# singIST: An integrative method for comparative single-cell transcriptomics between disease models and humans

**DOI:** 10.1371/journal.pcbi.1014002

**Published:** 2026-03-16

**Authors:** Aitor Moruno-Cuenca, Sergio Picart-Armada, Rachael Bogle, Jennifer Fox, Lam C. Tsoi, Johann Eli Gudjonsson, Alexandre Perera-Lluna, Francesc Fernández-Albert

**Affiliations:** 1 Data Science, R&D Center, Almirall SA, Sant Feliu de Llobregat, Spain; 2 B2SLab, Institut de Recerca i Innovació en Salut (IRIS), Universitat Politècnica de Catalunya, Barcelona, Spain; 3 Department of Dermatology, University of Michigan, Ann Arbor, Michigan, United States of America; 4 Networking Biomedical Research Centre in the subject area of Bioengineering, Biomaterials and Nanomedicine (CIBER-BBN), Madrid, Spain; 5 Institut de Recerca Sant Joan de Déu, Esplugues de Llobregat, Barcelona, Spain; The University of Texas MD Anderson Cancer Center, UNITED STATES OF AMERICA

## Abstract

**Motivation:**

Disease models are fundamental tools in drug discovery and early-stage drug development, but they only approximate human disease, and selecting a suitable model is challenging. Quantitative computational methods exist to assess molecular resemblance to human conditions, but approaching that work at single-cell resolution, and doing so in an explainable and generalizable way, remain very limited.

**Results:**

We present singIST, a computational method for comparative single-cell transcriptomics analysis between disease models and human conditions. singIST provides explainable quantitative measures on disease model similarity to the human reference at the pathway, cell type and gene levels. These measures jointly account for gene orthology, cell type presence in the model, cell type and gene importance in the human condition, and gene level fold changes in the model, within a unifying framework that controls for the intrinsic complexities of single-cell data. We first test singIST in three well-characterized murine models against moderate-to-severe Atopic Dermatitis, showing that it recapitulates established biology while generating new hypotheses. We then apply it to Hidradenitis Suppurativa, comparing in vivo human lesions with ex vivo skin explants with and without CD3/CD28 stimulation, and show that stimulation selectively improves pathways that already recapitulate the human signal. Finally, we perform simulation studies that: (i) unit-test the implementation and behaviour of the algorithm under controlled scenarios and (ii) compare singIST against a naïve baseline based on overlapping differentially expressed genes.

## Author summary

Animal, in vitro and ex vivo models are essential for understanding human disease and developing new treatments, but no model is a perfect copy of the human condition. Many disease mechanisms act in specific cell types, and current approaches do not fully exploit single-cell data to ask: how similar is this model to humans, and in which pathways and cell types? We developed singIST, a method that compares single-cell gene expression from disease models with human data and quantifies how well each model reproduces human disease at the level of pathways, cell types and individual genes. The outputs are designed to be interpretable, highlighting not only which model is closest to humans overall, but also which cell types and genes drive good or poor recapitulation. We first applied singIST to three mouse models with an atopic dermatitis-like phenotype and showed that it recovers known biology while pinpointing where each model diverges from human disease. We then used it in hidradenitis suppurativa, comparing human skin lesions with human skin explants with and without T-cell stimulation, and found that stimulation improves only those pathways that were already well-recapitulated without stimulation. Finally, simulation studies confirmed that the implementation behaves as expected and performs better than a simple baseline. Together, these results show that singIST can help researchers choose and interpret disease models in a more systematic and transparent way.

## 1. Introduction

Disease models are biological experimental systems to study human disease. These models are designed to mimic the pathophysiology, progression, and response to treatments observed in human conditions. These models serve as the backbone to drug discovery and early drug development activities; drug target validation and characterization [1]; compound screening [2,3]; preclinical studies to identify a lead candidate from several targets, select optimal formulation, posology and route of administration [4]; and guide early phase clinical trial design [5,6]. However, the validation of molecular physiology, aetiology and pathogenesis of disease models to that of human condition remain a challenge, contributing to high rates of drug development attrition [7].

Recently, there have been methodological advancements in bioinformatics to quantitatively assess the validity of disease models in mimicking a human condition, through bulk transcriptomics. Found In Translation (FIT) [8] is a statistical methodology, relying on regularized linear regression models, that leverages bulk transcriptomics data to extrapolate murine disease models’ gene expression to expression changes that would be equivalent in the human condition, by using disease models’ Fold Changes (FC). Another approach is In Silico Treatment (IST) [9], a computational method that assesses translation of disease-related bulk gene expression patterns between animal models and humans, by also simulating observed disease models’ FC in humans, providing an interpretable measure of their transcriptomics similarity. Nonetheless, evaluating disease models using bulk transcriptomics methods may lack the necessary granularity to underpin changes in specific cell populations involved in the pathological manifestations of the human condition. This is particularly true for Immune-mediated inflammatory diseases (IMIDs), whose pathogenesis is primarily driven by lymphoid cells [10,11]. Neither FIT nor IST provides a trivial approach to accommodate single-cell data. Current methodologies for comparative analysis of single-cell transcriptomic changes in disease models to that of human condition are scarce. A recurrent approach is to perform an Overlapping Differentially Expressed Genes (ODEGs) analysis between disease models and human condition [12–14], yet ODEGs have been proven to be suboptimal as this analysis treats every gene direction and magnitude as equal importance posing the need for more sophisticated approaches [15]. Another strategy is performing a dimensionality reduction technique (CCA, NNMF, tSNE) on disease models’ and human scRNA-seq data and comparing the obtained latent factors [16–18], which poses difficulty in interpreting and quantifying the similarity between both.

To address the challenges in single-cell transcriptomics analysis, we introduce singIST, a flexible computational method built on the foundation of IST. singIST facilitates comparative analysis between disease models and human conditions by accounting for orthology, cell type agreement, adaptive sparsity, and the importance of genes and cell types. It provides interpretable measures of transcriptomic similarity at different levels of granularity. We demonstrate the potential of singIST first in three well-characterized murine models against Atopic Dermatitis (AD), and then in a second use case: Hidradenitis Suppurativa (HS) skin explant cultures ex vivo with and without CD3/CD28 stimulation. In addition, we performed two simulation studies to verify the implementation and properties, and to compare singIST against a naïve ODEGs based approach.

## 2. Materials and methods

### 2.1 Materials

#### 2.1.1 Atopic Dermatitis: Evaluation of mouse models.

Moderate-to-severe AD patients single-cell RNA-seq data were obtained from [19], including 4 healthy control (HC) and 5 AD skin suction-blister samples. Cell types followed the original annotations (T cells, melanocytes, dendritic cells, Langerhans cells, and keratinocytes).

Three epicutaneously sensitized murine models that develop an AD-like eczematous phenotype were analysed: Oxazolone (OXA) and Imiquimod 5% cream (IMQ) from [20], and Ovalbumin (OVA) from [21]. For each model and its respective controls, three ear-skin biopsy replicates were available. Details on preprocessing, pseudobulk construction and cell-type harmonization across species, as well as GEO accession numbers and sample metadata, are provided in S1, S2, and S4 Files.

#### 2.1.2 Hidradenitis Suppurativa: Evaluation of human skin explants ex vivo.

Moderate-to-severe HS patient single-cell RNA-seq data were obtained from a previously published study of lesional and healthy control skin biopsies (8 HS and 8 HC biopsies; [22]). For singIST, we restricted the analysis to immune cell populations (myeloid cells, T-cells and mast cells). A detailed description of clinical characteristics, sequencing and preprocessing for this dataset is provided in S1 File.

In addition, we generated an ex vivo HS explant dataset. Six-millimetre punch biopsies from HS lesional skin and healthy control skin were allocated to three experimental groups: healthy control cultured with DMSO (HC DMSO), HS cultured with DMSO (HS DMSO), HS cultured with DMSO plus anti-CD3/CD28 antibodies (HS CD3/CD28). Each group included three independent biological replicates, and all biopsies were cultured for 60 hours before processing. Single-cell RNA-seq libraries were processed using the same pipeline as for the published HS dataset, and only myeloid cells, T-cells and mast cells were retained for analysis. Experimental details, preprocessing steps and sample-level information are described in S1 File.

#### 2.1.3 Pathway data.

For AD, we selected the pathways from [23] that were significantly enriched in moderate-to-severe AD versus HC, and retrieved the corresponding curated gene sets from MsigDB v7.5. For HS, pathways were selected based on GSEA enrichment in human HS versus HC skin. Database sources are provided in S1 File.

### 2.2 Methods

#### 2.2.1 singIST method.

The objective of this section is to introduce singIST framework for quantifying the agreement in single-cell transcriptomic changes between a human reference and a disease model. A summary of notation used throughout this section is shown in Table 1, and a graphical summary of the full singIST procedure is shown in Fig 2.

**Table 1:** Summary table of notations defined in singIST method. S1 File has further details on dimensions.

	Notation and Symbol	Description
**Symbol**	$\hat{e}$	Estimation of element *e*
	*e*′	Element *e* for human singIST treated samples
	$\underset{\sim }{e}$	Block of vectors or matrices *e*
	*e* ^*i*^	Element *i* of $\underset{\sim }{e}$, or variable containing information thereof
**Notation**	*C* ^*b*^	Cell type of interest *b*
	${𝒢}_{p}$; ${\tilde{𝒢}}_{p}$	Gene set of pathway *p*; Equivalent of ${𝒢}_{p}$ for disease model organism gene symbol
	${𝒢}_{p}^{b}$; ${\tilde{𝒢}}_{p}^{b}$	Gene subset of pathway *p* for cell type *b*, ${𝒢}_{p}^{b}\subseteq {𝒢}_{p}$; Equivalent of ${𝒢}_{p}^{b}$ for disease model
	${𝒫}^{p}$	Superpathway ${𝒫}^{p}={⋃}_{b=1}^{B}{𝒢}_{p}^{b}$
	$x_{ig}^{b}$	Human pseudobulk of gene $g\in {𝒢}_{p}^{b}$, sample *i* and cell type *b*
	$C^{b}$	Matrix with human pseudobulk for cell type *b*, $C^{b}=\{x_{ig}^{b}{\}}_{\begin{array}{c} 1\leq i\leq n\\ g\in {𝒢}_{p}^{b} \end{array}}$
	*Y*_*i*_	A binary variable that is 1 if human sample *i* is in target class, and 0 otherwise
	Y	Vector with human class $Y=\{Y_{i}{\}}_{\begin{array}{c} 1\leq i\leq n \end{array}}$
	$r_{\tilde{g}}^{b}$	Disease model FC between target and base class for disease model gene $\tilde{g}\in {\tilde{𝒢}}_{p}^{b}$
	$R^{b}$	Vector with disease model FC for cell type *b*, $R^{b}=\{r_{\tilde{g}}^{b}{\}}_{\tilde{g}\in {\tilde{G}}_{p}^{b}}$
	*y*_*i*_	Superpathway’s score of asmbPLS-DA for sample *i*
	Ω	Difference between the median values of *y*_*i*_ for target and base class
	$\gamma_{i}^{b}$	Cell type *b* contribution to *y*_*i*_ for sample *i*
	$\Gamma^{b}$	For cell type *b*, difference between the median values of $\gamma_{i}^{b}$ for target and base class
	$\delta_{ig}^{b}$	For cell type *b*, gene $g\in {𝒢}_{p}^{b}$ contribution to $\gamma_{i}^{b}$
	$\Delta_{g}^{b}$	For cell type *b*, difference between $\delta_{ig}^{b^{\prime }}$ and $\delta_{ig}^{b}$, which is constant for all samples *i*
	Ωf	$\Omega^{\prime }$ as a fraction of Ω
	$\Gamma f^{b}$	$\Gamma^{b^{\prime }}$ as a fraction of $\Gamma^{b}$
	$\Delta f_{g}^{b}$	$\Delta_{g}^{b}$ as a fraction of $\Gamma^{b}$

**Fig 1:**
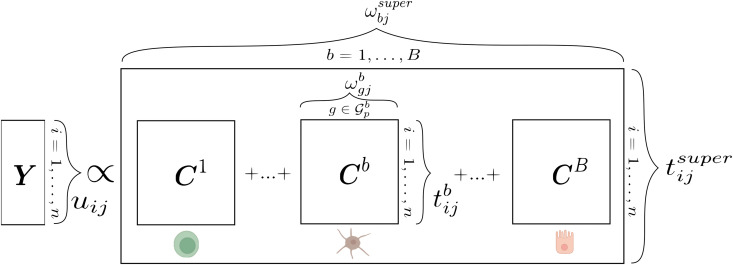
Representation of asmbPLS-DA for scRNA-seq readouts. The response vector Y contains samples as rows, one-hot encoded. Predictor blocks $C^{b}$ are defined by cell types, with columns representing genes and rows representing samples. Each element within these predictor blocks is the pseudobulk of gene expression values. The figure displays predictor blocks $\omega_{gj}^{b}$ and the predictor superblock $\omega_{bj}^{super}$ weights, as well as scores for the response matrix $u_{ij}$, predictor blocks $t_{ij}^{b}$, and the superblock $t_{ij}^{super}$. Created in BioRender. Moruno Cuenca, A. (2026) https://BioRender.com/l36t9i6.

We start by defining the three inputs of singIST: superpathways, human scRNA-seq, and disease model scRNA-seq $\log_{2}FC$. First, we define the concept of a superpathway ${𝒫}^{p}$ as a set containing cell types and genes. We name $C^{1},\ldots ,C^{b},\ldots ,C^{B}$ as the cell types of interest, previously identified and annotated. For each superpathway ${𝒫}^{p}$, there is a gene set ${𝒢}_{p}$ extracted from a pathway of interest *p*, from which gene subsets are derived for the cell types ${𝒢}_{p}^{1},\ldots ,{𝒢}_{p}^{b},\ldots ,{𝒢}_{p}^{B}$ where ${𝒢}_{p}^{b}\subseteq {𝒢}_{p}$ ∀b. Each superpathway is formally defined as ${𝒫}^{p}={⋃}_{b=1}^{B}{𝒢}_{p}^{b}$, with the complete set of superpathways $𝒫=\{{𝒫}^{1},\ldots ,{𝒫}^{p},\ldots ,{𝒫}^{P}\}$ representing all pathways under evaluation. singIST method runs independently for each of the superpathways; hence, without loss of generality, from now on we fix a superpathway ${𝒫}^{p}$.

Second, we structure the human scRNA-seq data according to the superpathway, after batch correction and normalization using standard workflows. Let $\underset{\sim }{C}=[C^{1},\ldots ,C^{b},\ldots ,C^{B}]$ be the block of matrices containing the pseudobulk log-normalized expression for each cell type. Each matrix is defined element-wise $C^{b}=\{x_{ig}^{b}{\}}_{\begin{array}{c} 1\leq i\leq n\\ g\in {𝒢}_{p}^{b} \end{array}}$, where $x_{ig}^{b}$ is the pseudobulk gene expression of human sample *i* for gene *g* in cell type *b*. We define the *target class* as the human experimental group that the disease model is intended to mimic (i.e., disease), and the *base class* as the human experimental group that should be differentiated from the *target class* (i.e., healthy control). Let Y be the response binary vector denoting human sample class, defined element-wise $Y=\{Y_{i}{\}}_{\begin{array}{c} 1\leq i\leq n \end{array}}$, where elements $Y_{i}\in \{0,1\}$

Third, we assume there are l=1,…,L disease models to be assessed against human scRNA-seq data for each superpathway. Since singIST runs independently for each disease model, without loss of generality we fix a disease model *l*. We structure the disease model scRNA-seq FC as b*l*ocks of vectors. Let $\underset{\sim }{R}=[R^{1},\ldots ,R^{b},\ldots ,R^{B}]$ be the block of vector containing the FC between *target class* and *base class* of disease model samples. Each vector is defined element-wise $R^{b}=\{r_{\tilde{g}}^{b}{\}}_{\tilde{g}\in {\tilde{G}}_{p}^{b}}$, where ${\tilde{G}}_{p}^{b}$ denotes the human gene subset ${𝒢}_{p}^{b}$ with its equivalent gene organism symbols for the disease model. The $\log_{2}FC$ are computed through Eq (1).


$$\begin{array}{c} r_{\tilde{g}}^{b}:=\left\{ \begin{array}{cc} 0 & {\text{p}}_{\tilde{g}}^{b}>0.05\\ log_{2}FC_{\tilde{g}}^{b} & {\text{p}}_{\tilde{g}}^{b}\leq 0.05 \end{array}\right.\; \end{array}$$ (1)

Where $\log_{2}FC_{\tilde{g}}^{b}$ (difference of mean $\log_{2}$ expression; i.e., $\log_{2}$ ratio of geometric means of *counts*+1) and $p_{\tilde{g}}^{b}$ are the disease model $\log_{2}FC$ and adjusted *p*-value, respectively, of *target class* versus *base class*. Details on the computation are in S1 File.

With the three inputs required for singIST now defined, we proceed to outline the steps of the method. The first step is to quantify the transcriptomic changes in the human reference for the superpathway, which comprises gene sets grouped by cell types. To accomplish this, we employ adaptive sparse multi-block partial least squares discriminant analysis (asmbPLS-DA) as the regression model, that performs feature selection in high-dimensional omics data using a multi-block structure [24]. A detailed description of asmbPLS-DA is provided in S1 File.

asmbPLS-DA is trained on human scRNA-seq Y as the response and $\underset{\sim }{C}$ as the block variable, both centred and scaled, with *base class* serving as the reference. The model structure is illustrated in Fig 1.

Using the fitted asmbPLS-DA, each human sample receives a superpathway score ${\hat{y}}_{i}$ – the continuous predicted response. The reference shift for the superpathway is the median difference between *target* and *base classes* (Eq. (2)), which quantifies how strongly the superpathway separates human classes. We call this measure *superpathway reference recapitulation*.


$$\hat{\Omega }:=\underset{i\in \{1\leq i\leq n|Y_{i}=1\}}{\text{median}}\left({\hat{y}}_{i}\right)-\underset{i\in \{1\leq i\leq n|Y_{i}=0\}}{\text{median}}\left({\hat{y}}_{i}\right)$$ (2)

We show that ${\hat{y}}_{i}$ admits an additive decomposition both by cell type and by gene (Eq. (3)). The full derivation is provided in S1 File.


$${\hat{y}}_{i}=\sum_{b=1}^{B}{\hat{\gamma }}_{i}^{b}=\sum_{b=1}^{B}\sum_{g\in {𝒢}_{p}^{b}}{\hat{\delta }}_{ig}^{b}$$ (3)

This result justifies defining cell type level human reference shift as the median difference of cell type contribution (${\hat{\gamma }}_{i}^{b}$) between *target* and *base* classes, which quantifies how strongly cell type *b* separates human classes. We call this measure *cell type b reference recapitulation*.


$${\hat{\Gamma }}^{b}:=\underset{i\in \{1\leq i\leq n|Y_{i}=1\}}{\text{median}}\left({\hat{\gamma }}_{i}^{b}\right)-\underset{i\in \{1\leq i\leq n|Y_{i}=0\}}{\text{median}}\left({\hat{\gamma }}_{i}^{b}\right)$$ (4)

The gene contributions ${\hat{\delta }}_{ig}^{b}$ serve to attribute the cell type effect hierarchically – i.e., within each cell type *b*, genes explain ${\hat{\Gamma }}^{b}$ additively and are later used for driver analyses of these shifts. In short, these reference recapitulation constructions provide a hierarchical quantification of the difference between human *target* and *base classes* – from superpathway ($\hat{\Omega }$) to cell type (${\hat{\Gamma }}^{b}$), with genes used for attribution.

In the second step of singIST, we ask a counterfactual question that links the disease model to the human reference data: if the human *base class* samples changed exactly as observed in the disease model, would the fitted asmbPLS-DA judge them more *target class*-like? However, directly comparing the disease model $\log_{2}FC$ to human scores is not meaningful because they live on different scales and may differ by organism. We therefore create a commensurate yardstick by injecting the disease model’s changes into human reference data – while holding the fitted asmbPLS-DA fixed. We call these samples *singIST treated samples* and we construct them by applying the disease model $\log_{2}FC$ to human expression *base class* samples only where a mapped cell type exists and there is a one-to-one ortholog; otherwise values are left unchanged (Eq. (5)).


$$\begin{array}{c} {x_{ig}^{b}}^{\prime }:=\ell \left(x_{ig}^{b},r_{\tilde{g}}^{b}\right)=\left\{ \begin{array}{cc} x_{ig}^{b} & \neg A\vee \neg B\\ x_{ig}^{b}+r_{\tilde{g}}^{b} & A\wedge B \end{array}\right.\; \end{array}$$ (5)

Two biologically plausible scenarios define the transformation in Eq (5); (¬A∨¬B) the case where either cell type *b* does not exist in the disease model (¬A) or disease model does not have a one-to-one ortholog of human gene *g* (¬B); (A∧B) the case where both cell type *b* and a one-to-one ortholog gene of *g* exist in disease model.

We now turn to the third step of singIST. Having constructed the singIST treated predictors ${\underset{\sim }{C}}^{\prime }=[C^{1^{\prime }},\ldots ,C^{b^{\prime }},$ $\ldots ,C^{B^{\prime }}]$ in step 2, we evaluate the counterfactual with the same fitted asmbPLS-DA from step 1 (identical loadings, scaling, and reference). Using the same derivations as in step 1, we summarize the disease model-induced shift relative to the *base class* human data.


$${\hat{\Omega }}^{\prime }:=\underset{i\in \{1\leq i\leq n|Y_{i}=0\}}{\text{median}}\left({\hat{y}}_{i}^{\prime }\right)-\underset{i\in \{1\leq i\leq n|Y_{i}=0\}}{\text{median}}\left({\hat{y}}_{i}\right)$$ (6)

$${\hat{\Gamma }}^{b^{\prime }}:=\underset{i\in \{1\leq i\leq n|Y_{i}=0\}}{\text{median}}\left({\hat{\gamma }}_{i}^{b^{\prime }}\right)-\underset{i\in \{1\leq i\leq n|Y_{i}=0\}}{\text{median}}\left({\hat{\gamma }}_{i}^{b}\right)$$ (7)

Which we call the *superpathway predicted recapitulation* and *cell type b predicted recapitulation*, respectively. These two measures quantify how far the *singIST treated samples* move toward the *target class* in the human reference space when judged by the human fitted asmbPLS-DA model.

In the last step, we report predicted recapitulations as percentage of the human reference recapitulations.


$$\hat{\Omega }f=100\cdot \frac{{\hat{\Omega }}^{\prime }}{\hat{\Omega }}$$ (8)

$$\hat{\Gamma }f^{b}=100\cdot \frac{{\hat{\Gamma }}^{b^{\prime }}}{{\hat{\Gamma }}^{b}}$$ (9)

To attribute cell type recapitulation to genes, we define the signed percent contribution of gene $g\in {𝒢}_{p}^{b}$:


$$\hat{\Delta }f_{g}^{\;b}=100\cdot \frac{{\hat{\Delta }}_{g}^{b}}{{\hat{\Gamma }}^{b}},\;\text{where}\;{\hat{\Delta }}_{g}^{b}:={\hat{\delta }}_{ig}^{b}\prime -{\hat{\delta }}_{ig}^{b}$$ (10)

The increment ${\hat{\Delta }}_{g}^{b}$ does not depend on *i*; a proof is provided in S1 File. By construction, $\hat{\Gamma }f^{b}=\sum_{{𝒢}_{p}^{b}}\hat{\Delta }f_{g}^{b}$, and therefore $\hat{\Delta }f_{g}^{b}$ partitions $\hat{\Gamma }f^{b}$ additively into gene level drivers. Genes without a mapped cell type, without a one-to-one ortholog, or with non-significant model $\log_{2}FC$ contribute zero by Eq. (5).

#### 2.2.2 Interpretation of recapitulation measures.

Recapitulation quantifies the concordance in both direction and magnitude of single-cell transcriptomic changes between the human reference and the disease model. A recapitulation score of $\hat{\Omega }f\approx 100$ indicates perfect agreement in direction and magnitude at the superpathway level, whereas a score of $\hat{\Omega }f\approx -100$ denotes an opposite direction but perfect agreement in magnitude. The interpretation for cell type recapitulation $\hat{\Gamma }f^{b}$ remains unchanged. Recapitulation is proportional to one-to-one orthology coverage, the number of mapped cell types, and the proportion of differentially expressed genes in the disease model. Within each cell type recapitulation $\hat{\Gamma }f^{b}$, gene contributions $\hat{\Delta }f_{g}^{b}$ indicate the direction and magnitude of each gene’s impact on the cell type recapitulation. Contributions are zero when the log2FC is non-significant, the gene lacks one-to-one orthology, or the cell type is unmapped. These properties are formally demonstrated in S1 File, and a comprehensive simulation study of recapitulation behaviour is presented in S5 File.

### 2.3 Validation methodology

#### 2.3.1 Validity test of the optimal asmbPLS-DA.

Once the optimal model is selected, the validity of such for classifying between *target class* and *base class* is checked by a permutation test [25] adapted to small sample size and asmbPLS-DA. A null model distribution $H_{0}:Y\underset{\sim }{C}$ is generated by permuting Y, noted as σ(Y), and setting the number of permutations. For each σ(Y) an asmbPLS-DA model is fitted with $J^{*}$ and $\lambda_{j}^{b}$ as the optimal number of PLS components and quantile combination for each block and PLS component, respectively, and randomly taking one sample out to avoid overfitting. With the permuted model, *Y* is predicted for all samples under analysis, such prediction is compared against the true *Y* by *F*_1_ score. The rationale behind randomly permuting each *Y* element is that the original relationship of the model is disrupted while the dependence structure of $\underset{\sim }{C}$ is preserved [26], thus providing a control of a false positive model. If the optimal model is actually significant, it is expected that error measures increase substantially when permuting. To this end, the *F*_1_ LOOCV error of optimal model is compared against the $CI_{\left(1-\alpha \right)}$ of null distribution of *F*_1_ score, where α=0.05 is the confidence threshold and the quantile serves as the p-value which is adjusted for multiple comparison by Benjamini-Hochberg [27]; FDR is set to 0.1.

#### 2.3.2 Parameter variabilities and significance of the optimal asmbPLS-DA model.

Cell type and gene importance, within a cell type *b*, may be assessed by considering the weighted average of its estimated coefficient, by taking as weights the relative importance of each PLS component *q*_*j*_ [28]. To this end, we define the Cell Importance Projection (CIP) for cell type *b*:


$$CIP^{b}=\frac{\sum_{j=1}^{J^{*}}q_{j}{\left(\omega_{bj}^{super}\right)}^{2}}{\sum_{j=1}^{J^{*}}q_{j}}\;]$$ (11)

Similarly we define the Gene Importance Projection (GIP) for gene *g* within cell type *b*:


$$GIP_{g}^{b}=\frac{\sum_{j=1}^{J^{*}}q_{j}{\left(\omega_{gj}^{b}\right)}^{2}}{\sum_{j=1}^{J^{*}}q_{j}}\;]$$ (12)

Both indices verify $\sum_{b=1}^{B}CIP^{b}=\sum_{g\in {𝒢}_{p}^{b}}GIP_{g}^{b}=1$; this is proven in S1 File. The direction of *CIP*^*b*^ may be assessed by $sign(CIP^{b})=sign(\sum_{j=1}^{J^{*}}q_{j}\omega_{bj}^{super})$, and equivalently for $GIP_{g}^{b}$. Note that *CIP*^*b*^ distribution is nested to the already estimated $\lambda_{j}^{b}\in [0,1]$, the blocks with only a small number of relevant genes will assign higher $\lambda_{j}^{b}$ values, being a cell type $\lambda_{j}^{b}=1$ a cell type that does not contain any relevant information in classifying between *target class* and *base class*.

The $GIP_{g}^{b}$ distribution of a gene that is significant is likely to substantially differ from its associated null *H*_0_ distribution. A null distribution of $GIP_{g}^{b}$ of the form $H_{0}:x_{i_{1}g_{1}}^{b}x_{i_{2}g_{2}}^{b},\forall i_{1}\neq i_{2},\forall g_{1}\neq g_{2}$, and b∈{1,…,B} is generated by permuting all samples and genes within blocks. Note that permuting between blocks would not satisfy exchangeability assumption as $GIP_{g}^{b}$ distribution is dependent on $\lambda^{b}$. The median $GIP_{g}^{b}\sim {𝒟}_{g,Jackknife}^{b}$ of distribution of the optimal model is compared against the null distribution $GIP_{g}^{b}\sim {𝒟}_{H_{0}}^{b}$ by a Mann-Whitney U test, with the alternative hypothesis being the median ${𝒟}_{g,Jackknife}^{b}$ greater than median of ${𝒟}_{H_{0}}^{b}$. P-value is adjusted by Bonferroni correction with a lower bound of expected number of true null hypothesis for each cell type $m_{0}^{b}=\lfloor \prod_{j=1}^{J^{*}}\lambda_{j}^{b}|{𝒢}_{p}^{b}|\rfloor$; the rationale is provided in S1 File.

## 3. Results

### 3.1 Atopic Dermatitis: Evaluation of mouse models

#### 3.1.1 Training on human data and reference shifts.

Training the human reference on AD versus healthy yielded clear superpathway separation (FDR ≤0.1), with gene set sizes spanning from 15 genes (CD40/CD40L signalling [PID]) to 701 (Cytokine signalling in the immune system [REACTOME]) (Table A in S3 File).

Using the CIP measure, each superpathway exhibited a distinct ranking of cell types that most strongly drive the AD-healthy prediction. For Dendritic Cells in Th1/Th2 Development [BIOCARTA], T-cells had the highest CIP, with secondary importance in melanocytes and dendritic cells, while keratinocytes and Langerhans cells ranked lower. JAK-STAT signalling pathway [KEGG] displayed diffuse CIP profile (similar importance across cell types), consistent with broad activation. Chemokine receptors bind chemokines [REACTOME] was led by keratinocytes and dendritic cells, whereas Cytokine-cytokine receptor interaction [KEGG] was driven primarily by antigen-presenting cells (APCs). Full CIP ranks, sparsity parameters, and the number of significant genes per cell type are reported in Table A in S3 File.

Gene level signals align with canonical AD biology and added cell type specific detail, with explicit literature cross-references in Table C in S3 file. In Dendritic Cells in Th1/Th2 Development [BIOCARTA], T-cells showed upregulation of IL13, IL5, and CSF2 with TLR7 down, and APCs highlighted ANPEP. In JAK-STAT signalling pathway [KEGG], T-cell interleukins (e.g., IL13, IL26, IL2RA, IL7) and interferon-axis components were prominent; keratinocytes showed IL15/IL15RA; dendritic cells emphasized SPRED1, SOCS1, and OSM; and melanocytes exhibited concurrent upregulation of CCND3 and CCND1, consistent with proliferative JAK-STAT activity (see S2 File for literature references). Per superpathway and cell type top-give genes are listed in Table B in S3 File.

#### 3.1.2 Recapitulation across Oxazolone, Imiquimod, and Ovalbumine mouse models.

Fig 3 summarizes per-model one-to-one orthology to human and the corresponding superpathway recapitulation estimated by singIST. All three are mouse models; observed one-to-one orthology levels are broadly similar, but OVA shows lower observed orthology because fewer genes were sequenced per pathway. Coverage varies by pathway – for example, Asthma and Chemokine receptors bind chemokines are 60%, whereas IL12 signalling events mediated by STAT4 reach 100% (Fig A in S3 File).

**Fig 2:**
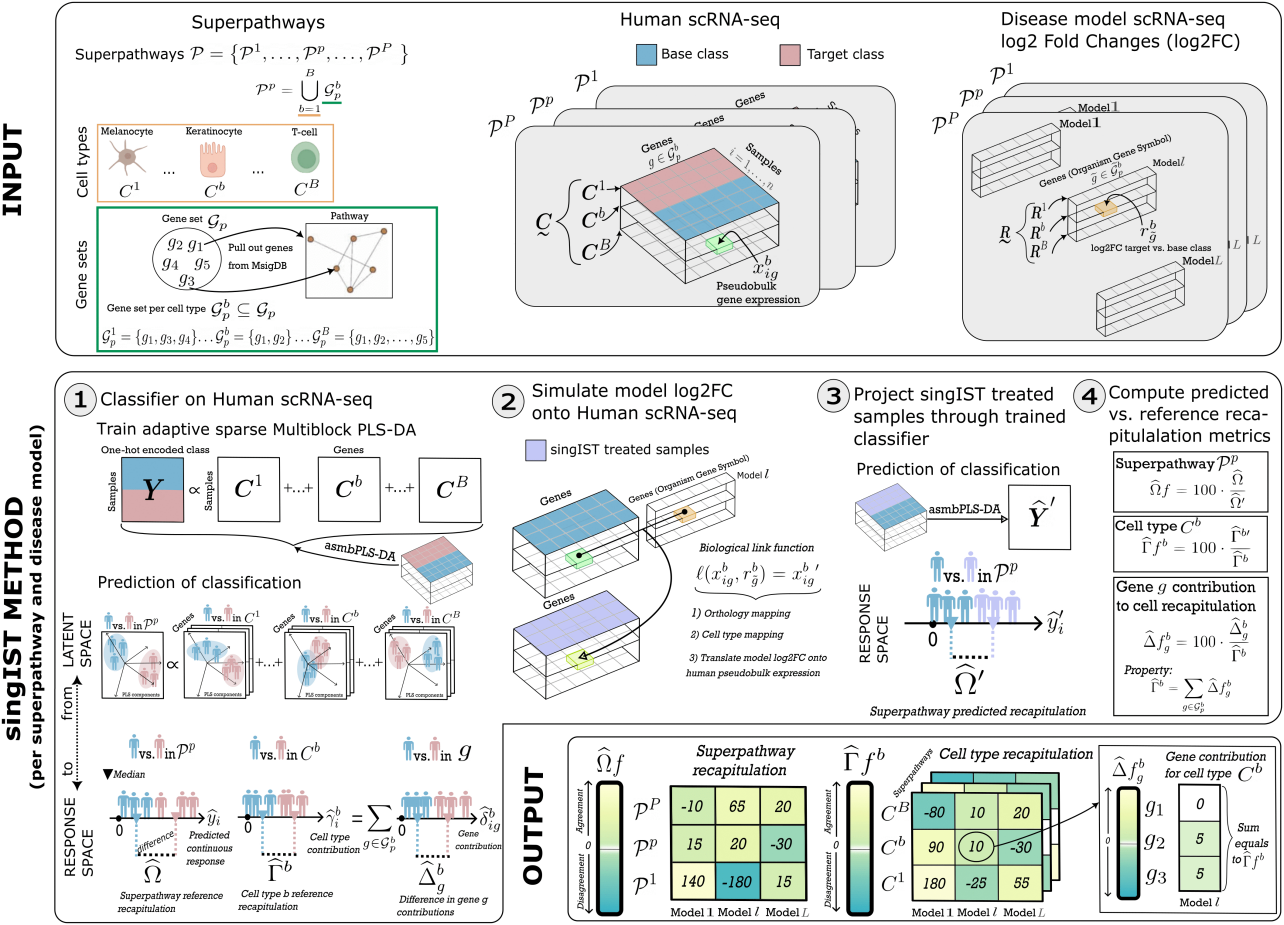
INPUT) First, definition of a superpathway. (${𝒫}^{p}$) as a set containing cell types and genes. For each ${𝒫}^{p}$, there is a gene set ${𝒢}_{p}$ from which gene subsets are derived for cell types $\{{𝒢}_{p}^{b}{\}}_{b}\subseteq {𝒢}_{p}$. Second, for each ${𝒫}^{p}$ human scRNA-seq data are organized into matrix layers. Target class is the human experimental group that the disease model aims to mimic (i.e., disease), while base class is such that it should be differentiated from target class (i.e., healthy control). Third, for each ${𝒫}^{p}$ disease models scRNA-seq $\log_{2}FC$ are structured into vector layers. **singIST METHOD)** The method is organized into four steps, which run independently for each ${𝒫}^{p}$ and disease model. **Step 1)** *Objective:* Quantify differences between target and base class human samples at various levels of granularity (superpathway, cell type, and gene) using asmbPLS-DA. *Input:* A ${𝒫}^{p}$ and human scRNA-seq data. *Output:* Optimal asmbPLS-DA. From such, we derive cell type contributions (${\hat{\gamma }}_{i}^{b}$) and gene contribution (${\hat{\delta }}_{ig}^{b}$). With the contributions we compute similarity measures at the superpathway ($\hat{\Omega }$) and the cell type levels (${\hat{\Gamma }}^{b}$). **Step 2)** *Objective:* Biologically unify the human data with the disease model data for subsequent comparison. *Input:* Human scRNA-seq base class samples and disease model scRNA-seq $\log_{2}FC$ data. *Output:* Human scRNA-seq gene expression observed when disease model $\log_{2}FC$ are applied, we call them *singIST treated samples*. The former is achieved in the *Biological link function*, which performs steps; one-to-one orthologous mapping; cell type alignment; translate $\log_{2}FC$ to $x_{ig}^{b}$. **Step 3)** *Objective:* Compute metrics of output from Step 1 between singIST treated samples and human scRNA-seq base class. *Input:* singIST treated samples, Human scRNA-seq base class samples and optimal asmbPLS-DA. *Output:* Pathway predicted recapitulation (${\hat{\Omega }}^{\prime }$), Cell type *b* predicted recapitulation (${\hat{\Gamma }}^{b}$) and predicted gene contributions (${\hat{\delta }}_{ig}^{b^{\prime }}$). **Step 4)** *Objective:* Compute similarity metrics between human and disease model. *Input:* From step 1; $\hat{\Omega }$ and ${\hat{\Gamma }}^{b}$, and ${\hat{\delta }}_{ig}^{b}$. From step 3; ${\hat{\Omega }}^{\prime }$ and ${\hat{\Gamma }}^{b^{\prime }}$, and ${\hat{\delta }}_{ig}^{b^{\prime }}$. *Output:* Predicted recapitulations as a fraction of the reference recapitulations ($\hat{\Omega }f$, $\hat{\Gamma }f^{\;b}$). $\hat{\Gamma }f^{\;b}$ is explained by contributing genes ($\hat{\Delta }f_{g}^{b}$), providing interpretation on which genes drive the cell type recapitulation. **OUTPUT)** $\hat{\Omega }f$ and $\hat{\Gamma }f^{b}$ are displayed. Positive values show agreement in gene expression change between disease model and humans; negative values show opposition. Each $\hat{\Gamma }f^{\;b}$ equals the sum of its gene contributions $\hat{\Delta }f_{g}^{b}$. Fig 2.

**Fig 3:**
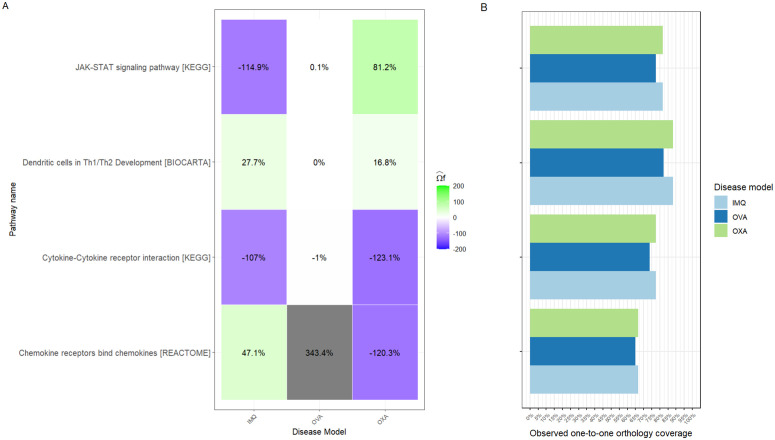
Superpathway recapitulation and observed one-to-one orthology of AD disease models. **A)** Superpathway predicted recapitulation as a fraction of the superpathway reference recapitulation for IMQ, OXA and OVA across all pathways under study. Negative recapitulations refer to opposed directions with human observed condition, while positive recapitulations define agreement in direction. **B)** Observed one-to-one orthology coverage refers to number of observed and one-to-one ortholog genes in disease model as a fraction of pathway gene set size. Despite the fact that disease models belong to the same organism *mus musculus* their differences in observed orthology one-to-one coverage come from sequenced reads.

**Fig 4:**
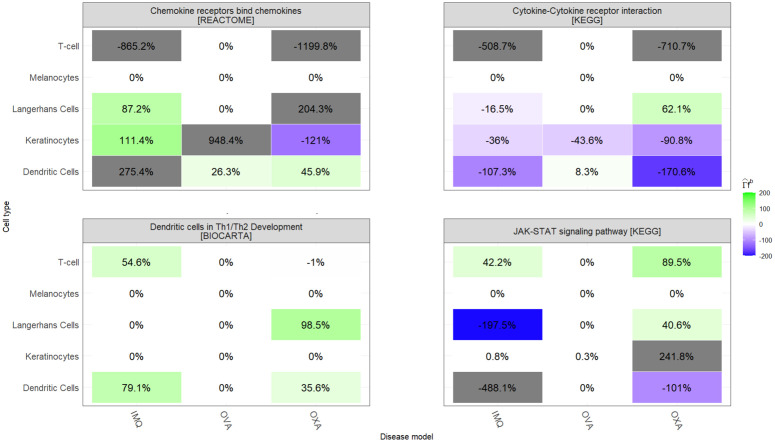
Cell type recapitulation for all AD disease models and superpathways under analysis.

**Fig 5:**
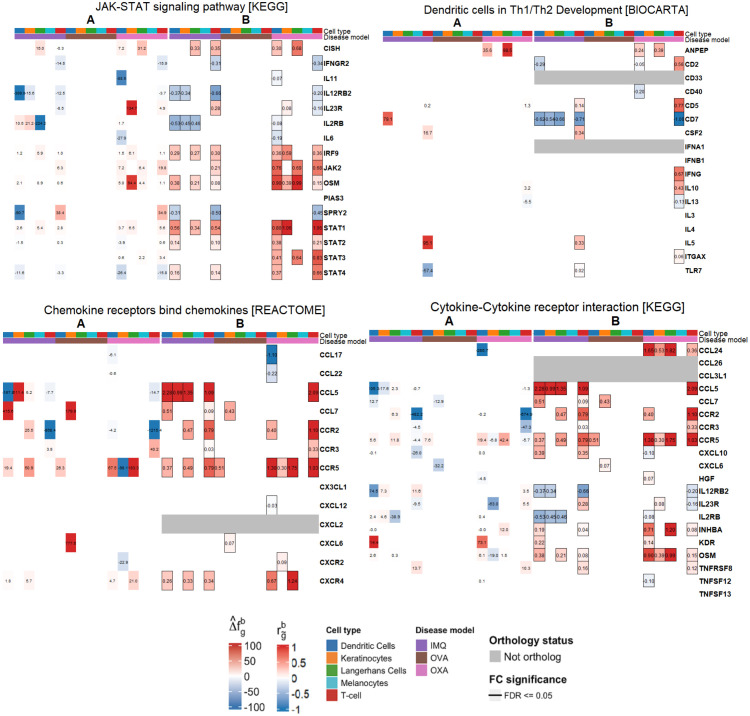
Gene contribution and disease model estimated. $r_{\tilde{g}}^{b}$.**A)** Gene contribution to cell type recapitulation by disease model. If gene set size of pathway is greater than 50, only the top 5 contributing genes, for each cell type, were displayed. Blank gene contributions correspond to 0 values. **B)** Computed $r_{\tilde{g}}^{b}$ by disease model. Grey $\log_{2}FC$ refer to genes without one-to-one ortholog and/or not sequenced in disease model. Framed $\log_{2}FC$ refer to statistically significant FDR≤0.05 genes, as per FindMarkers. Blank $\log_{2}FC$ correspond to 0 values.

Superpathway recapitulation for Dendritic Cells in Th1/Th2 Development was moderate for IMQ (27.7%) and OXA (16.8%), and null for OVA (0%). Cell type recapitulations are displayed in Fig 4, which shows moderate T-cell recapitulation for IMQ (54.6%) and no recapitulation for OVA (0%) and OXA (-1%). In OXA, the signal is instead carried by Langerhans cells (98.5%) and dendritic cells (35.6%). OVA has 0% for Langerhans cells across superpathways because that population was removed (<100 cells). Melanocytes are 0% in all models and superpathways (unmapped cell type). Fig 5 shows gene contributions to cell type recapitulation. In IMQ, T-cell recapitulation is driven by IL5 (95.1%) and CD7 (16.7%), whereas TLR7 contributes negatively (-57.4%) because it is suppressed in human AD but upregulated in IMQ. OVA has no DEGs in this pathway and therefore 0% recapitulation. Notably, the key T-cell marker IL13 in AD does not contribute positively in any model. JAK-STAT signalling pathway [KEGG] superpathway recapitulation was highest for OXA (81.2%), while IMQ (-114.9%) was opposite in direction and OVA (0.1%) was essentially null. OXA agreed in direction across all cell types except dendritic cells, with varying magnitudes. The negative score for IMQ was driven by strong discordance in Langerhans cells (-197.5%) and dendritic cells (-488.1%); gene-level contributions point to IL12RB2 in dendritic cells (-399%) and IL2RB in Langerhans cells (-224.2%). Cytokine-cytokine receptor interaction [KEGG] recapitulation was strongly negative for all models, with OVA ≈0%. The disagreement was consistent across cell types. Gene-level disagreement drivers included multiple chemokine receptors (CCR2, CCR3) and ligands (CCL5, CCL24, CCL17, CXCL10, CXCL6). Chemokine receptors bind chemokines [KEGG] recapitulation was high for OVA (343.4%), moderate for IMQ (47.1%) and negative for OXA (-120.3%). The OVA signal is heavily concentrated in keratinocyte cell type, just two genes - CCL7 (179.9%) and CXCL6 (777.6%) - which largely accounts for its extreme value.

### 3.2 Hidradenitis Suppurativa: Evaluation of human skin explants ex vivo

#### 3.2.1 Training on human data and reference shifts.

We first trained the human reference model on lesional HS skin versus healthy control skin. All superpathways for HS corresponded to immune and inflammatory infiltration of the dermis (T-cell activation/signalling, chemokine–cytokine signalling, myeloid programmes), and all of them were statistically significant under the permutation test (FDR ≤ 0.1), confirming that the disease and control separation is not due to chance (Fig D in S3 File).

Cell type importance projection (CIP) showed two recurrent patterns (Figs E and F in S3 File). A first group of superpathways was predominantly T-cell driven, with T-cells ranking clearly highest and myeloid cells contributing little. A second group showed a joint T-cell and myeloid signature, in which T-cells remained the top contributor but myeloid cells also carried a sizable part of the HS signal. Mast cells were not predictive in any superpathway, consistently appearing with negligible CIP. These superpathways define the human HS reference shifts that we later use to evaluate ex vivo explants with and without CD3/CD28 stimulation.

#### 3.2.2 Recapitulation with and without CD3/CD28 stimulation in explants.

Since both organisms are human, observed one-to-one orthology was uniformly high (mostly >90%) for HS explants and controls; the small differences between disease models were due to genes not detected in the explants rather than to mapping issues (Fig G in S3 File).

**Fig 6:**
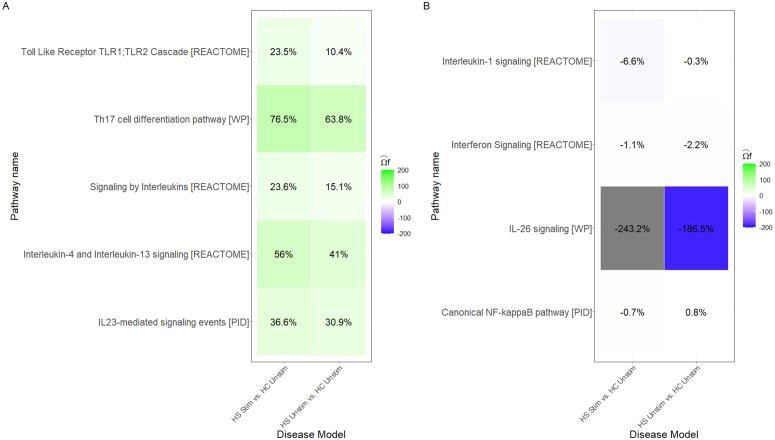
Superpathway recapitulation of HS disease models. **A)** Superpathway recapitulation of well-recapitulated superpathways in HS disease models (HS Unstim vs. HC Unstim and HS Stim vs. HC Unstim). **B)** Superpathway recapitulation of bad-recapitulated superpathways in HS disease models.

In the unstimulated explants (HS in DMSO vs. Healthy in DMSO), singIST already identified a group of superpathways that reproduced the human HS signal (Fig 6A). These corresponded mainly to T-cell driven pathways and to mixed T-cell/myeloid pathways associated with dermal inflammation (Fig H in S3 File). When the same explants were stimulated with CD3/CD28, these pathways were the ones that improved: their superpathway recapitulation increased, and the gain was also visible in the T-cell and myeloid components and in the gene-level contributions (Fig 6A; Figs I and J in S3 File). In other words, stimulation amplified the pathways that were already aligned with the human reference.

**Fig 7:**
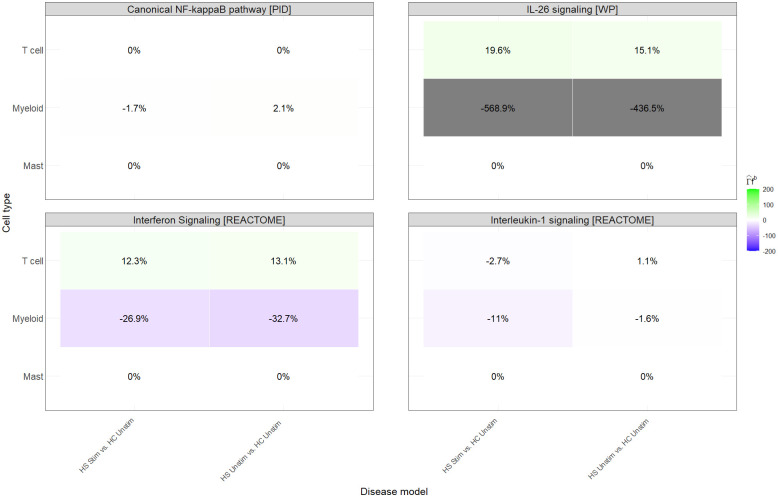
Cell type recapitulation for bad-recapitulated HS disease models.

By contrast, the superpathways that were not well recapitulated in the unstimulated condition did not become more human-like after stimulation (Fig 6B). This was especially clear for pathways whose human signal was predominantly a combination of T-cell and myeloid (Fig H in S3 File) or where explants showed an opposite direction in myeloid cells: even after CD3/CD28, cell-type recapitulation remained low or fragmented (Fig 7; Fig K in S3 File). The only clear exception was NF-*κ*B signalling, which is T-cell driven in vivo but was still poorly reproduced ex vivo. This pattern is consistent with the $\log_{2}FC$ heatmaps: stimulated and unstimulated explants display very similar transcriptomic profiles, and stimulation mainly produces a stronger version of the same response, rather than introducing a new one (Fig J and K in S3 File). At the same time, stimulation does what we would expect biologically in T-cells: ZAP70 activation marker that was absent in the unstimulated cultures appears after CD3/CD28, becoming a positive contributor to the Th17 cell differentiation [WP] superpathway, i.e., exactly in one of the pathways that improved under stimulation.

An independent analysis of cell type composition showed that myeloid cells are expanded in vivo HS lesions but reduced in the unstimulated explants (Fig L in S3 File), which explains why myeloid driven HS pathways remain poorly recapitulated in the explants.

## 4. Discussion

Atopic Dermatitis (AD) represents a chronic skin-immune-mediated inflammatory disease (IMID), characterized by dysregulated T-cell mediated inflammation and keratinocyte differentiation [29]. We put a special focus on the discussion on pathways proven to be causal drivers of AD pathogenesis or related to its clinical severity; JAK-STAT signalling pathway, Dendritic Cells in regulating Th1/Th2 development, Cytokine-Cytokine receptor interaction, and Chemokine signalling pathway. Of the four AD pathways we examined, JAK–STAT signalling [KEGG] was the only one showing broad activation across cell types (TC, KC, MC, and APC), whereas the other pathways were activated in a more cell type–restricted manner. This is compatible with lesion associated inflammatory signalling in AD skin and with the concurrent upregulation of cell-cycle and survival genes reported in skin lesions, including the simultaneous activation of CCND3/Cyclin-D3 and CCND1/Cyclin-D1 in MC [30], as well as genes in the PI3K–AKT axis such as PIK3 CD and PIK3CB, and the upregulation of IL15 and IL15RA/IL15Rα in KC during inflammation.

It was not unexpected to observe few DEGs in OVA for the pathways analysed. This agrees with the low DEG counts in TC and DC reported by [21] and with bulk-RNA studies showing limited differential expression in AD-relevant pathways in this model [31]. The absence of MC in all three mouse models is expected, because in C57BL/6J and BALB/c pelage — including the ear, where biopsies were collected — largely lacks functional pigment producing melanocytes [32].

The Dendritic cells in Th1/Th2 development [BIOCARTA] pathway was the one with disease model and human mismatch. In IMQ, TLR7 is upregulated in T-cells, but in human AD it is suppressed, so TLR7 enters singIST with a negative contribution. This fits biology: TLR7 signalling drives Th1/Th17 responses [33], whereas its suppression favours the Th2 profile seen in AD [34]. A second mismatch is that none of the three mouse models induces IL13/IL4 through Th2 cells: IMQ shifts to IL17/IL22, and OVA/OXA produce IL13/IL4 mainly through infiltrating basophils/myeloid cells rather than Th2 cells [20,21]. Consequently, classical Th2 genes contribute little or even slightly negatively (e.g., IL13 in OXA with -5.5% contribution in T-cells).

In JAK–STAT signalling [KEGG], OXA showed the highest human-like shift (81.2%), with consistent agreement across cell types except dendritic cells, which were opposed in IMQ/OXA models. OVA was essentially null (0.1%), and IMQ was the opposite (−114.9%). This pattern is consistent with OXA being the murine model most often used for JAK-inhibitor studies in AD [35] and with bulk RNA data favouring OXA over OVA for this pathway [31]. One source of disagreement is STAT4, which is suppressed during Th2 development in human AD [36] but upregulated in IMQ and OXA, reflecting their non-Th2-skewed responses, and thus STAT4 contributing negatively to T-cell recapitulations.

Chemokine receptors bind chemokines [REACTOME] and Cytokine–cytokine receptor interaction [KEGG] share several high contributing genes. Many of these chemokines are stage-dependent in AD lesions. In our data, CCL5 (from the chemokine receptor pathway) showed large negative contributions in TC for both IMQ and OXA (and in DC for IMQ), whereas acute AD lesions are known to upregulate CCL5, while chronic lesions are known to suppress it [37]. Since the human samples [19] are likely chronic and the murine lesions are acute, this stage mismatch potentially explains the opposite directions across models and cell types.

In the HS application, singIST showed very consistent behaviour: pathways that already recapitulated the human HS signal in unstimulated explants were the ones that improved after CD3/CD28, whereas poorly recapitulated pathways stayed poor.

For the well recapitulated pathways, CD3/CD28 produced the expected boost: superpathway scores increased, the same cell types gained resemblance, and the same genes became stronger contributors (Fig 6A; Fig H and J in S3 File). This is the pattern we would expect after CD3/CD28, and it also shows that the stimulation was effective: ZAP70, an early marker of T-cell activation [38], appears in T-cells only in the stimulated explants in Th17 cell differentiation pathway [WP]. This means that the fact that most transcriptomic profiles remain very similar between unstimulated and stimulated conditions is not due to insufficient stimulation, but to the biology of the explant system (i.e., it can boost the T-cell component, but it cannot rebuild the full missing signals). By contrast, myeloid dependent HS pathways did not improve with stimulation (Fig 6B). Even after CD3/CD28, explants did not recover the human like myeloid signal, and at the cell-type level recapitulation remained fragmented (Fig 7; Fig K in S3 File). This agrees with reports showing that tissue-resident macrophages and microglia rapidly lose their tissue-imprinted transcriptional programme once they are placed in culture and only re-acquire it in the appropriate in vivo niche or under specific stimuli [39–41]. Our independent cell type composition analysis points in the same direction: in vivo HS skin showed an expansion of myeloid cells, whereas the unstimulated explants in culture showed a significant reduction (Fig L in S3 File). In other words, the culture condition preserves T-cell responsiveness to stimulation, but does not preserve the tissue-conditioned myeloid cells that drive part of the HS signature. Taken together, this supports a simple claim: the CD3/CD28-stimulated HS explant is the most appropriate model for those HS pathways that already recapitulate in the unstimulated condition, but not for myeloid driven HS pathways that do not.

While demonstrating significant capabilities, singIST presents several limitations, including dependence on pre-annotated cell types, the assumption of homogeneous effects when translating fold changes to human gene expression, and the requirement for well-defined human disease states (e.g., endotypes) prior to analysis. Additionally, extensions could be explored on differentiating changes due to cell type-specific gene expression and cell type proportions, between human classes. Further validation in additional disease contexts will solidify its utility in drug development and preclinical research.

## 5. Conclusions

Here we developed singIST, a computational framework for comparative single-cell transcriptomics that provides an integrative, explainable way to evaluate disease model similarity to human conditions at pathway, cell type and gene levels. Its application to murine models against Atopic Dermatitis and to human Hidradenitis Suppurativa skin explants (with and without CD3/CD28 stimulation), together with supporting simulation studies, shows that singIST can both recover known biology and pinpoint where models succeed or fail to recapitulate human disease.

## Supporting information

S1 File Materials and methods. Detailed description of methodological procedures, model formulation, and data processing steps.
(PDF)

S2 File Sample metadata. Metadata for all human and mouse samples, including demographics, experimental conditions, sequencing protocol, and GEO accession numbers.
(PDF)

S3 File Extended results. Supplementary tables and figures of Results section.
(PDF)

S4 File Cell type mapping. Overview of the cell type harmonization between human data and mouse models, including cluster selection and mapping criteria.
(PDF)

S5 File Simulation study. Simulation studies to validate singIST recapitulation properties and benchmark against ODEGs.
(PDF)
