# Supplementary material for: singIST: An integrative method for comparative single-cell transcriptomics between disease models and humans
Source: PLoS Comput Biol. 2026 Mar 16;22(3):e1014002. doi: 10.1371/journal.pcbi.1014002 (PMC13008255; doi:10.1371/journal.pcbi.1014002)
Supplement: S1 File — Detailed description of methodological procedures, model formulation, and data processing steps. (PDF) [file pcbi.1014002.s001.pdf]

Supplementary Material S1: Materials and methods

1. MATERIALS SUPPLEMENTARY

A. Atopic Dermatitis use case data

All disease models, and their respective controls, have 3 replicates of ear skin biopsies. To enable comparison with human data, existing cell-type annotations in the mouse datasets were mapped onto the human cell-type panel (TC, MC, DC, LC, KC); the mapping between original and harmonized labels is shown in Supplementary Material S2. For both human and murine datasets, raw UMI counts were aggregated into pseudobulk profiles per sample and cell type and log-normalized using Seurat v5.0.1. Data were used as provided in original sources. All scRNA-seq data were generated on the 10x Genomics platform. GEO identifiers and sample metadata are listed in Supplementary Material S4.

B. Hidradenitis Suppurativa use case data

Hidradenitis Suppurativa (HS) is a debilitating chronic inflammatory disease characterized by chronic abscess formation and development of multiple draining sinus tracts in the groin, axillae, and perineum.

Human data were extracted from GSE154775 and GSE173706. Five patients with chronic HS and 8 healthy controls for single-cell analysis at the University of Michigan. HS patients had a disease duration of at least 1 year prior to sampling and Hurley stage II or III disease. Patients did not use biologics or i.v. treatment and were off any other systemic treatment and off any topical agents for at least 2 weeks prior to inclusion. Six-millimeter punch biopsies were taken from lesional skin in the case of HS patients and healthy control skin from the hip/buttock for healthy controls.

For the HS explant, single cell RNA sequencing was performed using 10X Genomics Flex-seq kit. Tissue was dissociated per the manufacturer's instructions using the gentleMACS Octo Dissociator. In brief: 50um scrolls were placed into a GentleMACS C-tube (Miltenyi BioTech). The scrolls were then deparaffinized, washed and dissociated into a single cell suspension enzymatically using Liberase TH. Reads were aligned to the GRCh38 genome. CellRanger 7.0.1 was used to generate the gene x cell matrix. Matrices were loaded into Seurat v4.3.0. Ambient RNA was removed using SoupX v1.6.2 using default settings. ScDblFinder v1.12.0 was used to remove doublets using default settings. Cells containing fewer than 200 UMI and more than 25% mitochondrial reads were removed. Seurat v4.3.0 was used to normalize, scale, and reduce dimensionality of the data using Uniform Manifold Approximation and Projection (UMAP). Batch correction was performed using harmony v 1.0, using donor as batch. Clusters were annotated manually using a list of literature-based marker genes.

C. Pathway data

Pathways for the Atopic Dermatitis (AD) analyses were derived from the serum proteomics study of Brunner et al. (2017) (moderate-to-severe AD vs Healthy controls). We started from the set of pathways reported as significantly enriched in that work and retained those that: were available as gene sets in MsigDB v7.5, and belonged to the curated (C2) collections; archived sets were excluded. Gene sets were retrieved from MsigDB version 7.5 (Liberzon et al., 2015), pathway database source encompasses: KEGG (Kanehisa et al., 2023), REACTOME (Gillespie et al., 2022), BioCarta (Nishimura, 2001), PID (Schaefer et al., 2008), WP (**wikipathways**). After filtering, 22 pathways were retained and used to train the human AD reference and to evaluate the murine AD models. In total, 22 gene sets satisfying the former criteria were considered.

To define the HS pathways, we performed a gene set enrichment analysis on the in vivo HS biopsy dataset (HS lesional vs. Healthy control skin) at the cell type specific level. For each cell type, we first constructed a pseudobulk count matrix by aggregating raw counts across cells for each sample-cell type combination. For a given cell type, samples were labeled as HS or control, and we fitted a DESeq2 model to obtain $\log_2 FC$ estimates for HS versus control. The resulting

statistics were used to build a ranked gene list (genes ordered by $\log_2 FC$, decreasing) for that cell type; only cell types with both HS and control samples were retained for downstream analysis. Gene sets were taken from MsigDB v7.5 (C2 curated collections). Enrichment was performed with the GSEA function from clusterProfiler, using the ranked $\log_2 FC$ vectors as input, TERM2GENE defined by MsigDB mapping, nPermSimple = 100000 and a nominal p-value cutoff of 0.05. For each cell type, we exported the full GSEA results and visualised the top enriched pathways.

Pathways selected for the HS singIST analysis correspond to those enriched in HS versus control, for at least one cell type, and that are immune related.

2. METHODS SUPPLEMENTARY

A. Notation of singIST

Here we further detail dimensions of notation stated in Table 1 (Section 2.2.1). Throughout, we fix one superpathway P^p (we omit the superscript p when clear) and consider B cell types indexed by $b \in \{1, \ldots, B\}$. Let n denote the number of human samples, indexed by $i \in \{1, \ldots, n\}$.

Gene sets and indices. For the chosen pathway p, let G_p be the pathway gene set (human gene symbols) and $\bar{G}_p$ its equivalent gene set in the disease-model organism gene symbols. For each cell type b, we define a cell-type-specific gene subset $G_p^b \subseteq G_p$ and its disease model equivalent $\bar{G}_p^b \subseteq \bar{G}_p$. We write $|G_p^b|$ and $|\bar{G}_p^b|$ for the corresponding cardinalities (which may vary across b).

Human pseudobulk matrices. For each cell type b, the human pseudobulk matrix is

$$C^b \;=\; \{x_{ig}^b\}_{1 \le i \le n,\, g \in G_p^b} \in \mathbb{R}^{n \times |G_p^b|},$$

where each row corresponds to a human sample i and each column corresponds to a gene $g \in G_p^b$. We collect these matrices into a *block of matrices*

$$\underset{\sim}{C} \;=\; [C^1, \ldots, C^B].$$

Importantly, $\underset{\sim}{C}$ is not a tensor: while the number of rows is aligned across blocks (all blocks have n rows), the number of columns $\mid \mathrm{G}_p^b|$ can differ by cell type. Mathematically, C is best viewed as a tuple in the product space

$$\underset{\sim}{C} \in \prod_{b=1}^{B} \mathbb{R}^{n \times |G_p^b|}$$

Human sample labels. Let $Y_i \in \{0, 1\}$ denote the class label for human sample i (target vs. base), and

$$Y = (Y_1, \ldots, Y_n)^\top \in \{0, 1\}^n.$$

We also define $n_1 = |\{i : Y_i = 1\}|$ and $n_0 = |\{i : Y_i = 0\}|$, with $n_0 + n_1 = n$.

Disease model $\log_2$ fold-changes. For each cell type b, the disease model $\log_2$ fold-change vector is

$$R^b \;=\; \{r_{\bar{g}}^b\}_{\bar{g} \in \bar{G}_p^b} \in \mathbb{R}^{|\bar{G}_p^b|}$$

We collect them into a *block of vectors*

$$\underset{\sim}{R} \;=\; [R^1, \ldots, R^B]$$

Analogously to C, R is *not* a matrix in general because the lengths $|\bar{G}_p^b|$ can differ across cell types. We therefore view

$$\underset{\sim}{R} \in \prod_{b=1}^{B} \mathbb{R}^{|\bar{G}_p^b|}$$

singIST-treated human data. Applying the disease model shifts to the base-class human data produces treated pseudobulk values $x_{ig}^{b'}$ and treated matrices

$$C^{b'} \in \mathbb{R}^{n \times |G_p^b|}, \qquad \underset{\sim}{C}' = [C^{1'}, \ldots, C^{B'}] \in \prod_{b=1}^{B} \mathbb{R}^{n \times |G_p^b|}$$

Thus, singIST treatment does not change dimensions; it only modifies entries where a cell type is mapped and a one-to-one ortholog exists.

asmPLS-DA scores and decompositions. Fitting asmPLS-DA on the human reference data yields a continuous predicted response (superpathway score)

$$\hat{y} = (\hat{y}_1, \ldots, \hat{y}_n)^\top \in \mathbb{R}^n$$

The score admits an additive decomposition by cell type:

$$\hat{y}_i = \sum_{b=1}^{B} \hat{\gamma}_i^b,$$

where for each cell type b,

$$\hat{\gamma}^b = (\hat{\gamma}_1^b, \ldots, \hat{\gamma}_n^b)^\top \in \mathbb{R}^n$$

Within each cell type, we further decompose by gene:

$$\hat{\gamma}_i^b = \sum_{g \in G_p^b} \hat{\delta}_{ig}^b, \qquad \hat{\delta}^b = \{\hat{\delta}_{ig}^b\}_{1 \le i \le n, g \in G_p^b} \in \mathbb{R}^{n \times |G_p^b|}$$

For treated data, the corresponding quantities $\hat{y}'$, $\hat{\gamma}^{b'}$, and $\hat{\delta}^{b'}$ have the same dimensions:

$$\hat{y}' \in \mathbb{R}^n, \quad \hat{\gamma}^{b'} \in \mathbb{R}^n, \quad \hat{\delta}^{b'} \in \mathbb{R}^{n \times |G_p^b|}$$

Reference and predicted recapitulation measures. The superpathway *reference shift* is a scalar

$$\hat{\Omega} \in \mathbb{R},$$

computed as a difference of medians between target and base classes. The cell-type-specific *reference shifts* are also scalars:

$$\hat{\Gamma}^b \in \mathbb{R}, \qquad b = 1, \ldots, B.$$

The singIST *predicted shifts* (computed within the base class by comparing treated vs. untreated) are scalars:

$$\hat{\Omega}' \in \mathbb{R}, \qquad \hat{\Gamma}^{b'} \in \mathbb{R}.$$

The reported *recapitulations* are scalar percentages:

$$\hat{\Omega} f \in \mathbb{R}, \qquad \hat{\Gamma} f^b \in \mathbb{R}.$$

Finally, gene-level attribution uses, for each b and $g \in G_p^b$, scalar increments and fractions:

$$\hat{\Delta}_g^b \in \mathbb{R}, \qquad \hat{\Delta} f_g^b \in \mathbb{R}.$$

Block terminology. When we refer to a "block of matrices" (e.g., $\underset{\sim}{C}$) or a "block of vectors" (e.g., $\underset{\sim}{R}$), we do not mean a tensor or a single concatenated matrix. Rather, we follow the multi-block methodology convention, where a block variable is a tuple of datasets aligned on samples (rows) but potentially differing in feature dimension (columns), which in our setting arises because $|G_p^b|$ and $|\bar{G}_p^b|$ may vary with the cell type b.

B. Introduction to asmbPLS-DA

Here we introduce adaptive sparse multiblock partial least square discriminant analysis (asmbPLS-DA) (Zhang and Datta, 2023), a multiblock data fusion method that accounts for sparsity criteria adaptive to each predictor block. asmbPLS-DA is based on sparse multiblock partial least squares discriminant analysis (smbPLS-DA) (Li et al., 2012).

Let $\boldsymbol{X} = [\boldsymbol{X}^1, \ldots, \boldsymbol{X}^B]$ and $\boldsymbol{Y}$ be the predictor matrix and outcome matrix, respectively, that are defined on the same samples n. Concretely, the samples are split into G groups $n_1 + n_2 + \cdots + n_G = n$The outcome matrix $\boldsymbol{Y}$ is one-hot-encoded (1/0), with only one column for the binary outcome ($G = 2$) and G columns for the multiclasss outcome ($G \geq 3$).

The objective of asmbPLS-DA is to build a set of orthogonal PLS components such that the covariance between the latent scores t_j^{super} and u_j, which represent information from $\boldsymbol{X}$ and $\boldsymbol{Y}$ respectively, is maximized for each PLS component $j = 1, 2, \ldots, J$. Formally, the problem can be stated as an optimization problem.

$$\max_{\{\omega_j^{super}, \omega_j^b, q_j\}} Cov(\boldsymbol{t}_j^{super}, \boldsymbol{u}_j) \tag{S1}$$

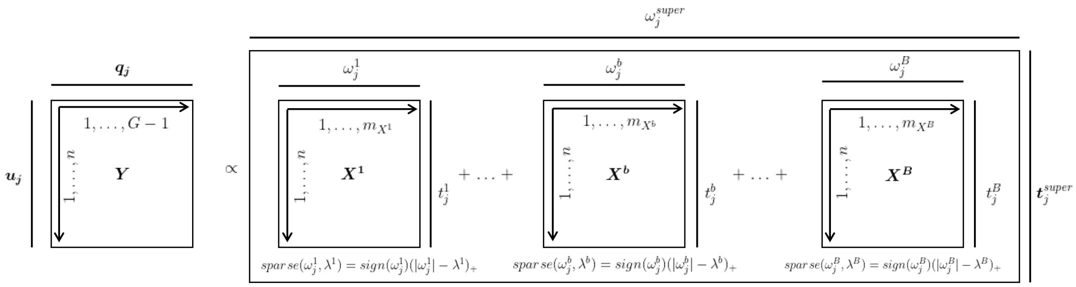

Fig. S1. Representation of asmbPLS-DA.

Source: own elaboration.

$$\text{subject to} \quad ||\omega_j^b|| = ||\omega_j^{super}|| = 1$$

$$\text{where} \quad t_j^b = \frac{\boldsymbol{X}^b \omega_j^b}{\sqrt{m_{X^b}}}, \quad \boldsymbol{T}_j = [t_j^1, \ldots, t_j^B], \quad \boldsymbol{u_j} = \boldsymbol{Y}\boldsymbol{q_j}, \quad \boldsymbol{t}_j^{super} = \boldsymbol{T}_j \omega_j^{super}$$

The optimization algorithm to solve (1) revolves around the following steps for each PLS component $j = 1, \ldots, J$.

- The first dimension reduction is conducted by taking a linear combination of the predictor features (columns) to obtain each of the block scores $t_j^b = \frac{\boldsymbol{X}^b \omega_j^b}{\sqrt{m_{X^b}}}$ for each of the predictor blocks $b = 1, \ldots, B$. Where ω_j^b contains the block feature weight in $\boldsymbol{X}^b$ which indicates the relevance of the feature to classify between groups, and m_{X^b} is the number of variables in X^b for block scaling.

- Within each block, the soft tresholding function $sparse(\omega_j^b, \lambda^b) = sign(\omega_j^b)(|\omega_j^b| - \lambda^b)$ is implemented as a sparsity criteria which sets as 0 all weights below a quantile treshold $\lambda^b \in [0, 1]$ selected by Cross Validation. By doing this, only the most relevant features will be retained.

- All block scores are combined into $\boldsymbol{T}_j = [t_j^1, \ldots, t_j^B]$ and a new dimension reduction is implemented again by taking a linear combination of the different block scores $\boldsymbol{t}_j^{super} = \boldsymbol{T}_j \omega_j^{super}$, where ω_j^{super} is the relevance of each block of the matrix $\boldsymbol{X}$ in classifying, while $\boldsymbol{t}_j^{super}$ is the super score that represents the information contained in predictor $\boldsymbol{X}$.

- Finally, the summary vector $\boldsymbol{u}_j$ contains the information on the response $\boldsymbol{Y}$ and q_j is the weight of each column of $\boldsymbol{Y}$, hence $\boldsymbol{u}_j = \boldsymbol{Y} q_j$ is computed.

Once all parameters are estimated $\{\omega_j^{super}, \omega_j^b, q_j\}$ for the first PLS component, $\boldsymbol{X}$ and $\boldsymbol{Y}$ are deflated, and then the deflated matrices are used again for the calculation of the second PLS component, and so on. With that a prediction of $\boldsymbol{Y}$ for a new set of predictors $\boldsymbol{X'}$ are computed through a linear combination.

$$\boldsymbol{Y'} = \boldsymbol{t}_1^{super'} q_1 + \boldsymbol{t}_2^{super'} q_2 + \cdots + \boldsymbol{t}_J^{super'} q_J \tag{S2}$$

With the estimated numeric prediction $\boldsymbol{Y'}$ different decision rules to discriminate between classes, and assign one of the classes $1, 2, \ldots, G$, are implemented in asmbPLS-DA from: fixed cut-off, euclidean distance, mahalanobis distance, principal component analysis and mahalanobis distance, etc.

Note that many solutions can arise from (1) based on the selection of the number of PLS components J and the quantile treshold for each block $\lambda = [\lambda^1, \ldots, \lambda^B]$, to select the optimal value for the hyperparameters a Cross Validation with K-fold is implemented in asmbPLS-DA. The K-fold CV selects the best hyperparameters for which the highest balanced accuracy (BA) is

attained. The samples are randomly placed into K groups with the ratios of the samples from different classes being equal in all the groups. Such step is repeated N_{CV} times to generate N_{CV} set of groups. For each of the K groups the last dataset is used for validation while the rest $K-1$ are used for training. The average K-fold BA is computed as an average over the N_{CV} sets:

$$BA_{N_{CV},K} = \frac{1}{N_{CV}} \sum_{n_{CV}=1}^{N_{CV}} \frac{1}{K} \sum_{k=1}^{K} BA_{n_{CV},k} \tag{S3}$$

Where $BA_{n_{CV},k}$ is the balanced accuracy using samples from the $k-th$ group of $n_{CV}-th$ set as the validation, the model with the lowest (3) is chosen. The optimal number J^* of PLS components are determined by initially selecting $J^*=1$, then check whether including one more component decreases the BA by a treshold $0<\varepsilon<0.005$, i.e $BA_{J^*+1}+\varepsilon \leq BA_{J^*}$, if true then set J^* to J^*+1 until the treshold criteria is not attained.

C. Gene orthology mapping

Gene orthology mapping between humans and disease models were retrieved from the ENSEMBL (Yates et al., 2019) homology between *Homo sapiens* and *Mus musculus*. Only one-to-one orthologs were retrieved, excluding many-to-many and one-to-many relationships. Entrez gene symbols were mapped to HGNC using biomaRt v2.58.2 (Durinck et al., 2009), which were posteriorely used for singIST workflow.

D. asmbPLS-DA parameter tuning and model selection

The optimal model is fitted using Leave-One-Out Cross Validation (LOOCV), due to the small sample size of scRNA-seq data, to select the number of PLS components J^*, as well as the quantile combination for each block and PLS component $\lambda_j^b = \text{quantile}\{|\omega_j^b|, \lambda^b\}$, λ^b is the hyperparameter for quantile tuning $\lambda^b \in [0,1]$, that is used for the sparse criteria $sparse(\omega_j^b, \lambda_j^b) = sign(\omega_j^b)(|\omega_j^b| - \lambda_j^b)_+$. The LOOCV method aims to identify the most effective quantile sparsity combination, for each PLS component j, that yield the highest F_1 score.

E. Computation of log2FC of disease model

The objective of this section will be to detail the workflow to obtain the information for Eq (1) of manuscript:

$$r_{\tilde{g}}^b := \begin{cases} 0 & \mathrm{p}_{\tilde{g}}^b > 0.05 \\ \log_2 FC_{\tilde{g}}^b & \mathrm{p}_{\tilde{g}}^b \leq 0.05 \end{cases} \tag{S4}$$

Which are the $\log_2 FC$ of the disease model used by singIST. We detail here the workflow as used by R/singIST library. For a fixed superpathway $\mathcal{P}^p$ and disease model:

1. Determine statistically significant genes ($p_{\tilde{g}}^b$ adjusted p-value) within $\mathcal{P}^p$ with `Seurat::FindMarkers`.

2. Compute descriptive point estimate of $\log_2 FC$:

   (a) Pseudobulk and log-normalize the scRNA-seq object and extract the layer data.

   (b) A descriptive point estimate of $\log_2 FC$ is given by: $\log_2 FC_{\tilde{g}}^b = \overline{x}_{\tilde{g}}^b - \overline{y}_{\tilde{g}}^b$, where $\overline{x}_{\tilde{g}}^b$ and $\overline{y}_{\tilde{g}}^b$ are the mean of the log-normalized expression values for samples belonging to the target and base class, respectively.

3. Compute $r_{\tilde{g}}^b$:

   (a) If $p_{\tilde{g}}^b \leq 0.05$ then $r_{\tilde{g}}^b = \log_2 FC_{\tilde{g}}^b$ else $r_{\tilde{g}}^b = 0$.

F. Superpathway's score $\widehat{y}_i$ decomposition onto cell type $\widehat{\gamma}_i^b$ and gene $\widehat{\delta}_{ig}^b$ contributions

For a generic sample i and number of PLS J, the asmbPLS-DA predictor is $\widehat{y}_i = \sum_{j=1}^{J} t_{ij}^{super} q_j^T$. Denote $\boldsymbol{T} = [t_{ij}^1, \ldots, t_{ij}^b, \ldots, t_{ij}^B]$ then $\boldsymbol{t}_{ij}^{super} = (\boldsymbol{T} \times_1 \omega^{super}) = \sum_{b=1}^{B} t_{ij}^b \left(\omega_{bj}^{super}\right)^T$, where $(\boldsymbol{T} \times_1 \omega^{super})$ denotes the n-mode product over the mode 1. Hence plugging that in the predictor:

$$\widehat{y}_i = \sum_{j=1}^{J} t_{ij}^{super} q_j^T = \sum_{j=1}^{J} \left[\sum_{b=1}^{B} t_{ij}^b \left(\omega_{bj}^{super}\right)^T\right] q_j^T = \sum_{j=1}^{J} \left[\sum_{b=1}^{B} t_{ij}^b \left(\omega_{bj}^{super}\right)^T q_j^T\right] \tag{S5}$$

$$= \sum_{b=1}^{B} \left[\sum_{j=1}^{J} t_{ij}^b \left(\omega_{bj}^{super}\right)^T q_j^T\right] = \sum_{b=1}^{B} \widehat{\gamma}_i^b \tag{S6}$$

Where $\widehat{\gamma}_i^b = \sum_{j=1}^{J} t_{ij}^b \left(\omega_{bj}^{super}\right)^T q_j^T$ is the cell type contribution to $\widehat{y}_i$. To obtain the gene contributions note that the gene score $t_{ij}^b = \frac{\boldsymbol{C}^b \omega^b}{\sqrt{|\mathcal{G}_p^b|}} = \sum_{g \in \mathcal{G}_p^b} \frac{x_{ig}^b \omega_{gj}^b}{\sqrt{|\mathcal{G}_p^b|}}$, and plugging such into Eq. (S6):

$$\widehat{y}_i = \sum_{b=1}^{B} \left[\sum_{j=1}^{J} t_{ij}^b \left(\omega_{bj}^{super}\right)^T q_j^T\right] = \sum_{b=1}^{B} \left[\sum_{j=1}^{J} \left(\sum_{g \in \mathcal{G}_p^b} \frac{x_{ig}^b \omega_{gj}^b}{\sqrt{|\mathcal{G}_p^b|}}\right) \left(\omega_{bj}^{super}\right)^T q_j^T\right] \tag{S7}$$

$$= \sum_{b=1}^{B} \left[\sum_{j=1}^{J} \left(\sum_{g \in \mathcal{G}_p^b} \frac{x_{ig}^b \omega_{gj}^b}{\sqrt{|\mathcal{G}_p^b|}} \left(\omega_{bj}^{super}\right)^T q_j^T\right)\right] = \sum_{b=1}^{B} \sum_{g \in \mathcal{G}_p^b} \left[\sum_{j=1}^{J} \frac{x_{ig}^b \omega_{gj}^b}{\sqrt{|\mathcal{G}_p^b|}} \left(\omega_{bj}^{super}\right)^T q_j^T\right] \tag{S8}$$

Which gets us to $\widehat{y}_i = \sum_{b=1}^{B} \sum_{g \in \mathcal{G}_p^b} \widehat{\delta}_{ig}^b$, where $\widehat{\delta}_{ig}^b = \sum_{j=1}^{J} \frac{x_{ig}^b \omega_{gj}^b}{\sqrt{|\mathcal{G}_p^b|}} \left(\omega_{bj}^{super}\right)^T q_j^T$ is the gene contribution to $\widehat{y}_i$. To compute predictions the blocks are loading deflated.

G. Cell type recapitulation as sum of gene contributions $\widehat{\Gamma} f^b = \sum_g \widehat{\Delta} f_g^b$

Since $\ell(x_{ig}^b, r_{\tilde{g}}^b)$ defines an affine transformation, the change $x_{ig}^{b\,\prime} - x_{ig}^b$ is constant for all original samples $i \in K := \{1 \leq i \leq n | y_i = 1\}$, further the predictor $\widehat{y}_i$ is a linear transformation, thus the change $\widehat{y}_i^{\prime} - \widehat{y}_i$ is also constant for all original samples $i \in K$. As shown in Eq. (S6) the predictor $\widehat{y}_i$ can be decomposed onto cell contributions $\widehat{y}_i^{\prime} - \widehat{y}_i = \sum_{b=1}^{B} \left(\widehat{\gamma}_i^{b\prime} - \widehat{\gamma}_i^b\right)$, for which there $\exists \{\widetilde{\gamma}^b\}_b$ constants such that $\widehat{y}_i^{\prime} - \widehat{y}_i = \sum_{b=1}^{B} \widetilde{\gamma}^b$. Without loss of generality fix a $b \in \{1, \ldots, B\}$, the constant difference in cell contribution is $\widehat{\gamma}_i^{b\prime} = \widetilde{\gamma}^b + \widehat{\gamma}_i^b$, note that applying $\underset{i \in K}{\text{median}} \left(\widehat{\gamma}_i^{b\prime}\right) = \underset{i \in K}{\text{median}} \left(\widetilde{\gamma}^b + \widehat{\gamma}_i^b\right)$ since *singIST treated samples* do not belong to K samples $\widehat{\gamma}_i^{b\prime} = \widetilde{\gamma}^b + \underset{i \in K}{\text{median}} \left(\widehat{\gamma}_i^b\right)$, plugging it into the observed cell type recapitulation:

$$\widehat{\Gamma}^b = \underset{i \in K^c}{\text{median}} \left(\widehat{\gamma}_i^{b\prime}\right) - \underset{i \in K}{\text{median}} \left(\widehat{\gamma}_i^b\right) = \underset{i \in K^c}{\text{median}} \left(\widetilde{\gamma}^b + \underset{i \in K}{\text{median}} \left(\widehat{\gamma}_i^b\right)\right) - \underset{i \in K}{\text{median}} \left(\widehat{\gamma}_i^b\right) \tag{S9}$$

$$= \widetilde{\gamma}^b \tag{S10}$$

With the same reasoning, there also $\exists \{\widehat{\Delta}_g^b\}_{g,b}$ such that $\widetilde{\gamma}^b = \sum_{g \in \mathcal{G}_p^b} \widehat{\Delta}_g^b$, which is straightforward then that $\widehat{\Gamma} f^b = \sum_{g \in \mathcal{G}_p^b} \widehat{\Delta} f_g^b$.

H. Recapitulations as a function of ω^{super}, ω^b, $\exists b$, $\exists \tilde{g} \equiv g$, $r_{\tilde{g}}^b$

For a fixed b and g, we start by explicitly computing the absolute gene contribution to cell type recapitulation $\widehat{\Delta}_g^b := \widehat{\delta}_{ig}^{b\,\prime} - \widehat{\delta}_{ig}^b$:

$$\widehat{\Delta}_g^b = \sum_{j=1}^{J} \frac{q_j^T}{\sqrt{|\mathcal{G}_p^b|}} \left(\omega_{bj}^{super}\right)^T \omega_{gj}^b \left(x_{ig}^{b\,\prime} - x_{ig}^b\right) \tag{S11}$$

If $\nexists b$ and/or $\nexists \tilde{g} \equiv g$ by construction $x_{ig}^{b}{}' = x_{ig}^{b}$, hence $\widehat{\Delta}_{g}^{b} = 0$ which yields to $\widehat{\Delta} f_{g}^{b} = 0$. Similarly, assume $GIP_{g}^{b} = 0$ which implies that $\omega_{gj}^{b} = 0 \quad \forall j$, then $\widehat{\Delta}_{g}^{b} = 0$ indistinguisably of $x_{ig}^{b}{}' - x_{ig}^{b}$. Likewise, if a cell type is not important in human condition $CIP^{b} = 0$ it implies that $\omega_{bj}^{super} = 0 \quad \forall j$, hence $\widehat{\Gamma}^{b} = \sum_{g \in \mathcal{G}_{p}^{b}} \widehat{\Delta}_{g}^{b} = 0$ and the cell type recapitulation becomes null $\widehat{\Gamma} f^{b} = 0$.

Assume a non trivial case where $CIP^{b}, GIP_{g}^{b} > 0$ and conditions $\exists b$, $\exists \tilde{g} \equiv g$ hold true, then Eq. (S12) becomes:

$$\widehat{\Delta}_{g}^{b} = \frac{r_{\tilde{g}}^{b}}{\sqrt{|\mathcal{G}_{p}^{b}|}} \sum_{j=1}^{J} q_{j}^{T} \left(\omega_{bj}^{super} \right)^{T} \omega_{gj}^{b} \tag{S12}$$

Note that $|\mathcal{G}_{p}^{b}|$ is always positive and so are q_{j}^{T} and $\left(\omega_{bj}^{super} \right)^{T}$ when class is one-hot-encoded, hence the direction of $\widehat{\Delta}_{g}^{b}$ as $sign\left(\widehat{\Delta}_{g}^{b}\right) = sign\left(\sum_{j=1}^{J} q_{j}^{T} \left(\omega_{bj}^{super}\right)^{T} \omega_{gj}^{b}\right) sign\left(r_{\tilde{g}}^{b}\right)$, as the agreement in direction between the disease model estimated FC and the estimated direction of the gene within the cell type for humans. Note that differences between disease models' recapitulation will be based on their orthology, cell type agreement and estimated FC, as all other parameters are fixed by disease model.

I. Parameters variabilities and significance of the optimal asmbPLS-DA

First, we prove that $\sum_{g \in \mathcal{G}_{p}^{b}} GIP_{g}^{b} = 1$. From the optimization problem of asmbPLS-DA it stems that $||\omega_{\cdot j}^{b}|| = 1$.

$$\sum_{g \in \mathcal{G}_{p}^{b}} GIP_{g}^{b} = \frac{\sum_{g \in \mathcal{G}_{p}^{b}} \sum_{j=1}^{J^{*}} q_{j} \left(\omega_{gj}^{b}\right)^{2}}{\sum_{j=1}^{J^{*}} q_{j}} = \frac{\sum_{j=1}^{J^{*}} \left[q_{j} \sum_{g \in \mathcal{G}_{p}^{b}} \left(\omega_{gj}^{b}\right)^{2} \right]}{\sum_{j=1}^{J^{*}} q_{j}} = \frac{\sum_{j=1}^{J^{*}} q_{j} ||\omega_{\cdot j}^{b}||^{2}}{\sum_{j=1}^{J^{*}} q_{j}} = 1$$

We now detail the rationale of Bonferroni's multiple testing correction of GIP_{g}^{b} significance test by using $m_{0}^{b} = \left\lfloor \prod_{j=1}^{J^{*}} \lambda_{j}^{b} |\mathcal{G}_{p}^{b}| \right\rfloor$ as the minimum number of true hypothesis. For cell type b, let $H_{0,1}^{b}, \ldots, H_{0,g}^{b}, \ldots, H_{0,g_{b}}^{b}$ be the family of null hypothesis defined as $H_{0,g}^{b}$: median$(\mathcal{D}_{g,Jackknife}^{b}) \leq$ median$(\mathcal{D}_{0,g}^{b})$ and their corresponding p-values $p_{1}^{b}, \ldots, p_{g}^{b}, \ldots, p_{g_{b}}^{b}$. $|\mathcal{G}_{p}^{b}|$ are the total number of hypothesis to be tested and M_{0}^{b} the number of true null hypothesis. We provide a lower bound of M_{0}^{b} by noting that for any cell with quantile sparsity values $\{\lambda_{j}^{b}\}_{j=1}^{J^{*}}$ it holds that $P_{\mathcal{D}}(GIP_{g}^{b} = 0) = \prod_{j=1}^{J^{*}} \lambda_{j}^{b}$ for any distribution $\mathcal{D}$, and non-trivial gene $x_{ig}^{b} = 0, \forall i$. asmbPLS-DA retains the top $\lambda_{j}^{b} \in [0,1]$ weights, hence by definition of the sparsity criteria and orthogonality of PLS components $P_{\mathcal{D}}(\{\omega_{gj}^{b} = 0 : \forall j\}) = \prod_{j=1}^{J^{*}} \lambda_{j}^{b}$ for any non-trivial gene g. Without loss of generality assume the weights $q_{j} \geq 0$, which is always the case when the class is one-hot-encoded and/or response scale is greater than 0.

$$P_{\mathcal{D}}\left(GIP_{g}^{b} = 0 | q_{j} \geq 0, \forall j\right) = P_{\mathcal{D}}(\{\omega_{gj}^{b} = 0 : \forall j\}) = \prod_{j=1}^{J^{*}} \lambda_{j}^{b}, \quad \forall \mathcal{D}$$

In conclusion, for at least $\left\lfloor \prod_{j=1}^{J^{*}} \lambda_{j}^{b} |\mathcal{G}_{p}^{b}| \right\rfloor$ tests the null hypothesis will be true due to the sparsity criteria. The lower bound of the number of true null hypothesis $M_{0}^{b} \geq m_{0}^{b} = \left\lfloor \prod_{j=1}^{J^{*}} \lambda_{j}^{b} |\mathcal{G}_{p}^{b}| \right\rfloor$ serves as a correction value to preserve a confidence treshold of α, if $\left\lfloor \prod_{j=1}^{J^{*}} \lambda_{j}^{b} \right\rfloor = 0$ then m_{0}^{b} was set to $m_{0}^{b} = 1$

$$FWER_{Bonferroni} \leq \frac{m_{0}^{b}}{|\mathcal{G}_{p}^{b}|} \alpha \leq \alpha$$

Which trivially holds true due to the chain of inequalities:

$$\alpha \geq \frac{M_0^b}{|\mathcal{G}_p^b|}\alpha \geq \frac{m_0^b}{|\mathcal{G}_p^b|}\alpha \geq \frac{\prod_{j=1}^{J^*} \lambda_j^b |\mathcal{G}_p^b|}{|\mathcal{G}_p^b|}\alpha = \prod_{j=1}^{J^*} \lambda_j^b \alpha$$

REFERENCES

Brunner, Peter M et al. (2017). "The atopic dermatitis blood signature is characterized by increases in inflammatory and cardiovascular risk proteins". In: *Scientific reports* 7.1, p. 8707.

Durinck, Steffen et al. (2009). "Mapping identifiers for the integration of genomic datasets with the R/Bioconductor package biomaRt". In: *Nature Protocols* 4.8, pp. 1184–1191.

Gillespie, M. et al. (2022). "The Reactome Pathway Knowledgebase 2022". In: *Nucleic Acids Research* 50.D1, pp. D687–D692.

Kanehisa, Minoru et al. (2023). "KEGG for taxonomy-based analysis of pathways and genomes". In: *Nucleic acids research* 51.D1, pp. D587–D592.

Li, Wenyuan et al. (Aug. 2012). "Identifying multi-layer gene regulatory modules from multi-dimensional genomic data". In: *Bioinformatics* 28.19, pp. 2458–2466. ISSN: 1367-4803.

Liberzon, Arthur et al. (2015). "The Molecular Signatures Database (MSigDB) hallmark gene set collection". In: *Cell systems* 1.6, pp. 417–425.

Nishimura, Darryl (2001). "BioCarta". In: *Biotech Software & Internet Report* 2.3, pp. 117–120.

Schaefer, Carl F. et al. (Oct. 2008). "PID: the Pathway Interaction Database". In: *Nucleic Acids Research* 37.suppl$_1$, pp. D674–D679. ISSN: 0305-1048.

Yates, Andrew D et al. (Nov. 2019). "Ensembl 2020". In: *Nucleic Acids Research* 48.D1, pp. D682–D688. ISSN: 0305-1048.

Zhang, R and S Datta (2023). "Adaptive Sparse Multi-Block PLS Discriminant Analysis: An Integrative Method for Identifying Key Biomarkers from Multi-Omics Data". In: *Genes (Basel)* 14.5, p. 961.
