# Supplementary material for: singIST: An integrative method for comparative single-cell transcriptomics between disease models and humans
Source: PLoS Comput Biol. 2026 Mar 16;22(3):e1014002. doi: 10.1371/journal.pcbi.1014002 (PMC13008255; doi:10.1371/journal.pcbi.1014002)
Supplement: S2 File — Metadata for all human and mouse samples, including demographics, experimental conditions, sequencing protocol, and GEO accession numbers. (PDF) [file pcbi.1014002.s002.pdf]

Organism	Cluster	Cell type granularity 1**	Cell type granularity 2***	Action	Reason
	KC-1		Suprabasal Keratinocyte		No relevant information reported on granularity 2 Keratinocytes in the original paper, only for granularity 1 Keratinocyte
	KC-2		Basal Keratinocyte		
	KC-3	Keratinocytes	Late differentiation Keratinocyte	KEEP GRANULARITY 1	
	KC-4		Proliferating Keratinocyte		
	KC-5		?		
	Tregs	T-cell	T-regs		
	TC-1	T-cell	Tissue Resident Memory T-cell	KEEP GRANULARITY 2	
	TC-2	T-cell	CD8+ effector T-cell		
	TC-3	T-cell	(CD161)+ T-cell / Th2a ?	DROP CLUSTER	- Small cluster according to [1] "*The smaller clusters TC-3, TC-4, and TC-5 were either absent (TC-3) or only detectable in small numbers (TC-4 and TC-5) in healthy control samples* " - Not clear from the publication if its (CD161)+ T-cell or Th2A
	TC-4	T-cell	?	DROP CLUSTER	Non-identified cell type
	TC-5	T-cell	Proliferating T-cell	DROP CLUSTER	Small cluster according to [1] "*The smaller clusters TC-3, TC-4, and TC-5 were either absent (TC-3) or only detectable in small numbers (TC-4 and TC-5) in healthy control samples* "
*Homo Sapiens Sapiens**	TC-6	T-cell	Natural Killer T-cell	KEEP GRANULARITY 2	
	Melanocytes	Melanocytes		KEEP GRANULARITY 1	It's the only granularity reported
	LC	Dendritic cells	Langerhans cells	KEEP GRANULARITY 2	
	DC-1	Dendritic cells	Myeloid cells		
	DC-2	Dendritic cells	Mature DCs	DROP CLUSTER	- Not relevant according to [1] "*Although the list of differentially expressed genes in DC-2 was relatively short, likely due to very small cell numbers and thus lacking statistical power, a few anti-inflammatory genes showed marked up-regulation during treatment, especially,...* " - We are not considering treatment period, only HC and Baseline. - Very small cluster to consider it for analysis
	DC-3	Dendritic cells	Plasmacytoid DCs	DROP CLUSTER	- Very small population according to [1] "*We found a very small population of plasmacytoid DCs (DC-3)...* "
	MastC_Others	?		DROP CLUSTER	Non-identified cell type

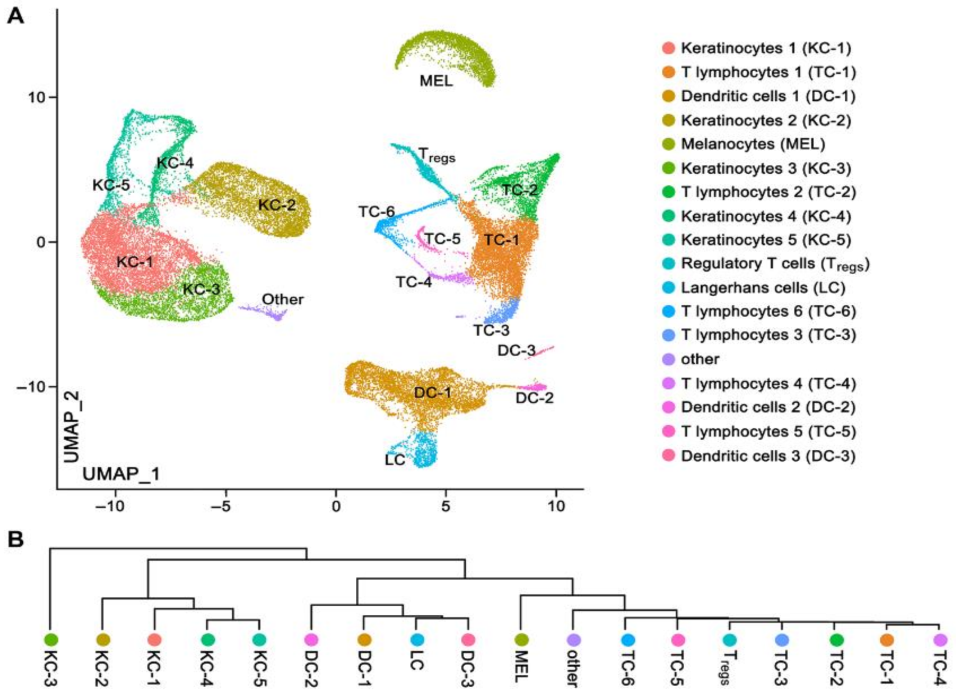

*Human data extracted from [1] "*Persistence of mature dendritic cells, TH2A, and Tc2 cells characterize clinically resolved atopic dermatitis under IL-4Ralpha blockade* " url:
** If "?" cell type granularity 1 is not clearly identified in the publication
*** If "?" cell type granularity 2 is not clearly identified in the publication

https://www.science.org/doi/epdf/10.1126/sciimmunol.abe2749

Organism	Disease model(s)	Cluster	Cell type granularity 1*	Cell type granularity 2*	Action	Reason
Mus musculus	*Imiquimod (5%)* and *Oxazolona 3 Challenges*	0	Mac (Macrophages)			
		1	DETC (Dendritic Epidermal T-cells)			
		2	M/MdM (Monocyte derived Macrophages)			
		3	dgammadeltaT			
		4	cDC2 (Type 2 conventional Dendritic cell)			
		5	ILC2 (Type 2 Innate Lymphoid cell)			
		6	Thet (T-cell heterogeneous)	Treg<br>Tconv (Conventional T-cell)<br>CD8+ (CD8+ effector T-cell)<br>DNT (Double Negative T-cell)		
		7	Fibroblasts1			
		8	Indeterminate1			
		9	M/B (Mast cells and Basophils)			
		10	Neu (Neutrophils)			
		11	Cell cycle1			
		12	Keratinocytes			
		13	Cell cycle2			
		14	cDC1 (Type 1 conventional Dendritic cell)			
		15	LC (Langerhans cell)			
		16	mDCs (Mature Dendritic cell)			
		17	NK (Natural Killer cell)			
		18	Endothelial			
		19	Indeterminate2			
		20	Fibroblasts2			

*IMQ-OXA data extracted from [2] "Single-Cell Profiling Reveals Divergent, Globally Patterned Immune Responses in Murine Skin Inflammation" url: https://pubmed.ncbi.nlm.nih.gov/33205009/

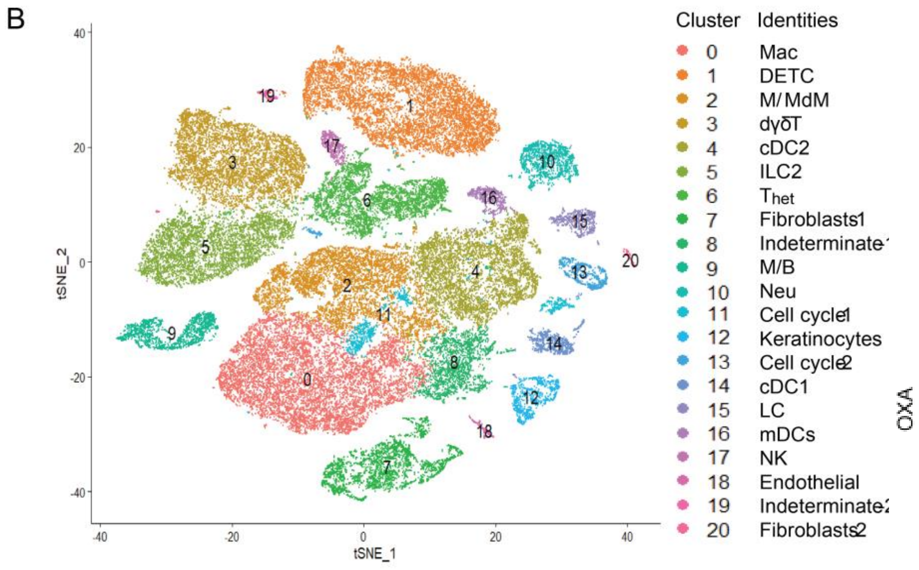

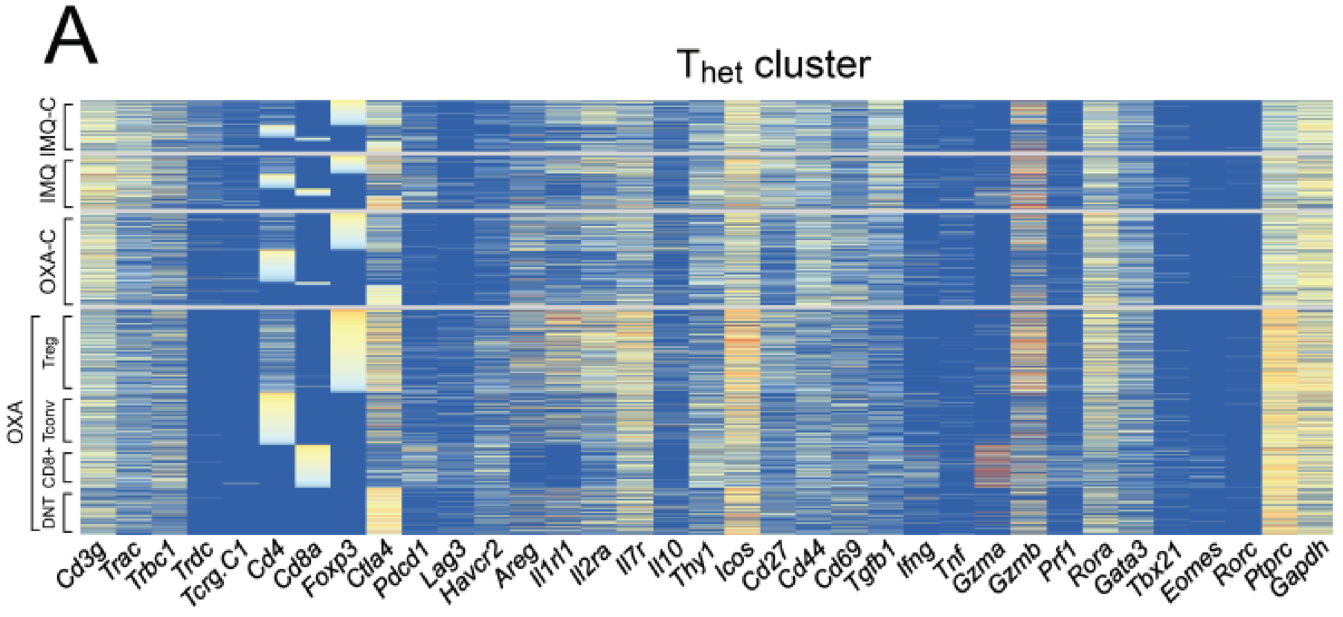

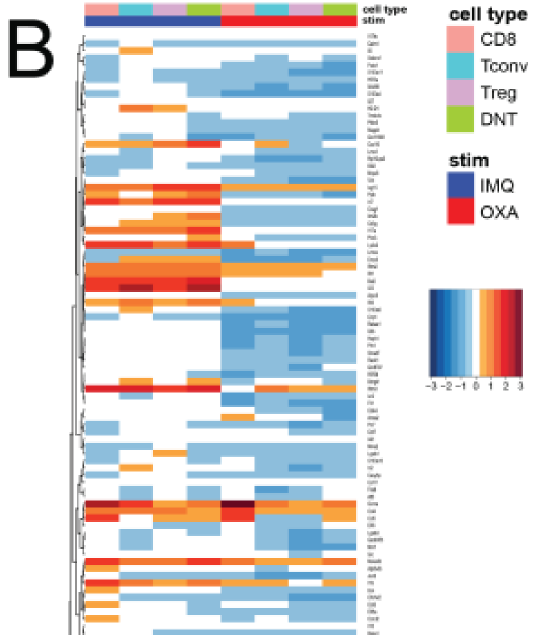

**Human cells**	**IMQ-OXA cluster**	**IMQ-OXA cells**	**Reason**
Keratinocytes	12	Keratinocytes	
Tregs	6	T-heterogeneous (Treg)	
Tissue Resident Memory T-cell	**?**	**?**	
CD8+ effector T-cell	6	T-heterogeneous (CD8+ effector T-cell)	
Natural Killer T-cell	17	NK	
Melanocytes		**None**	Melanocytes are not present in murine ear skin but either bulge and bulb regions of hair follicles, tail or ventral paws of non-hairy skin according to [3] "*Firstly, mouse pelage skin interfollicular epidermis entirely lacks functional, pigment-producing melanocytes. While murine melanocytes are found either in the bulge and bulb regions of hair follicles, in the tail, or in the ventral paws of non-hairy mouse skin, functional human melanocytes are mostly located in the basal layer of the epidermis (Gola et al., 2012).* "
Langerhans cells	15	LC (Langerhans cells)	
Myeloid cells	0, 2, 9, 10, 17	Mac (Macrophages), M/MdM (Monocyte derived Macrophages), M/B (Mast cells/Basophils), Neu (Neutrophils)	

[3] "*Characterization of a melanocyte progenitor population in human interfollicular epidermis* ". Cell Reports.

REFERENCES

1. Bangert C, Rindler K, Krausgruber T, Alkon N, Thaler FM, Kurz H, Ayub T, Demirtas D, Fortelny N,
Vorstandlechner V, Bauer WM, Quint T, Mildner M, Jonak C, Elbe-Bürger A, Griss J, Bock C,
Brunner PM. Persistence of mature dendritic cells, TH2A, and Tc2 cells characterize clinically resolved atopic dermatitis under IL-4Rα blockade.
Sci Immunol. 2021 Jan 22;6(55):eabe2749. doi: 10.1126/sciimmunol.abe2749. PMID: 33483337.
2. Theocharidis G, Tekkela S, Veves A, McGrath JA, Onoufriadis A.
Single-cell transcriptomics in human skin research: available technologies,
technical considerations and disease applications. Exp Dermatol. 2022; 31: 655–673. doi:10.1111/exd.14547
3. Michalak-Mićka K, Büchler VL, Zapiórkowska-Blumer N,
Biedermann T, Klar AS. Characterization of a melanocyte
progenitor population in human interfollicular epidermis.
Cell Rep. 2022 Mar 1;38(9):110419. doi: 10.1016/j.celrep.2022.110419. PMID: 35235792.

4. Yale Liu, Christopher Cook, Andrew J. Sedgewick, Shuyi Zhang, Marlys S. Fassett, Roberto R. Ricardo-Gonzalez, Paymann Harirchian, Sakeen W. Kashem, Sho Hanakawa, Jacob R. Leistico, Jeffrey P. North, Mark A. Taylor, Wei Zhang, Mao-Qiang Man, Alexandra Charruyer, Nadejda Beliakova-Bethell, Stephen C. Benz, Ruby Ghadially, Theodora M. Mauro, Daniel H. Kaplan, Kenji Kabashima, Jaehyuk Choi, Jun S. Song, Raymond J. Cho, Jeffrey B. Cheng,
Single-Cell Profiling Reveals Divergent, Globally Patterned Immune Responses in Murine Skin Inflammation,
iScience,
Volume 23, Issue 10,
2020,
101582,
ISSN 2589-0042,
https://doi.org/10.1016/j.isci.2020.101582.
(https://www.sciencedirect.com/science/article/pii/S2589004220307744)
