# Supplementary material for: singIST: An integrative method for comparative single-cell transcriptomics between disease models and humans
Source: PLoS Comput Biol. 2026 Mar 16;22(3):e1014002. doi: 10.1371/journal.pcbi.1014002 (PMC13008255; doi:10.1371/journal.pcbi.1014002)
Supplement: S3 File — Supplementary tables and figures of Results section. (PDF) [file pcbi.1014002.s003.pdf]

Supplementary Material S3: Results

1. ATOPIC DERMATITIS: EVALUATING MOUSE MODELS

Table A. Characteristics of fitted optimal asmbPLS-DA for all superpathways.

Pathway	J^{*a}	Gene set size[b]	adj pvalue[c]	Dendritic Cell[d]		Keratinocyte		Langerhans Cell		Melanocyte		T-cell	
				λ^1	GIP_g^1*	λ^2	GIP_g^2*	λ^3	GIP_g^3*	λ^4	GIP_g^4*	λ^5	GIP_g^5*
Cytokine-Cytokine receptor interaction [KEGG]	2	263	$p \leq 0.001$	0.75<br>0.05	31	0.95<br>0.05	18	0.55<br>0.05	50	0.95<br>0.65	28	0.95<br>0.45	17
Chemokine receptors bind chemokines [REACTOME]	1	57	$p \leq 0.001$	0.05	13	0.05	20	0.45	9	0.95	3	0.85	7
Chemokine signaling pathway [KEGG]	1	187	$p = 0.065$	0.05	35	0.05	44	0.05	37	0.05	41	0.05	39
Inflammation pathway [BIOCARTA]	1	27	$p \leq 0.05$	0.05	9	0.05	8	0.75	4	0.45	5	0.05	8
Th1/Th2 Differentiation [BIOCARTA]	1	21	$p = 0.065$	0.05	5	0.05	8	0.05	7	0.05	7	0.05	6
Cytokine Network [BIOCARTA]	1	19	$p \leq 0.05$	0.05	6	0.05	5	0.05	6	0.05	8	0.05	5
Dendritic Cells in Th1/Th2 Development [BIOCARTA]	1	17	$p \leq 0.05$	0.55	3	0.95	1	0.95	1	0.45	4	0.35	4
JAK-STAT signaling pathway [KEGG]	1	155	$p = 0.065$	0.05	34	0.05	38	0.05	32	0.05	41	0.05	32
Asthma [KEGG]	1	28	$p = 0.065$	0.05	8	0.05	11	0.95	2	0.95	2	0.05	8
Toll-like receptor signaling pathway [KEGG]	1	102	$p = 0.067$	0.05	21	0.05	30	0.05	23	0.05	24	0.05	22
IL12 signaling mediated by STAT4 [PID]	2	32	$p = 0.052$	0.05<br>0.85	6	0.05<br>0.85	7	0.05<br>0.95	5	0.05<br>0.95	7	0.05<br>0.45	10
CD40/CD40L signaling [PID]	1	15	$p = 0.067$	0.05	3	0.05	5	0.05	5	0.05	4	0.05	5
IL4-mediated signaling events [PID]	2	64	$p = 0.052$	0.05<br>0.45	19	0.05<br>0.85	13	0.05<br>0.85	14	0.05<br>0.95	16	0.05<br>0.95	11
IL23-mediated signaling events [PID]	2	37	$p = 0.052$	0.05<br>0.05	10	0.05<br>0.95	8	0.05<br>0.25	13	0.05<br>0.85	9	0.05<br>0.05	10
CXCR3-mediated signaling events [PID]	1	43	$p = 0.053$	0.05	9	0.25	12	0.05	8	0.95	3	0.55	9
Hematopoietic cell lineage [KEGG]	2	85	$p = 0.052$	0.05<br>0.25	23	0.05<br>0.95	21	0.35<br>0.95	15	0.05<br>0.95	15	0.35<br>0.05	26
IL2 signaling events mediated by STAT5 [PID]	1	30	$p = 0.052$	0.75	3	0.95	2	0.65	7	0.75	4	0.85	4
NOD-like receptor signaling pathway [KEGG]	1	62	$p \leq 0.05$	0.75	10	0.85	8	0.95	3	0.75	10	0.95	4
Downstream signaling in naïve CD8+ T cells [PID]	2	65	$p \leq 0.001$	0.05<br>0.55	20	0.05<br>0.95	18	0.05<br>0.95	18	0.45<br>0.85	11	0.95<br>0.05	20
T cell receptor signaling pathway [KEGG]	1	108	$p = 0.052$	0.05	20	0.05	27	0.05	23	0.15	20	0.25	20
Cytokine signaling in Immune system [REACTOME]	2	701	$p = 0.052$	0.05<br>0.05	199	0.05<br>0.05	232	0.05<br>0.85	119	0.05<br>0.05	221	0.05<br>0.05	200
Signaling by Interleukins [REACTOME]	1	449	$p = 0.052$	0.95	20	0.85	23	0.65	91	0.95	19	0.85	52

a. Optimal number of PLS components, for LOOCV was set to $J \leq 3$, *b*. Gene set size *c*. Adj. p-val of global significance, *d*. λ^b optimal quantiles of cell type *b*. The quantile space were set to 100000 combinations of λ^b values ranging along {0.05, ..., 0.55, ..., 0.95}. GIP_g^b* number of statistically significant GIP_g^b whose $adj.p - value \leq 0.05$, permutation tests were run on 10000 permutations.

Table B. Top five statistically significant genes ordered by GIP_g^b magnitude.

Pathway	Cell type	CIP (direction)	Top 5 genes[a]	GIP (direction)	Reference direction[b,c,d]
JAK-STAT signaling pathway [KEGG]	T-cell	0.17 (↑)	IL13, IL26, IL2RA, IL7, IFNGR1	0.05 (↑), 0.03 (↑), 0.03 (↑), 0.03 (↑), 0.03 (↑)	$\uparrow, \uparrow, \uparrow, -, -$
	Dendritic Cell	0.19 (↑)	IL23A, SPRED1,SOCS1, IFNL1, OSM	0.05 (↓), 0.04 (↑), 0.04 (↑), 0.03(↓), 0.03 (↑),	$\uparrow^{\dagger}, \uparrow^{*,\dagger}, \uparrow^{\dagger}, \downarrow^{\dagger}, \uparrow$
	Langerhans Cell	0.17 (↑)	IL22RA2, CCND2, CCND1, JAK1, STAT6	0.04 (↑), 0.04 (↓), 0.04 (↑), 0.04 (↑), 0.03 (↓)	$\uparrow, -, -, \uparrow^{\dagger}, -$
	Keratinocyte	0.28 (↑)	CCND3, SPRY1, IL15RA, IFNAR2, IL15	0.03 (↑), 0.03 (↓), 0.02 (↑), 0.02 (↑), 0.02 (↑)	$-, \downarrow^{*}, \uparrow^{*}, \uparrow^{*}, \uparrow$
	Melanocyte	0.18 (↑)	CCND3, IFNGR2, CCND2, IL10RA, CCND1	0.03 (↑), 0.03 (↑), 0.03 (↑), 0.03 (↑), 0.03(↑)	$\uparrow^{*}, \uparrow^{*}, -, \uparrow^{*}, \uparrow^{*}$
Dendritic Cells in Th1/Th2 development [BIOCARTA]	T-cell	0.38 (↑)	IL13, IL5, CSF2, TLR7	0.42 (↑), 0.13 (↑), 0.11 (↑), 0.11 (↓)	$\uparrow, \uparrow, \uparrow, \downarrow^{*}$
	Dendritic Cell	0.16 (↑)	ANPEP, CSF2, IL13	0.85 (↑), 0.06 (↑), 0.04 (↑)	$\uparrow^{\dagger}, \uparrow^{\dagger}, \uparrow$
	Langerhans Cell	0.11 (↑)	ANPEP	1 (↑)	$\uparrow^{\dagger}$
	Keratinocyte	0.09 (↑)	ITGAX	1 (↑)	$\uparrow^{\dagger}$
	Melanocyte	0.26 (↑)	IL10, ITGAX, CD7, CD33	0.39 (↑), 0.23 (↑), 0.14 (↑), 0.10 (↑)	$\uparrow^{\dagger}, \uparrow^{\dagger}, -, -$
Cytokine-Cytokine receptor interaction [KEGG]	T-cell	0.11 (↑)	IL13, CCR2, TNFSF10, CXCL13, IL1R2	0.28 (↑), 0.22 (↓), 0.12 (↑), 0.09 (↑), 0.01 (↑)	$\uparrow, \downarrow, \uparrow, \uparrow^{\dagger}, \uparrow$
	Dendritic Cell	0.32 (↑)	IL23A, CCR6, CCL5, CCL3L1, TNFRSF14	0.11 (↓), 0.06 (↓), 0.06 (↓), 0.05 (↓), 0.05 (↑)	$\uparrow^{\dagger}, \downarrow, \downarrow^{e,\dagger}, \downarrow^{\dagger}, \uparrow$
	Langerhans Cell	0.36 (↑)	IL22RA2, PLEKHO2, IL23A, IL7R, CCR1	0.06 (↑), 0.04 (↑), 0.04 (↓), 0.03 (↑), 0.03 (↑)	$\uparrow, \uparrow^{\dagger}, \downarrow^{\dagger}, \uparrow, \uparrow$
	Keratinocyte	0.11 (↑)	IL15RA, TNFRSF12A, CCR2, TNFRSF11A, IFNAR2	0.41 (↑), 0.17 (↑), 0.12 (↑), 0.04 (↓), 0.05 (↑)	$\uparrow^{*}, \uparrow, \uparrow, -, \uparrow^{*}$
	Melanocyte	0.10 (↑)	CX3CL1, TGFB2, IFNGR2, TNFSF13B, IL10RA	0.32 (↓), 0.28 (↓), 0.08 (↑), 0.08 (↑), 0.05 (↑)	$\downarrow^{*,\dagger}, \downarrow^{\dagger}, \uparrow^{*}, -, \uparrow^{*}$
Chemokine receptors bind chemokines [REACTOME]	T-cell	0.11 (↑)	CCR2, CXCL13, CCR1, CXCL8, CCR7	0.42 (↓), 0.31 (↑), 0.12 (↑), 0.07 (↑), 0.03 (↓)	$\downarrow, \uparrow^{\dagger}, \uparrow, \uparrow \downarrow$
	Dendritic Cell	0.32 (↑)	CCL5, CCR6, CCL3L1, CCL13, CXCL2	0.07 (↓), 0.07 (↓), 0.07 (↓), 0.06 (↑), 0.06 (↓)	$\downarrow^{e,\dagger}, \downarrow, \downarrow^{\dagger}, \uparrow^{\dagger}, -$
	Langerhans Cell	0.17 (↑)	CCR1, CCL17, CXCR4, CCR10, CCRL2	0.17 (↑), 0.13 (↑), 0.12 (↑), 0.10 (↓), 0.08 (↑)	$\uparrow, \uparrow, \uparrow, -, \uparrow^{\dagger}$
	Keratinocyte	0.36 (↑)	CCR2, CXCL2, CCL7, XCR1, CCL20	0.07 (↑), 0.06 (↑), 0.05 (↑), 0.04 (↑), 0.04 (↑)	$\uparrow, -, \uparrow^{\dagger}, -, \uparrow$
	Melanocyte	0.04 (↑)	CX3CL1, CXCL8, CXCR6	0.95 (↓), 0.03 (↑), 0.02 (↑)	$\downarrow^{*,\dagger}, \uparrow^{\dagger}, \uparrow^{\dagger}$

a. Only genes with $FDR \leq 0.05$ and maximum GIP_g^b magnitude. *b*. ↑ for upregulated genes in literature, ↓ for supressed and − for unknown/inconsistent direction. *c*. References on direction are in Supplementary Material S3, Table S3. *d*. ∗: not reported in human skin AD but reported gene's deregulation effect in specific cell type, disease models or human AD PBMC. †: reported in bulk but not cell type. *e*. in chronic AD skin lesions.

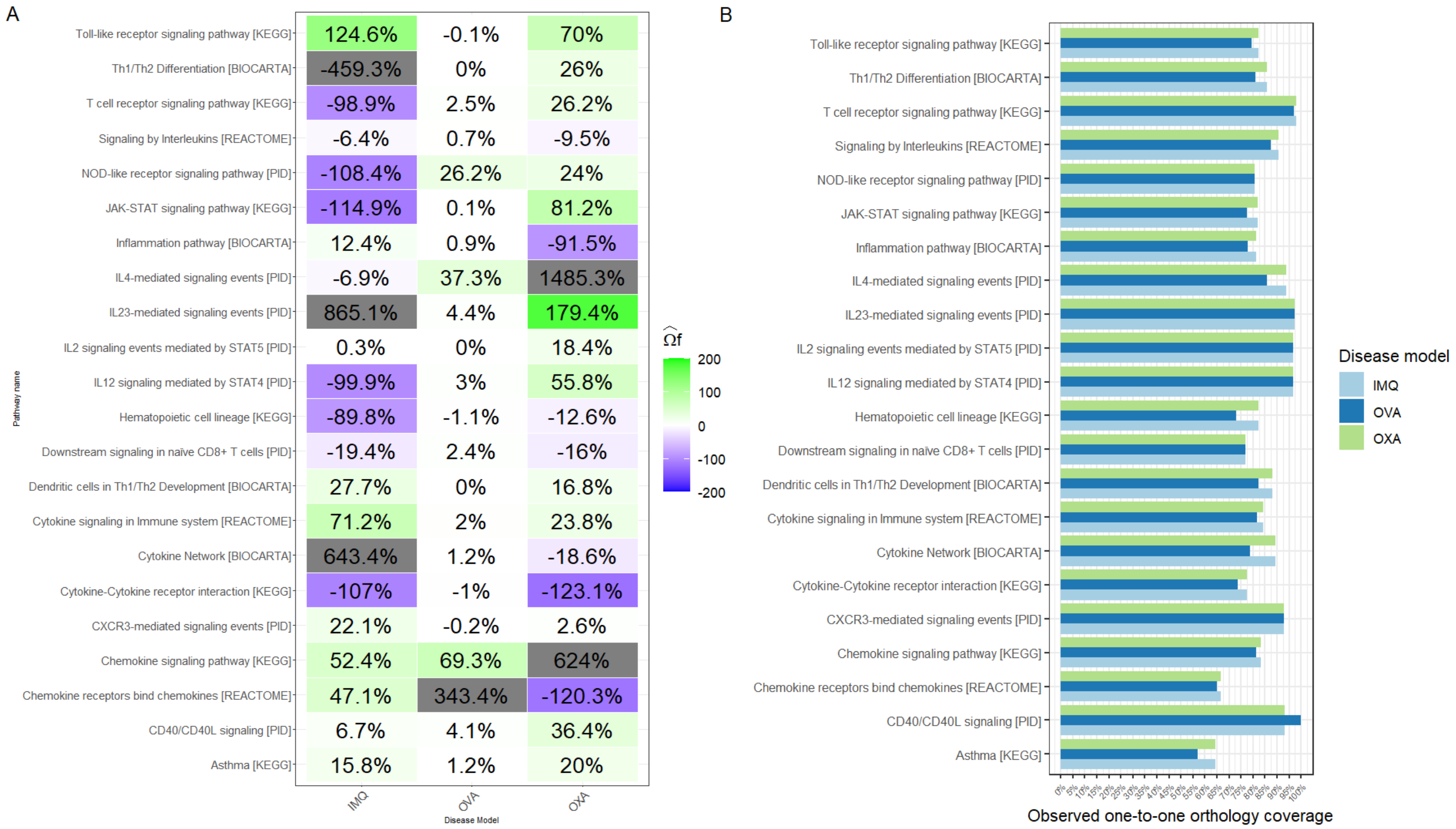

Fig A. Superpathway recapitulation and observed one-to-one orthology of AD disease models. A) Superpathway predicted recapitulation as a fraction of the superpathway reference recapitulation for IMQ, OXA and OVA across all pathways under study. Negative recapitulations refer to opposed directions with human observed condition, while positive recapitulations define agreement in direction. **B)** Observed one-to-one orthology coverage refers to number of observed and one-to-one ortholog genes in disease model as a fraction of pathway gene set size. Despite all disease models belong to the same organism *mus musculus* their differences in observed orthology one-to-one coverage come from sequenced reads.

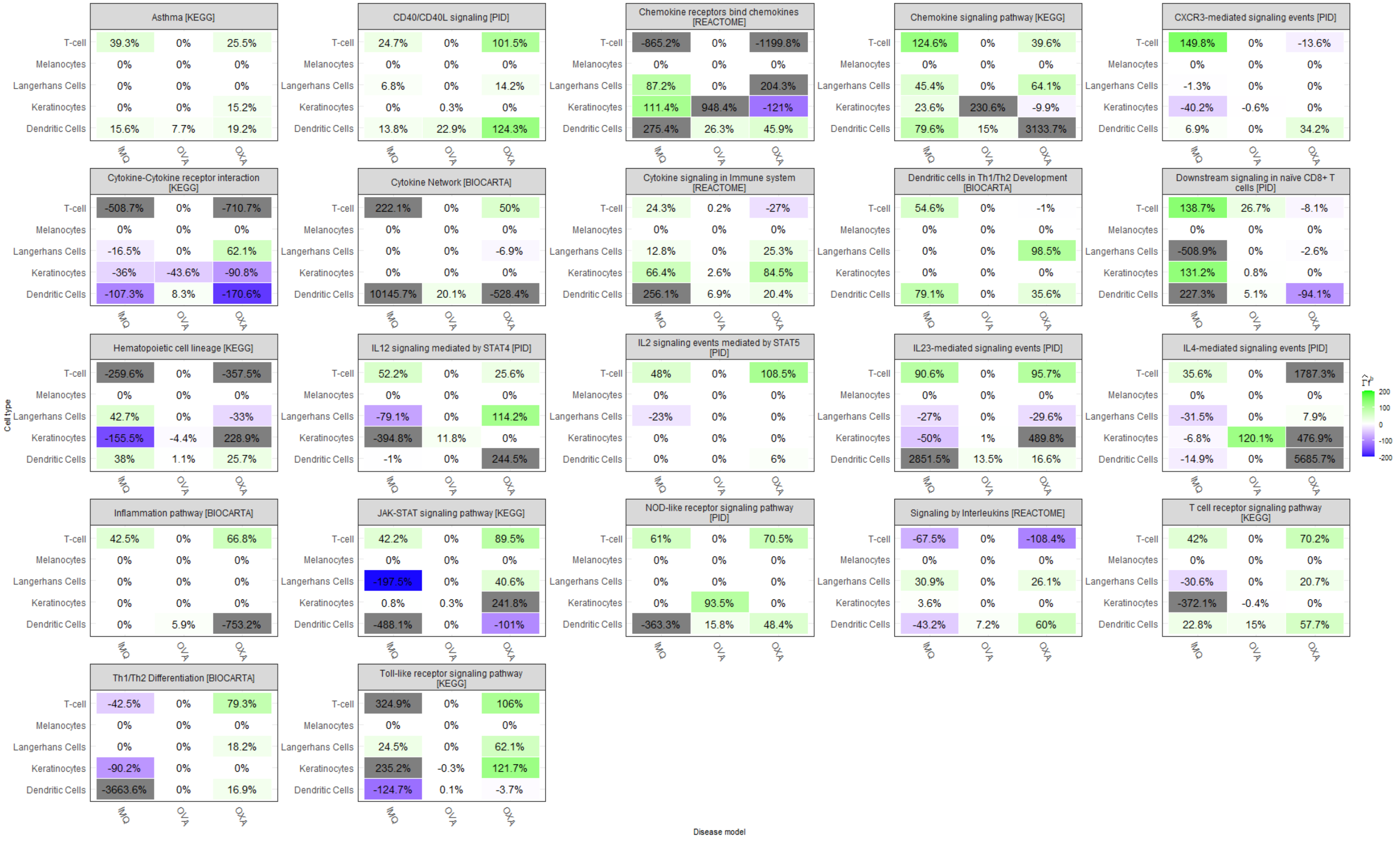

Fig B. Cell type recapitulation for all AD disease models and pathways under analysis.

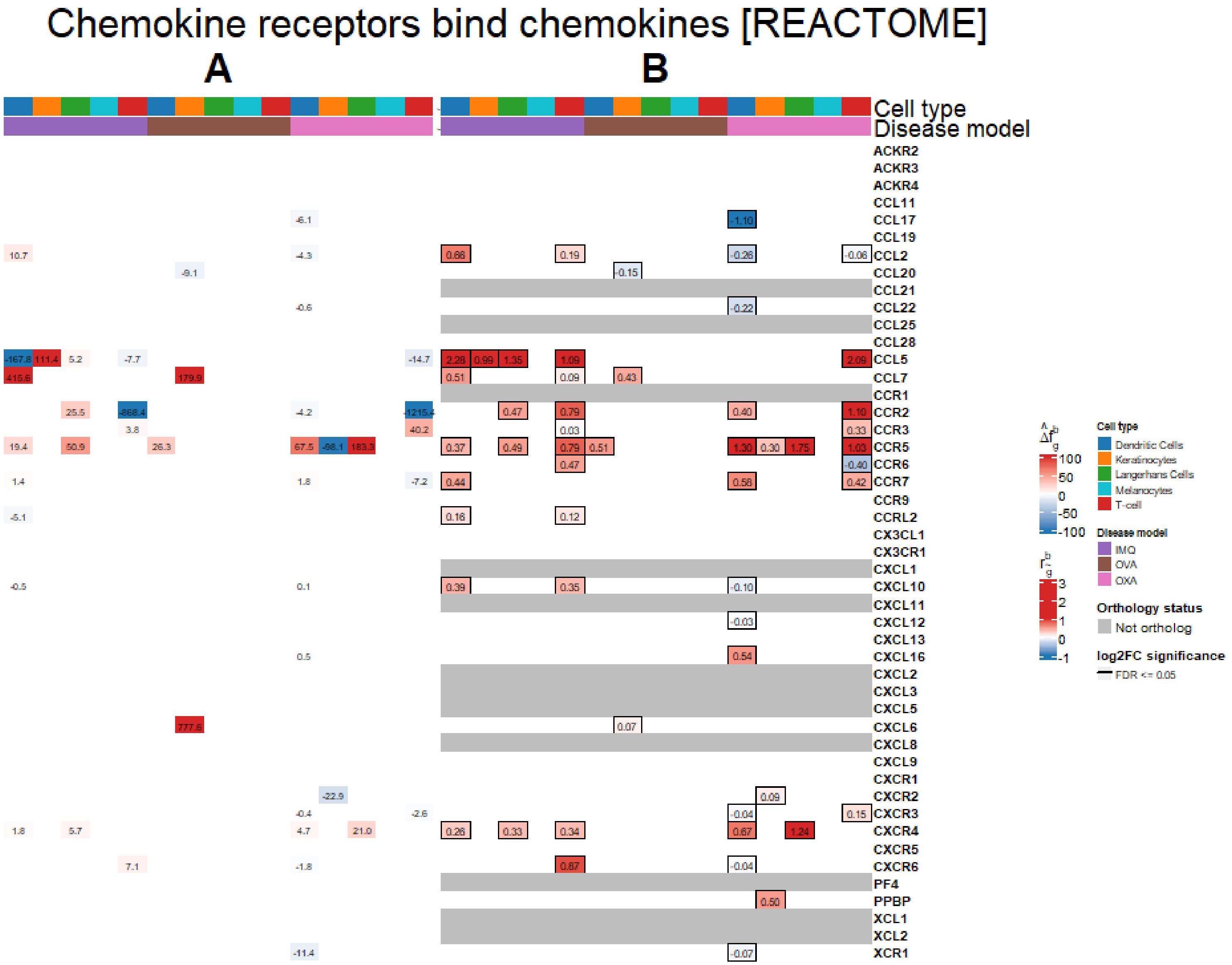
Chemokine receptors bind chemokines [REACTOME]
A
B
Cell type
Disease model
ACKR2
ACKR3
ACKR4
CCL11
CCL17
CCL19
CCL2
CCL20
CCL21
CCL22
CCL25
CCL28
CCL5
CCL7
CCR1
CCR2
CCR3
CCR5
CCR6
CCR7
CCR9
CCRL2
CX3CL1
CX3CR1
CXCL1
CXCL10
CXCL11
CXCL12
CXCL13
CXCL16
CXCL2
CXCL3
CXCL5
CXCL6
CXCL8
CXCL9
CXCR1
CXCR2
CXCR3
CXCR4
CXCR5
CXCR6
PF4
PPBP
XCL1
XCL2
XCR1
Cell type
Dendritic Cells
Keratinocytes
Langerhans Cells
Melanocytes
T-cell
Disease model
IMQ
OVA
OXA
Orthology status
Not ortholog
log2FC significance
FDR <= 0.05
100
50
0
-50
-100
3
2
1
0
-1

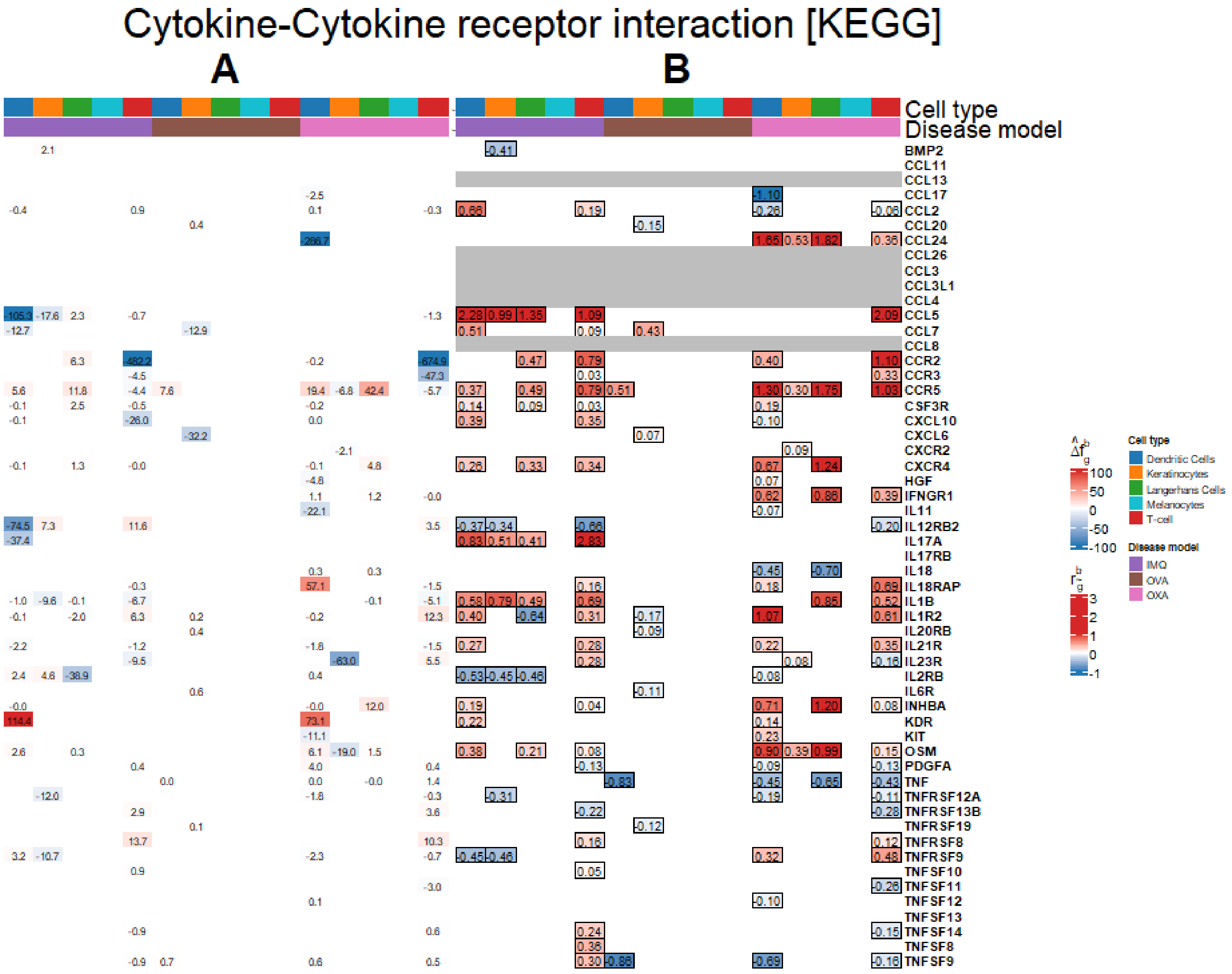
Cytokine-Cytokine receptor interaction [KEGG]
A
B
Cell type
Disease model
BMP2
CCL11
CCL13
CCL17
CCL2
CCL20
CCL24
CCL26
CCL3
CCL3L1
CCL4
CCL5
CCL7
CCL8
CCR2
CCR3
CCR5
CSF3R
CXCL10
CXCL6
CXCR2
CXCR4
HGF
IFNGR1
IL11
IL12RB2
IL17A
IL17RB
IL18
IL18RAP
IL1B
IL1R2
IL20RB
IL21R
IL23R
IL2RB
IL6R
INHBA
KDR
KIT
OSM
PDGFA
TNF
TNFRSF12A
TNFRSF13B
TNFRSF19
TNFRSF8
TNFRSF9
TNFSF10
TNFSF11
TNFSF12
TNFSF13
TNFSF14
TNFSF8
TNFSF9
100
50
0
-50
-100
3
2
1
0
-1
Cell type
Dendritic Cells
Keratinocytes
Langerhans Cells
Melanocytes
T-cell
Disease model
IMQ
OVA
OXA

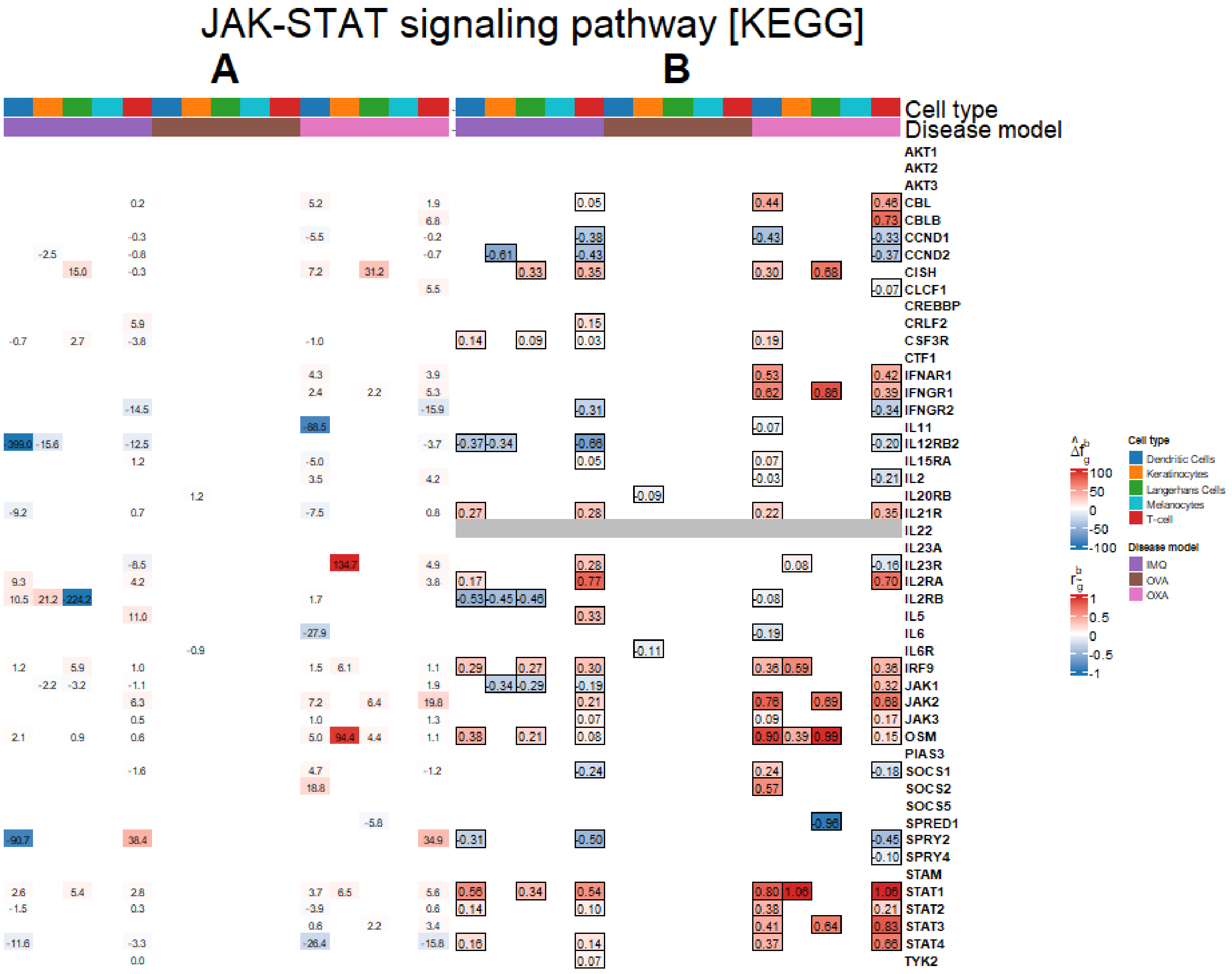
JAK-STAT signaling pathway [KEGG]
A
B
Cell type
Disease model
AKT1
AKT2
AKT3
CBL
CBLB
CCND1
CCND2
CISH
CLCF1
CREBBP
CRLF2
CSF3R
CTF1
IFNAR1
IFNGR1
IFNGR2
IL11
IL12RB2
IL15RA
IL2
IL20RB
IL21R
IL22
IL23A
IL23R
IL2RA
IL2RB
IL5
IL6
IL6R
IRF9
JAK1
JAK2
JAK3
OSM
PIAS3
SOCS1
SOCS2
SOCS5
SPRED1
SPRY2
SPRY4
STAM
STAT1
STAT2
STAT3
STAT4
TYK2
100
50
0
-50
-100
1
0.5
0
-0.5
-1
Cell type
Dendritic Cells
Keratinocytes
Langerhans Cells
Melanocytes
T-cell
Disease model
IMQ
OVA
OXA

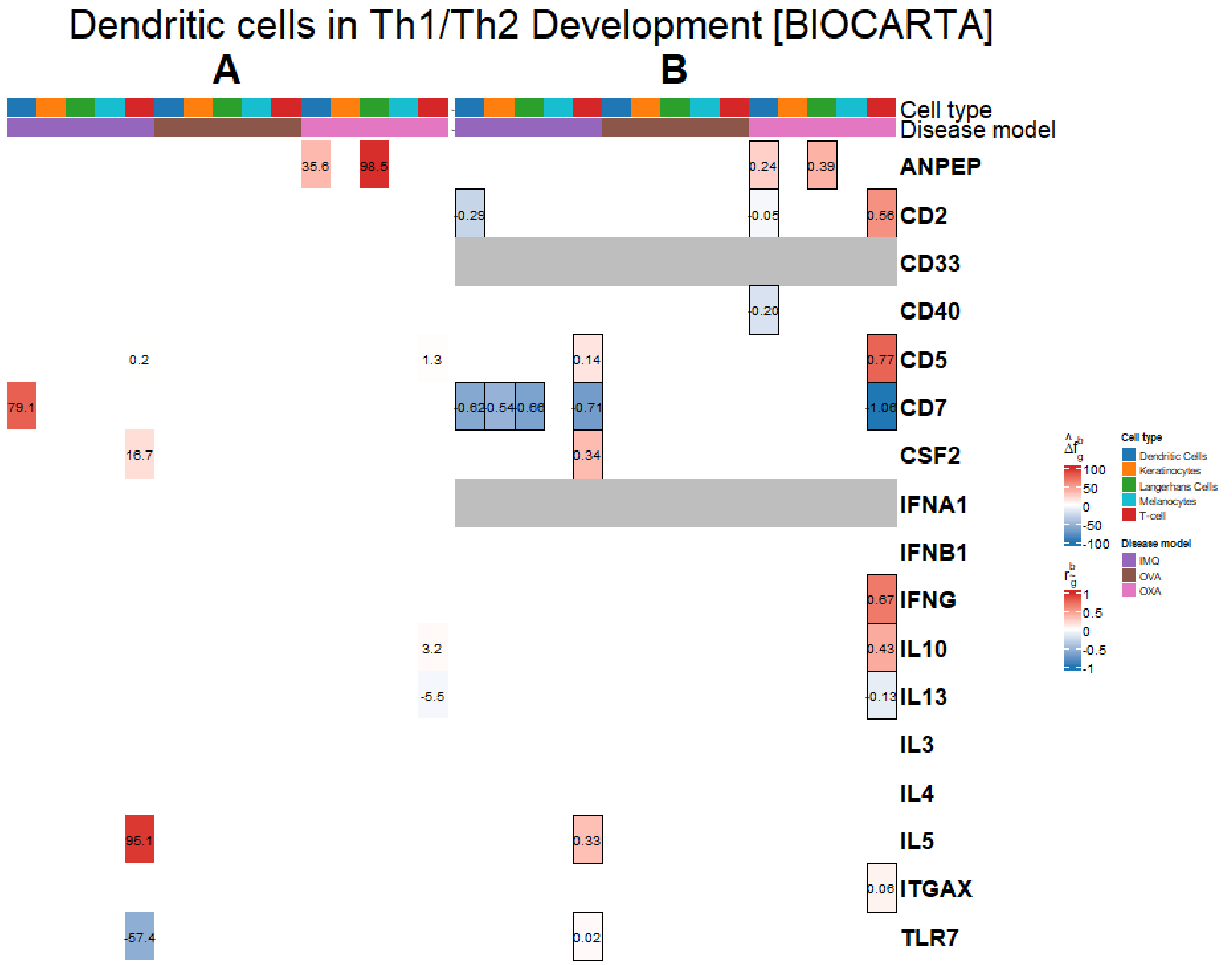

Fig C. Gene contribution and disease model estimated $r^b_{\tilde{g}}$. **A)** Gene contribution to cell type recapitulation by disease model. If gene set size of pathway is greater than 50, only the top 20 contributing genes, for each cell type, were displayed. Blank gene contributions correspond to 0 values. **B)** Computed $r^b_{\tilde{g}}$ by disease model. Grey FC refer to genes without one-to-one ortholog and/or not sequenced in disease model. Framed FC refer to statistically significant $FDR \leq 0.05$ genes, as per FindMarkers. Blank FC correspond to 0 values.

Table C. References of Manuscript's Table 2

Pathway	Cell type	Top 5 genes	Reference direction
JAK-STAT signaling pathway [KEGG]	T-cell	IL13, IL26, IL2RA, IL7, IFNGR1	(Napolitano et al., 2023), (Kamijo et al., 2020), (Jia et al., 2023) (Park et al., 2023), (Wasserer et al., 2024)
	Dendritic Cell	IL23A, SPRED1,SOCS1, IFNL1, OSM	(Napolitano et al., 2021), (Sakai et al., 2022), (Kopalli et al., 2022) (Philip et al., 2024), (Suehiro et al., 2023)
	Langerhan Cell	IL22RA2, CCND2, CCND1, JAK1, STAT6	(Bangert et al., 2024), NA, NA, (Huang et al., 2022), (Antczak et al., 2016)
	Keratinocyte	CCND3, SPRY1, IL15RA, IFNAR2, IL15	NA, (Cui et al., 2024), (Jones et al., 2016) (Morizane et al., 2023), (Karlen et al., 2020)
	Melanocyte	CCND3, IFNGR2, CCND2, IL10RA, CCND1	(Alekseenko et al., 2010), (Su et al., 2020), NA (Zhou et al., 2016), (Alekseenko et al., 2010)
Dendritic Cells in Th1/Th2 development [BIOCARTA]	T-cell	IL13, IL5, CSF2, TLR7	(Napolitano et al., 2023), (Antosz et al., 2024), [(Mu et al., 2021), (Xing et al., 1997)], (Jeisy-Scott et al., 2011)
	Dendritic Cell	ANPEP, CSF2, IL13	(Lu et al., 2020), (Taha et al., 1998), (Lamiable et al., 2022)
	Langerhan Cell	ANPEP	(Lu et al., 2020)
	Keratinocyte	ITGAX	(Zhong et al., 2021)
	Melanocyte	IL10, ITGAX, CD7, CD33	(Ohmen et al., 1995), (Zhong et al., 2021), NA, NA
Cytokine-Cytokine receptor interaction [KEGG]	T-cell	IL13, CCR2, TNFSF10, CXCL13, IL1R2	(Napolitano et al., 2023), [(Bakos et al., 2017), (Nedoszytko et al., 2014)], (Vassina et al., 2005), (Kwon et al., 2021), (Yamamoto-Hanada et al., 2023)
	Dendritic Cell	IL23A, CCR6, CCL5, CCL3L1, TNFRSF14	(Napolitano et al., 2021), (Gros et al., 2009), (Tsoi et al., 2020) (Alkon et al., 2022), (Bangert et al., 2021)
	Langerhan Cell	IL22RA2, PLEKHO2, IL23A, IL7R, CCR1	(Bangert et al., 2024), (Nousbeck et al., 2022), (Napolitano et al., 2021) (Gonzalez-Rodriguez et al., 2022), (Nedoszytko et al., 2014)
	Keratinocyte	IL15RA, TNFRSF12A, CCR2, TNFRSF11A, IFNAR2	(Morizane et al., 2023), (Bangert et al., 2021), (Nedoszytko et al., 2014) NA, (Hile et al., 2020)
	Melanocyte	CX3CL1, TGFB2, IFNGR2, TNFSF13B, IL10RA	(Staumont-Sallé et al., 2014), (Arkwright et al., 2001), (Su et al., 2020) NA, (Zhou et al., 2016)
Chemokine receptors bind chemokines [REACTOME]	T-cell	CCR2, CXCL13, CCR1, CXCL8, CCR7	[(Bakos et al., 2017), (Nedoszytko et al., 2014)], (Kwon et al., 2021), (Nedoszytko et al., 2014) (Morgner et al., 2023), (Bangert et al., 2021)
	Dendritic Cell	CCL5, CCR6, CCL3L1, CCL13, CXCL2	(Tsoi et al., 2020), (Gros et al., 2009), (Alkon et al., 2022) (Bangert et al., 2021), NA
	Langerhan Cell	CCR1, CCL17, CXCR4, CCR10, CCRL2	(Nedoszytko et al., 2014), (Bangert et al., 2021), (Dubrac et al., 2010) NA, (Nousbeck et al., 2022)
	Keratinocyte	CCR2, CXCL2, CCL7, XCR1, CCL20	(Nedoszytko et al., 2014), NA, (Gros et al., 2009), NA, (Nakayama et al., 2001)
	Melanocyte	CX3CL1, CXCL8, CXCR6	(Staumont-Sallé et al., 2014), , (Zhang et al., 2023)

REFERENCES

A. Alekseenko, A. Wojas-Pelc, G. J. Lis, A. Furgał-Borzych, G. Surówka, and J. A. Litwin. Cyclin d1 and d3 expression in melanocytic skin lesions. *Archives of Dermatological Research*, 302(7): 545–550, Sep 2010. doi: 10.1007/s00403-010-1054-3. Epub 2010 May 23.

N. Alkon, W. M. Bauer, T. Krausgruber, I. Goh, J. Griss, V. Nguyen, B. Reininger, C. Bangert, C. Staud, P. M. Brunner, C. Bock, M. Haniffa, and G. Stingl. Single-cell analysis reveals innate lymphoid cell lineage infidelity in atopic dermatitis. *Journal of Allergy and Clinical Immunology*, 149(2):624–639, 2022. doi: 10.1016/j.jaci.2021.07.025.

A Antczak, D Domańska-Senderowska, P Górski, et al. Analysis of changes in expression of il-4/il-13/stat6 pathway and correlation with the selected clinical parameters in patients with atopic asthma. *International Journal of Immunopathology and Pharmacology*, 29(2):195–204, 2016. doi: 10.1177/0394632015623794.

Katarzyna Antosz, Joanna Batko, Marta Błażejewska, Antoni Gawor, Jakub Sleziak, and Krzysztof Gomułka. Insight into il-5 as a potential target for the treatment of allergic diseases. *Biomedicines*, 12(7), 2024. ISSN 2227-9059. doi: 10.3390/biomedicines12071531. URL https://www.mdpi.com/2227-9059/12/7/1531.

P. D. Arkwright, J. M. Chase, S. Babbage, V. Pravica, T. J. David, and I. V. Hutchinson. Atopic dermatitis is associated with a low-producer transforming growth factor beta(1) cytokine genotype. *The Journal of Allergy and Clinical Immunology*, 108(2):281–284, 2001. doi: 10.1067/mai.2001.117259.

E. Bakos, C. A. Thaiss, M. P. Kramer, S. Cohen, L. Radomir, I. Orr, N. Kaushansky, A. Ben-Nun, S. Becker-Herman, and I. Shachar. Ccr2 regulates the immune response by modulating the interconversion and function of effector and regulatory t cells. *Journal of Immunology (Baltimore, Md. : 1950)*, 198(12):4659–4671, 2017. doi: 10.4049/jimmunol.1601458.

C. Bangert, N. Alkon, S. Chennareddy, et al. Dupilumab-associated head and neck dermatitis shows a pronounced type 22 immune signature mediated by oligoclonally expanded t cells. *Nat Commun*, 15:2839, 2024. doi: 10.1038/s41467-024-46540-0.

Christine Bangert et al. Persistence of mature dendritic cells, th2a, and tc2 cells characterize clinically resolved atopic dermatitis under il-4r blockade. *Science Immunology*, 6(eabe2749), 2021. doi: 10.1126/sciimmunol.abe2749.

Ying-Zhe Cui, Fan Xu, Yuan Zhou, Zhao-Yuan Wang, Xing-Yu Yang, Ni-Chang Fu, Xi-Bei Chen, Yu-Xin Zheng, Xue-Yan Chen, Li-Ran Ye, Ying-Ying Li, and Xiao-Yong Man. Spry1 deficiency in keratinocytes induces follicular melanocyte stem cell migration to the epidermis through p53/stem cell factor/c-kit signaling. *Journal of Investigative Dermatology*, 2024. ISSN 0022-202X. doi: https://doi.org/10.1016/j.jid.2024.02.018. URL https://www.sciencedirect.com/science/article/pii/S0022202X24001787.

Sandrine Dubrac, Matthias Schmuth, and Susanne Ebner. Atopic dermatitis: the role of langerhans cells in disease pathogenesis. *Immunology and Cell Biology*, 88(4):400–409, 2010. doi: https://doi.org/10.1038/icb.2010.33. URL https://onlinelibrary.wiley.com/doi/abs/10.1038/icb.2010.33.

Martin Ignacio Gonzalez-Rodriguez, Tanja Salomaa, Laura Kummola, Lotta Hiihtola, and Ilkka Junttila. IL7R and TSLPR expression on Langerhans cells in response to inflammatory stimulation. *The Journal of Immunology*, 208(1Supplement):45.02–45.02, 05 2022. ISSN 0022-1767. doi: 10.4049/jimmunol.208.Supp.45.02. URL https://doi.org/10.4049/jimmunol.208.Supp.45.02.

E. Gros, C. Bussmann, T. Bieber, I. Förster, and N. Novak. Expression of chemokines and chemokine receptors in lesional and nonlesional upper skin of patients with atopic dermatitis. *The Journal of Allergy and Clinical Immunology*, 124(4):753–760.e1, 2009. doi: 10.1016/j.jaci.2009.07.004.

G. A. Hile, J. E. Gudjonsson, and J. M. Kahlenberg. The influence of interferon on healthy and diseased skin. *Cytokine*, 132:154605, 2020. doi: 10.1016/j.cyto.2018.11.022.

I-Hsin Huang, Wen-Hung Chung, Po-Chien Wu, and Chun-Bing Chen. Jak–stat signaling pathway in the pathogenesis of atopic dermatitis: An updated review. *Frontiers in Immunology*, 13, 2022. ISSN 1664-3224. doi: 10.3389/fimmu.2022.1068260. URL https://www.frontiersin.org/journals/immunology/articles/10.3389/fimmu.2022.1068260.

V. Jeisy-Scott, W. G. Davis, J. R. Patel, J. B. Bowzard, W. J. Shieh, S. R. Zaki, J. M. Katz, and S. Sambhara. Increased mdsc accumulation and th2 biased response to influenza a virus infection in the absence of tlr7 in mice. *PloS One*, 6(9):e25242, 2011. doi: 10.1371/journal.pone.0025242.

H Jia, H Wan, and D Zhang. Innate lymphoid cells: a new key player in atopic dermatitis. *Front Immunol*, 14:1277120, Oct 2023. doi: 10.3389/fimmu.2023.1277120.

A. M. Jones, J. L. Griffiths, A. J. Sanders, S. Owen, F. Ruge, K. G. Harding, and W. G. Jiang. The clinical significance and impact of interleukin 15 on keratinocyte cell growth and migration. *International Journal of Molecular Medicine*, 38(3):679–686, Sep 2016. doi: 10.3892/ijmm.2016.2687. Epub 2016 Jul 21.

Hiroaki Kamijo, Tomomitsu Miyagaki, Yoshio Hayashi, Taro Akatsuka, Sayaka Watanabe-Otobe, Tomonori Oka, Naomi Shishido-Takahashi, Hiraku Suga, Makoto Sugaya, and Shinichi Sato. Increased il-26 expression promotes t helper type 17- and t helper type 2-associated cytokine production by keratinocytes in atopic dermatitis. *Journal of Investigative Dermatology*, 140 (3):636–644.e2, 2020. ISSN 0022-202X. doi: https://doi.org/10.1016/j.jid.2019.07.713. URL https://www.sciencedirect.com/science/article/pii/S0022202X19331483.

H. Karlen, S. Yousefi, H. U. Simon, and D. Simon. Il-15 expression pattern in atopic dermatitis. *International Archives of Allergy and Immunology*, 181(6):417–421, 2020. doi: 10.1159/000508515.

Spandana Rajendra Kopalli, Venkata Prakash Annamneedi, and Sushruta Koppula. Potential natural biomolecules targeting jak/stat/socs signaling in the management of atopic dermatitis. *Molecules*, 27(14), 2022. ISSN 1420-3049. doi: 10.3390/molecules27144660. URL https://www.mdpi.com/1420-3049/27/14/4660.

Y. Kwon, Y. Choi, M. Kim, M. S. Jeong, H. S. Jung, and D. Jeoung. Hdac6 and cxcl13 mediate atopic dermatitis by regulating cellular interactions and expression levels of mir-9 and sirt1. *Frontiers in Pharmacology*, 12:691279, 2021. doi: 10.3389/fphar.2021.691279.

O. Lamiable, M. Brewerton, and F. Ronchese. Il-13 in dermal type-2 dendritic cell specialization: From function to therapeutic targeting. *European Journal of Immunology*, 52(7):1047–1057, 2022. doi: 10.1002/eji.202149677.

Chenyang Lu, Mohammad A. Amin, and David A. Fox. CD13/Aminopeptidase N Is a Potential Therapeutic Target for Inflammatory Disorders. *The Journal of Immunology*, 204(1):3–11, 01 2020. ISSN 0022-1767. doi: 10.4049/jimmunol.1900868. URL https://doi.org/10.4049/jimmunol.1900868.

B. Morgner, J. Tittelbach, and C. Wiegand. Induction of psoriasis- and atopic dermatitis-like phenotypes in 3d skin equivalents with a fibroblast-derived matrix. *Scientific Reports*, 13:1807, 2023. doi: 10.1038/s41598-023-28822-7.

S. Morizane, T. Mukai, K. Sunagawa, K. Tachibana, Y. Kawakami, and M. Ouchida. "input/output cytokines" in epidermal keratinocytes and the involvement in inflammatory skin diseases. *Frontiers in Immunology*, 14:1239598, Oct 2023. doi: 10.3389/fimmu.2023.1239598.

X. Mu, K. Liu, H. Li, et al. Granulocyte-macrophage colony-stimulating factor: an immunotarget for sepsis and covid-19. *Cellular and Molecular Immunology*, 18:2057–2058, 2021. doi: 10.1038/s41423-021-00719-3.

T. Nakayama, R. Fujisawa, H. Yamada, T. Horikawa, H. Kawasaki, K. Hieshima, D. Izawa, S. Fujiie, T. Tezuka, and O. Yoshie. Inducible expression of a cc chemokine liver- and activation-regulated chemokine (larc)/macrophage inflammatory protein (mip)-3 alpha/ccl20 by epidermal keratinocytes and its role in atopic dermatitis. *International Immunology*, 13(1):95–103, 2001. doi: 10.1093/intimm/13.1.95.

M. Napolitano, G. Caiazzo, G. Fabbrocini, A. Balato, R. Di Caprio, E. Scala, M. Scalvenzi, and C. Patruno. Increased expression of interleukin-23A in lesional skin of patients with atopic dermatitis with psoriasiform reaction during dupilumab treatment. *British Journal of Dermatology*, 184(2): 341–343, 02 2021. ISSN 0007-0963. doi: 10.1111/bjd.19459. URL https://doi.org/10.1111/bjd.19459.

M Napolitano, F di Vico, A Ruggiero, G Fabbrocini, and C Patruno. The hidden sentinel of the skin: An overview on the role of interleukin-13 in atopic dermatitis. *Front Med (Lausanne)*, 10: 1165098, Apr 2023. doi: 10.3389/fmed.2023.1165098.

B. Nedoszytko, M. Sokołowska-Wojdyło, K. Ruckemann-Dziurdzińska, J. Roszkiewicz, and R. J. Nowicki. Chemokines and cytokines network in the pathogenesis of the inflammatory skin diseases: atopic dermatitis, psoriasis and skin mastocytosis. *Postepy Dermatol Alergol*, 31(2): 84–91, 2014. doi: 10.5114/pdia.2014.40920.

J. Nousbeck, M. A. McAleer, and A. D. Irvine. Peripheral blood gene expression profile of infants with atopic dermatitis. *JID Innovations*, 3(2):100165, 2022. doi: 10.1016/j.xjidi.2022.100165.

J D Ohmen, J M Hanifin, B J Nickoloff, T H Rea, R Wyzykowski, J Kim, D Jullien, T McHugh, A S Nassif, and S C Chan. Overexpression of IL-10 in atopic dermatitis. Contrasting cytokine patterns with delayed-type hypersensitivity reactions. *The Journal of Immunology*, 154(4):1956–1963, 02 1995. ISSN 0022-1767. doi: 10.4049/jimmunol.154.4.1956. URL https://doi.org/10.4049/jimmunol.154.4.1956.

HJ Park, SW Lee, L Van Kaer, MS Lee, and S Hong. Il-7 deficiency exacerbates atopic dermatitis in nc/nga mice. *Int J Mol Sci*, 24(12):9956, Jun 2023. doi: 10.3390/ijms24129956.

D. T. Philip, N. M. Goins, N. J. Catanzaro, I. Misumi, J. K. Whitmire, H. M. Atkins, and H. M. Lazear. Interferon lambda restricts herpes simplex virus skin disease by suppressing neutrophil-mediated pathology. *mBio*, 15(4):e0262323, 2024. doi: 10.1128/mbio.02623-23.

Hiroyasu Sakai, Ken Sato, Koya Ito, Ikoi Kosugi, Miho Kiyama, Risako Kon, Nobutomo Ikarashi, Junzo Kamei, Yoshihiko Chiba, and Tomoo Hosoe. Inhibition of spred/sprouty expression in the skin of a contact dermatitis-like model. *Biological and Pharmaceutical Bulletin*, 45(8):1208–1212, 2022. doi: 10.1248/bpb.b22-00279.

D. Staumont-Sallé, S. Fleury, A. Lazzari, O. Molendi-Coste, N. Hornez, C. Lavogiez, A. Kanda, J. Wartelle, A. Fries, D. Pennino, C. Mionnet, J. Prawitt, E. Bouchaert, E. Delaporte, N. Glaichenhaus, B. Staels, V. Julia, and D. Dombrowicz. Cxcl1 (fractalkine) and its receptor cxcr1 regulate atopic dermatitis by controlling effector t cell retention in inflamed skin. *Journal of Experimental Medicine*, 211(6):1185–1196, 2014. doi: 10.1084/jem.20121350.

Q. Su, F. Wang, Z. Dong, M. Chen, and R. Cao. Ifn- induces apoptosis in human melanocytes by activating the jak1/stat1 signaling pathway. *Molecular Medicine Reports*, 22(4):3111–3116, Oct 2020. doi: 10.3892/mmr.2020.11403. Epub 2020 Aug 3.

Masataka Suehiro, Tomofumi Numata, Ryo Saito, Nozomi Yanagida, Chie Ishikawa, Kazue Uchida, Tomoko Kawaguchi, Yuhki Yanase, Yozo Ishiuji, John McGrath, and Akio Tanaka. Oncostatin m suppresses il31ra expression in dorsal root ganglia and interleukin-31-induced itching. *Frontiers in Immunology*, 14, 2023. ISSN 1664-3224. doi: 10.3389/fimmu.2023.1251031. URL https://www.frontiersin.org/journals/immunology/articles/10.3389/fimmu.2023.1251031.

R. A. Taha, D. Y. Leung, O. Ghaffar, M. Boguniewicz, and Q. Hamid. In vivo expression of cytokine receptor mrna in atopic dermatitis. *The Journal of Allergy and Clinical Immunology*, 102 (2):245–250, 1998. doi: 10.1016/s0091-6749(98)70093-4.

L. C. Tsoi, E. Rodriguez, D. Stölzl, U. Wehkamp, J. Sun, S. Gerdes, M. K. Sarkar, M. Hübenthal, C. Zeng, R. Uppala, X. Xing, F. Thielking, A. C. Billi, W. R. Swindell, A. Shefler, J. Chen, M. T. Patrick, P. W. Harms, J. M. Kahlenberg, B. E. Perez White, and S. Weidinger. Progression of acute-to-chronic atopic dermatitis is associated with quantitative rather than qualitative changes in cytokine responses. *The Journal of Allergy and Clinical Immunology*, 145(5):1406–1415, 2020. doi: 10.1016/j.jaci.2019.11.047.

E. Vassina, M. Leverkus, S. Yousefi, L. R. Braathen, H. U. Simon, and D. Simon. Increased expression and a potential anti-inflammatory role of trail in atopic dermatitis. *The Journal of Investigative Dermatology*, 125(4):746–752, 2005. doi: 10.1111/j.0022-202X.2005.23878.x.

Sophia Wasserer, Manja Jargosch, Kristine E. Mayer, Jessica Eigemann, Theresa Raunegger, Görkem Aydin, Stefanie Eyerich, Tilo Biedermann, Kilian Eyerich, and Felix Lauffer. Characterization of high and low ifng-expressing subgroups in atopic dermatitis. *International Journal of Molecular Sciences*, 25(11), 2024. ISSN 1422-0067. doi: 10.3390/ijms25116158. URL https://www.mdpi.com/1422-0067/25/11/6158.

Z. Xing, J. Gauldie, G. M. Tremblay, B. R. Hewlett, and C. Addison. Intradermal transgenic expression of granulocyte-macrophage colony-stimulating factor induces neutrophilia, epidermal hyperplasia, langerhans' cell/macrophage accumulation, and dermal fibrosis. *Laboratory Investigation; a Journal of Technical Methods and Pathology*, 77(6):615–622, 1997.

Kiwako Yamamoto-Hanada, Mayako Saito-Abe, Kyoko Shima, Satoko Fukagawa, Yuya Uehara, Yui Ueda, Maeko Iwamura, Takatoshi Murase, Tetsuya Kuwano, Takayoshi Inoue, and Yukihiro Ohya. mrnas in skin surface lipids unveiled atopic dermatitis at 1 month. *Journal of the European Academy of Dermatology and Venereology*, 37(7):1385–1395, 2023. doi: https://doi.org/10.1111/jdv.19017. URL https://onlinelibrary.wiley.com/doi/abs/10.1111/jdv.19017.

Bowen Zhang, Lennart M. Roesner, Stephan Traidl, Valerie A. C. M. Koeken, Cheng-Jian Xu, Thomas Werfel, and Yang Li. Single-cell profiles reveal distinctive immune response in atopic dermatitis in contrast to psoriasis. *Allergy*, 78(2):439–453, 2023. doi: https://doi.org/10.1111/all.15486. URL https://onlinelibrary.wiley.com/doi/abs/10.1111/all.15486.

Y. Zhong, K. Qin, L. Li, H. Liu, Z. Xie, and K. Zeng. Identification of immunological biomarkers of atopic dermatitis by integrated analysis to determine molecular targets for diagnosis and therapy. *International Journal of General Medicine*, 14:8193–8209, Nov 2021. doi: 10.2147/IJGM.S331119.

J. Zhou, J. Ling, J. Song, Y. Wang, B. Feng, and F. Ping. Interleukin 10 protects primary melanocyte by activation of stat-3 and pi3k/akt/nf-b signaling pathways. *Cytokine*, 83:275–281, 2016. doi: 10.1016/j.cyto.2016.05.013.

2. HIDRADENITIS SUPPURATIVA: EVALUATION OF SKIN EXPLANTS EX VIVO

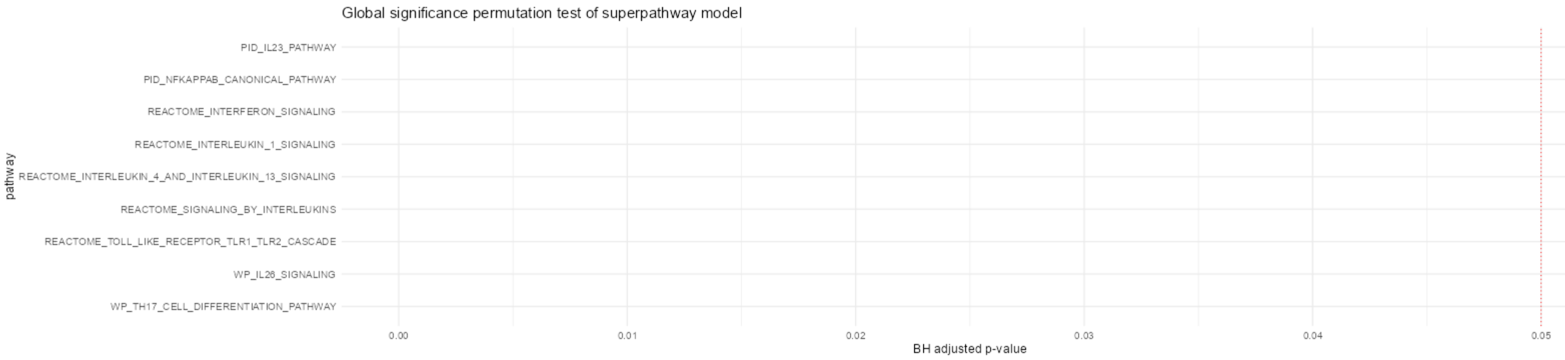

Fig D. Global permutation test of optimal HS asmbPLS-DA. To train all the optimal models a 3-Fold CV with 20 repetitions was performed. Hyperparameters were set to 5 PLS components (maximum number to search for), expected measure increase of 0.05 (maximum number of improvement to add another PLS component), and quantiles of cell type ranging along {0.05, 0.10, . . . , 0.55, . . . , 0.90, 0.95} with 1000 combinations possible, and optimization of F1 metric. Global permutation test, along with other permutation tests for CIP and GIP, were performed with 1000 permutations.

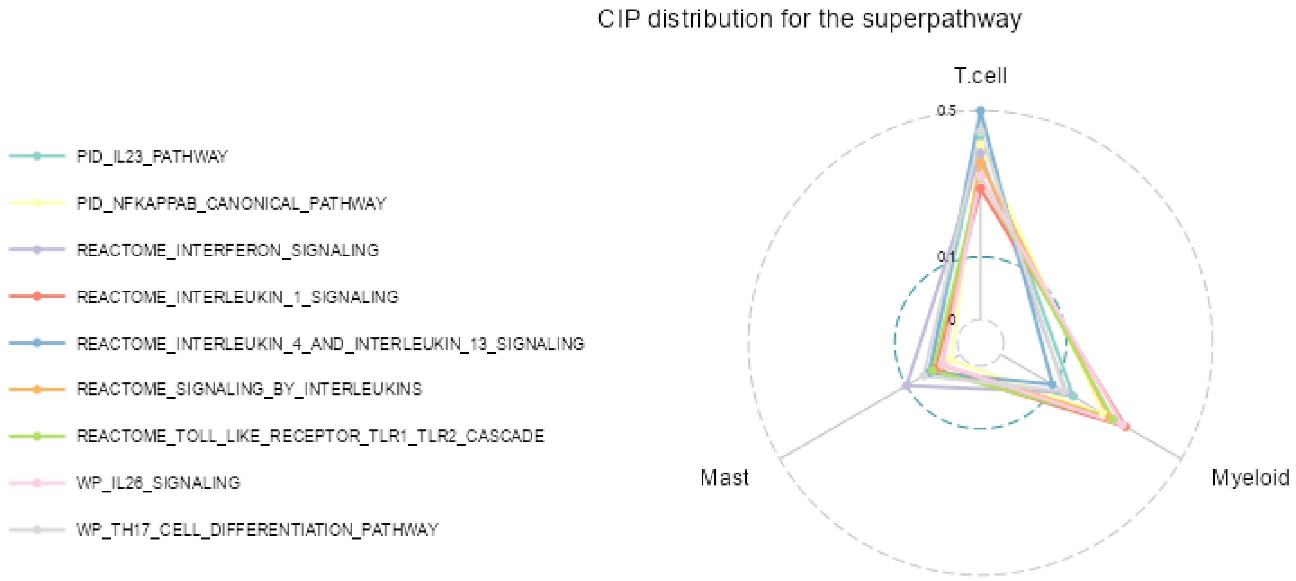

Fig E. Cell Importance Projection (CIP) distribution across all HS superpathways under analysis. These CIP distributions are from optimal asmbPLS-DA models.

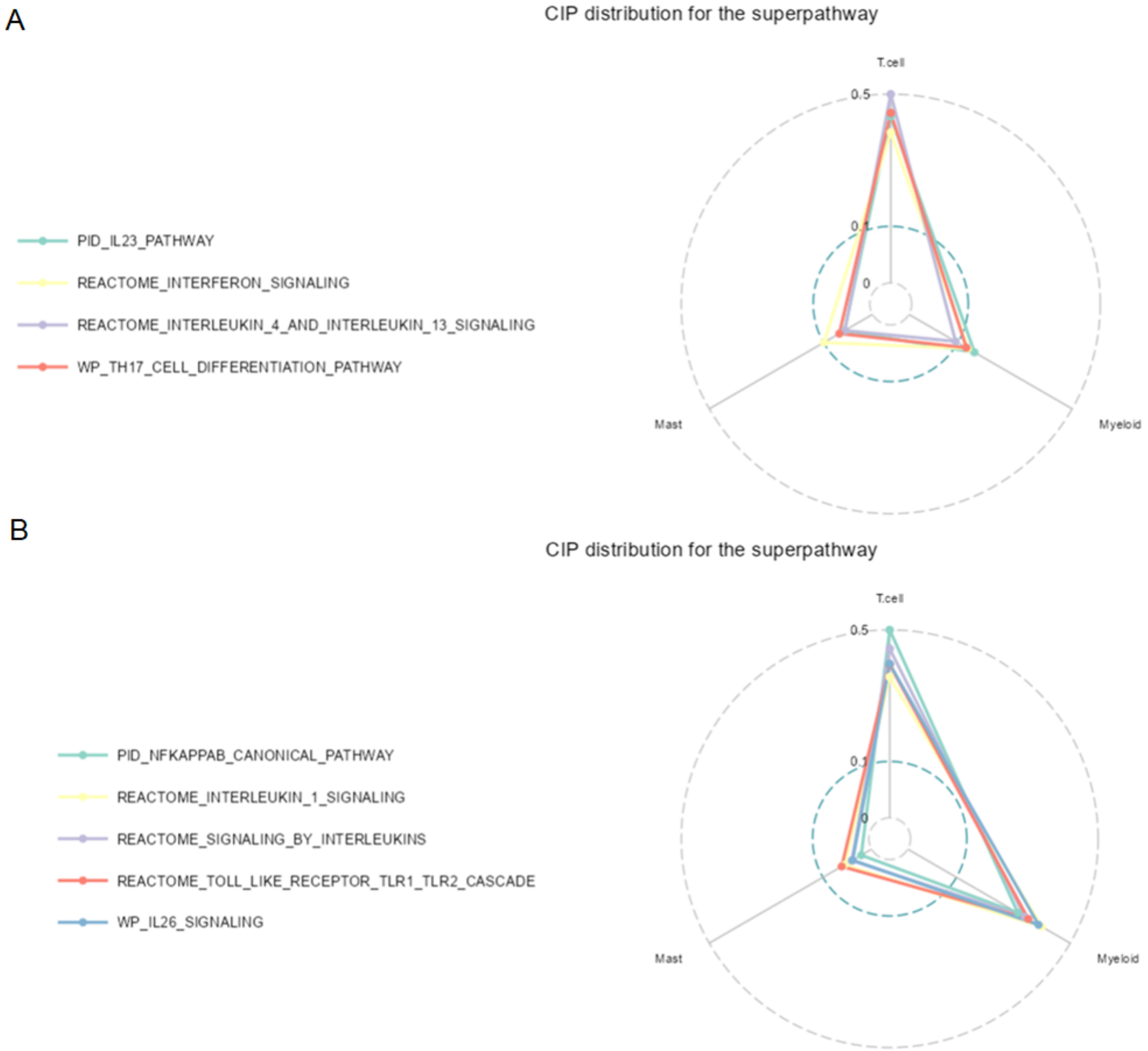

Fig F. A) HS Superpathways mainly driven by T-cell predictivity according to their CIP distribution. **B)** HS Superpathways driven by combination of T-cell and Myeloid cell types according to their CIP distribution.

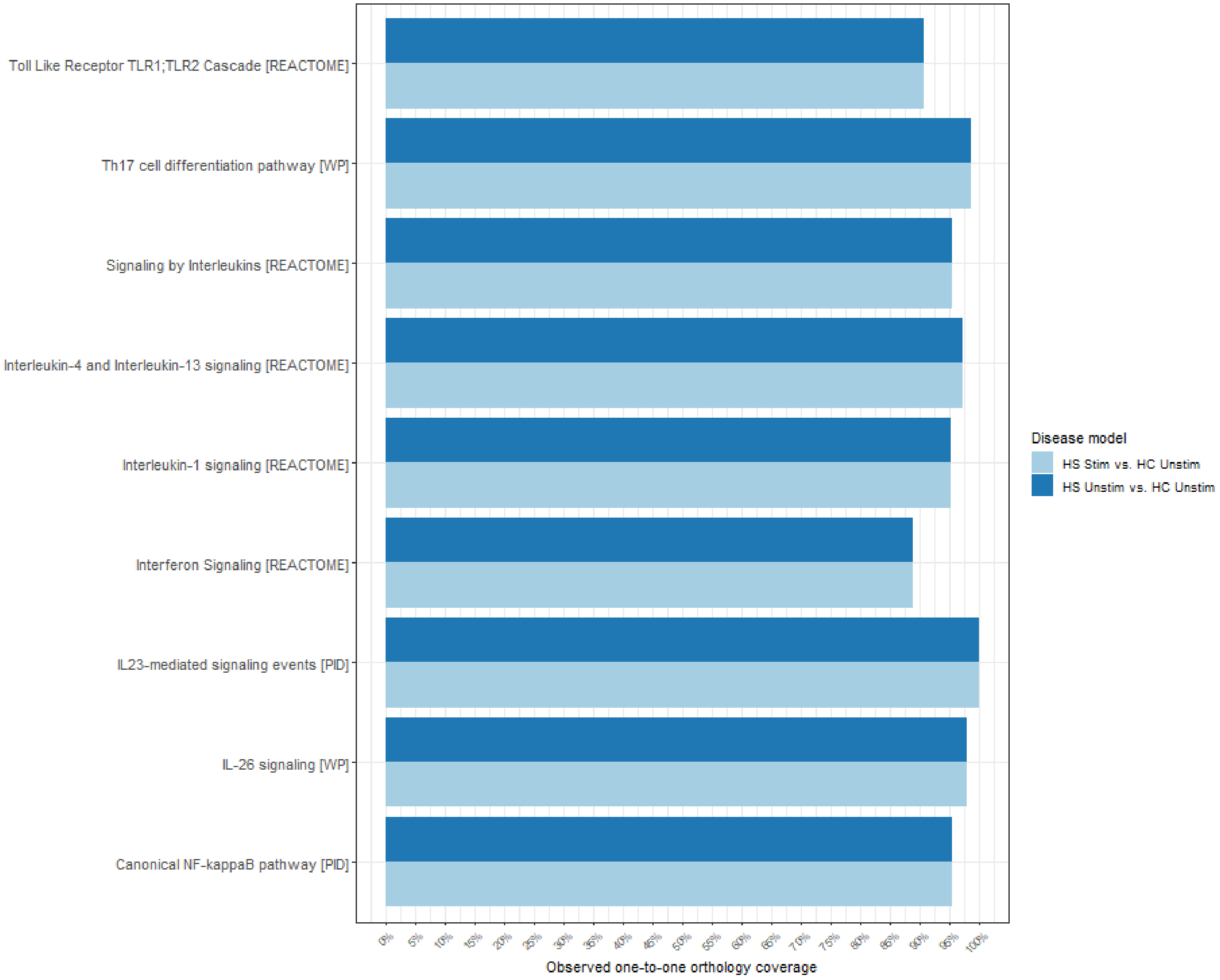

Fig G. Observed one-to-one orthology of HS disease models. Observed one-to-one orthology coverage refers to number of observed and one-to-one ortholog genes in disease model as a fraction of pathway gene set size. Despite all disease models belong to the same organism *Homo Sapiens* their differences in observed orthology one-to-one coverage come from sequenced reads.

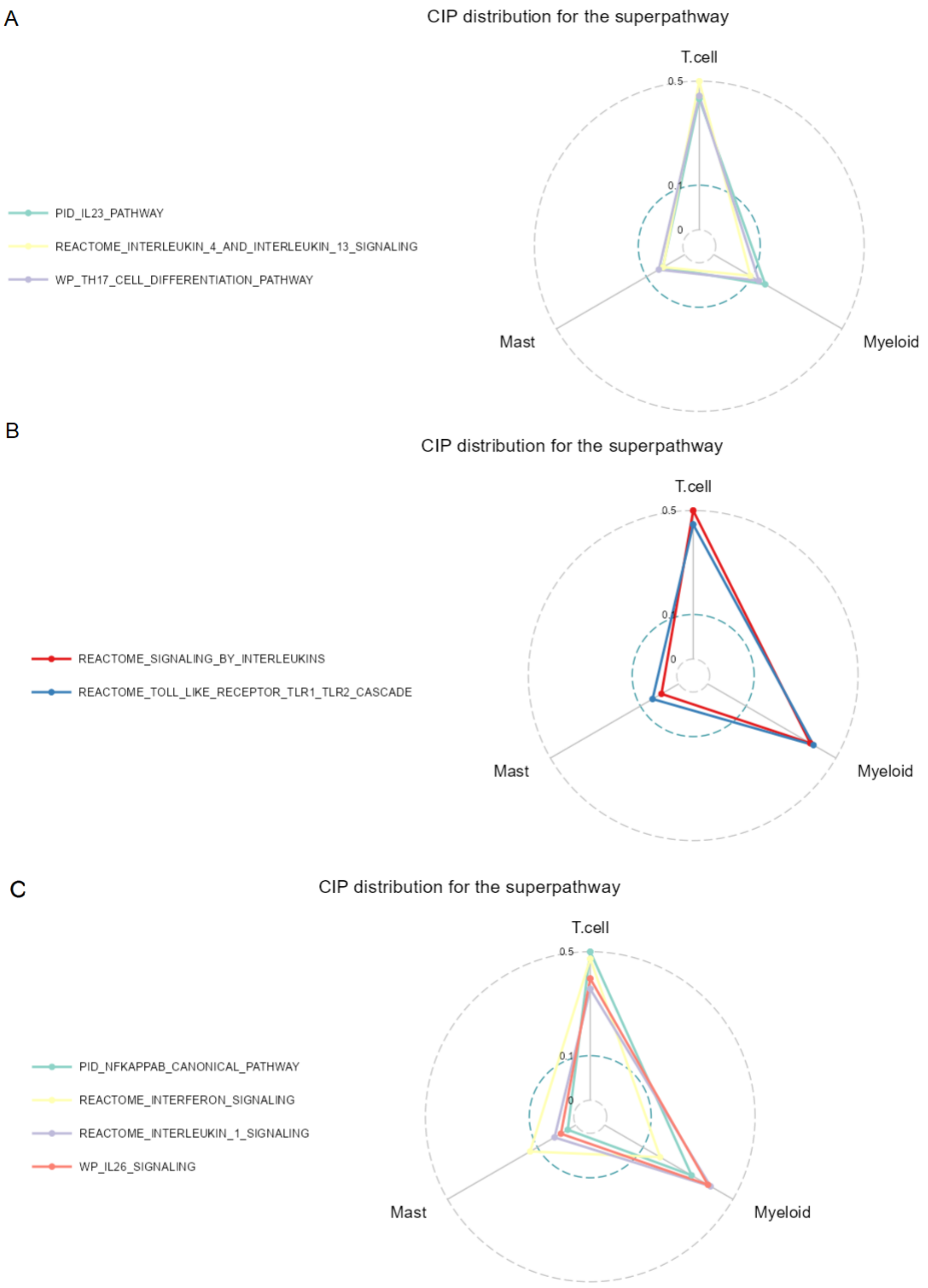

Fig H. A) HS well-recapitulated pathways with highest agreeing recapitulation values. **B)** HS well-recapitulated pathways with low agreeing recapitulation values. **C)** HS bad-recapitulated pathways with lowest agreeing recapitulation values.

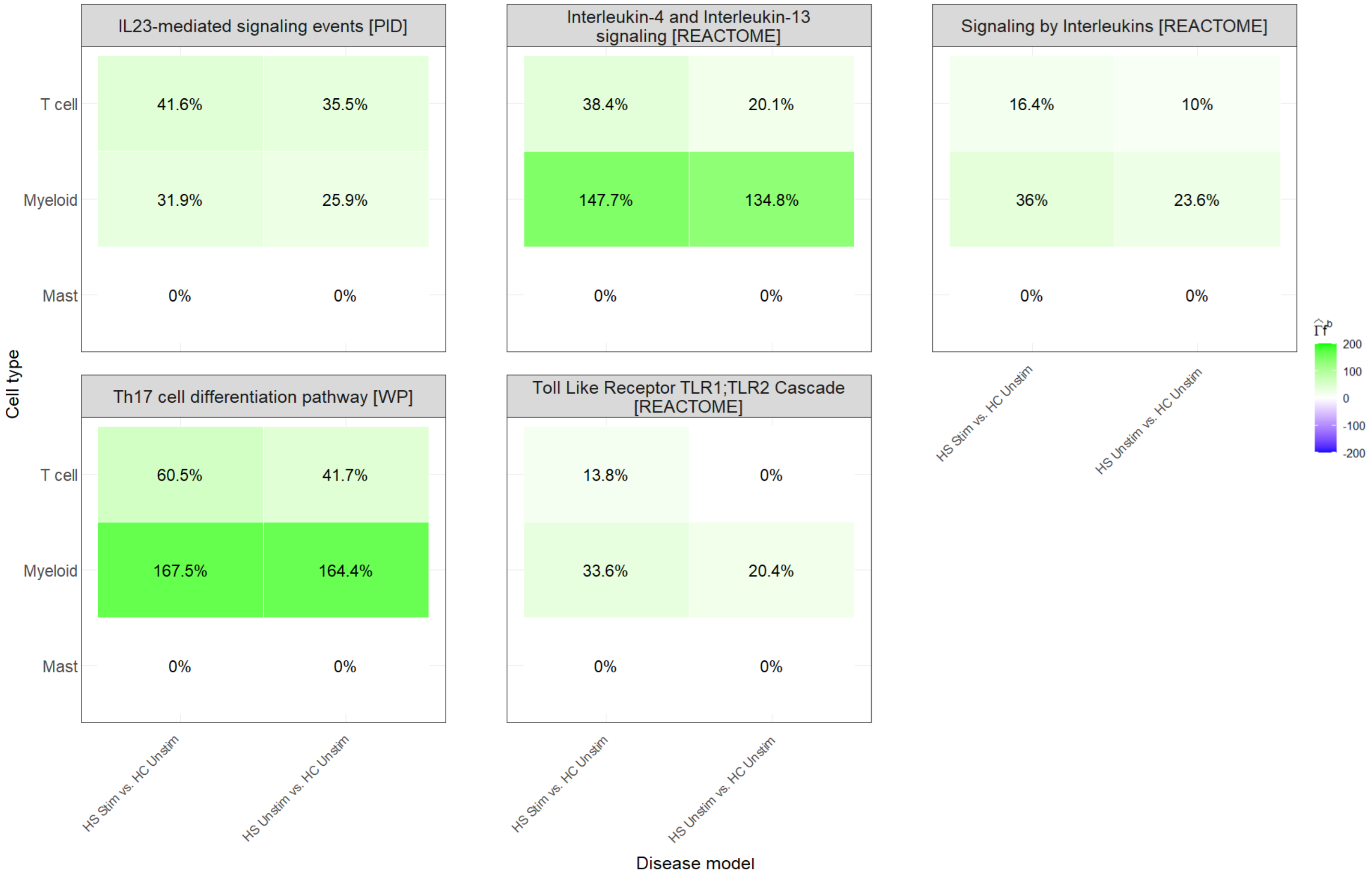

Fig I. Cell type recapitulation of well-recapitulated superpathways in HS disease models (HS Unstim vs. HC Unstim and HS Stim vs. HC Unstim).

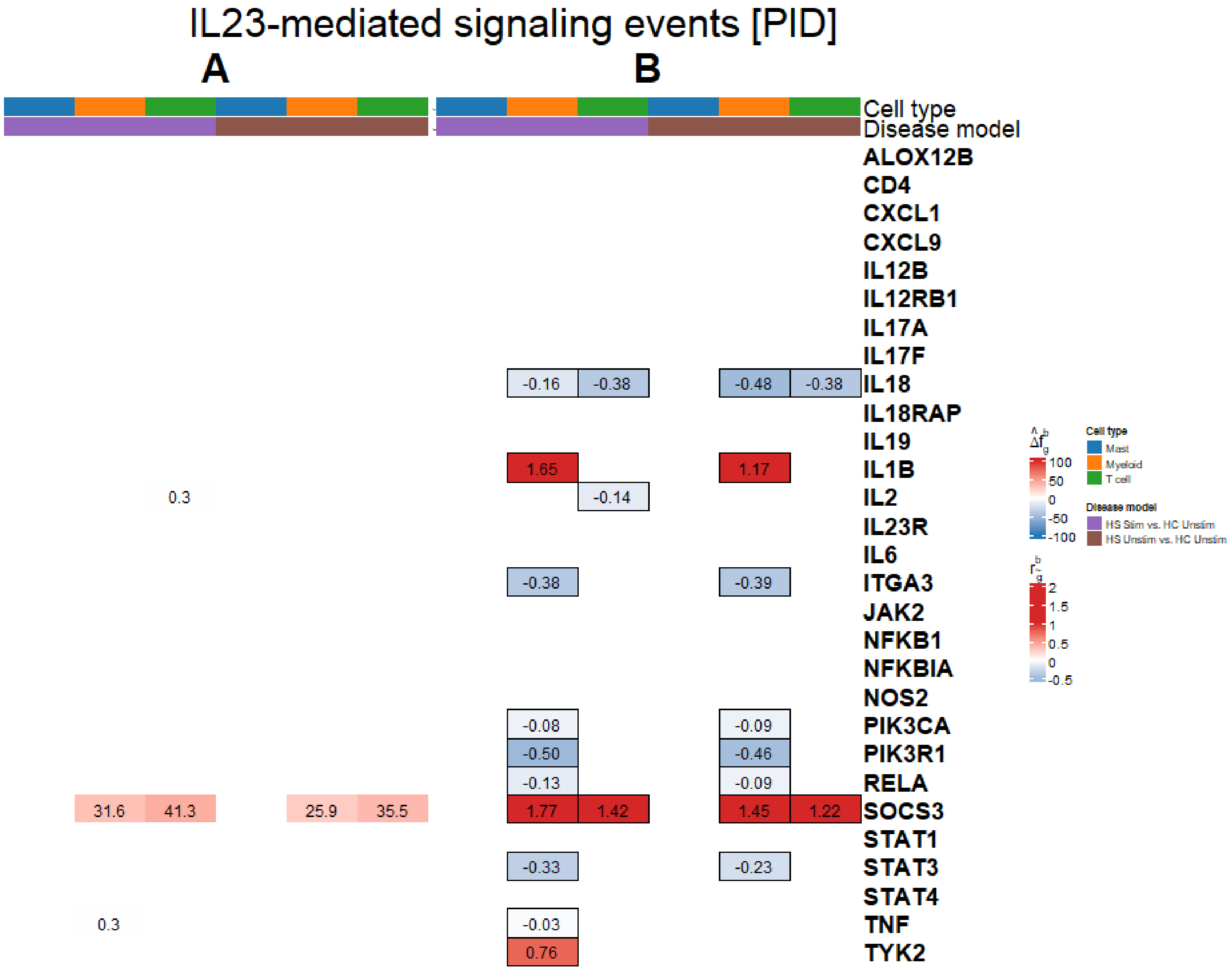
IL23-mediated signaling events [PID]
A
B
Cell type
Disease model
ALOX12B
CD4
CXCL1
CXCL9
IL12B
IL12RB1
IL17A
IL17F
-0.16 -0.38 -0.48 -0.38 IL18
IL18RAP
IL19
1.65 1.17 IL1B
0.3 -0.14 IL2
IL23R
IL6
-0.38 -0.39 ITGA3
JAK2
NFKB1
NFKBIA
NOS2
-0.08 -0.09 PIK3CA
-0.50 -0.46 PIK3R1
-0.13 -0.09 RELA
31.6 41.3 25.9 35.5 1.77 1.42 1.45 1.22 SOCS3
STAT1
-0.33 -0.23 STAT3
STAT4
0.3 -0.03 TNF
0.76 TYK2
100
50
0
-50
-100
2
1.5
1
0.5
0
-0.5
Cell type
Mast
Myeloid
T cell
Disease model
HS Stim vs. HC Unstim
HS Unstim vs. HC Unstim

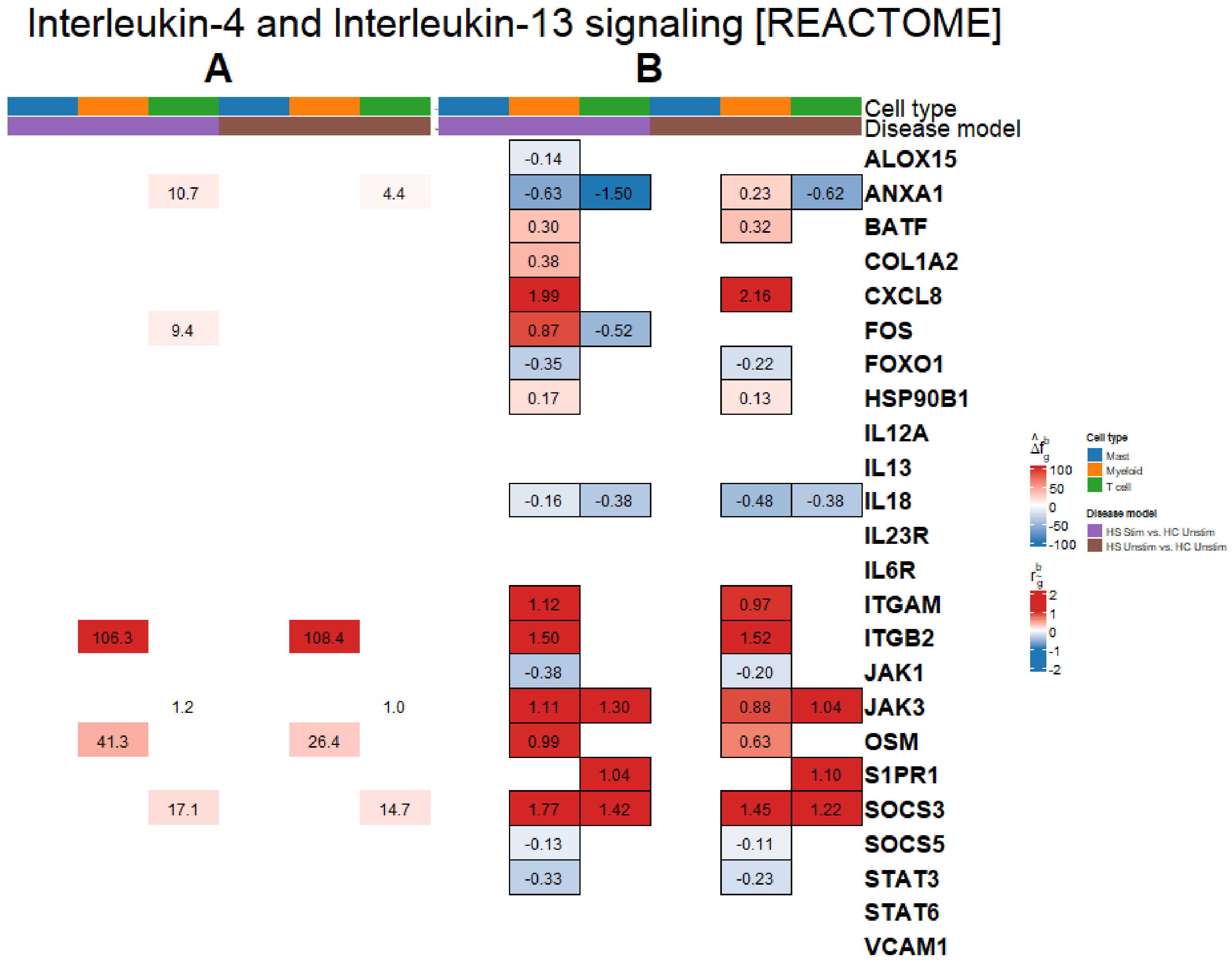
Interleukin-4 and Interleukin-13 signaling [REACTOME]
A
B
Cell type
Disease model
ALOX15
ANXA1
BATF
COL1A2
CXCL8
FOS
FOXO1
HSP90B1
IL12A
IL13
IL18
IL23R
IL6R
ITGAM
ITGB2
JAK1
JAK3
OSM
S1PR1
SOCS3
SOCS5
STAT3
STAT6
VCAM1
10.7
4.4
9.4
106.3
108.4
1.2
1.0
41.3
26.4
17.1
14.7
-0.14
-0.63
-1.50
0.23
-0.62
0.30
0.32
0.38
1.99
2.16
0.87
-0.52
-0.35
-0.22
0.17
0.13
-0.16
-0.38
-0.48
-0.38
1.12
0.97
1.50
1.52
-0.38
-0.20
1.11
1.30
0.88
1.04
0.99
0.63
1.04
1.10
1.77
1.42
1.45
1.22
-0.13
-0.11
-0.33
-0.23
100
50
0
-50
-100
2
1
0
-1
-2
Cell type
Mast
Myeloid
T cell
Disease model
HS Stim vs. HC Unstim
HS Unstim vs. HC Unstim

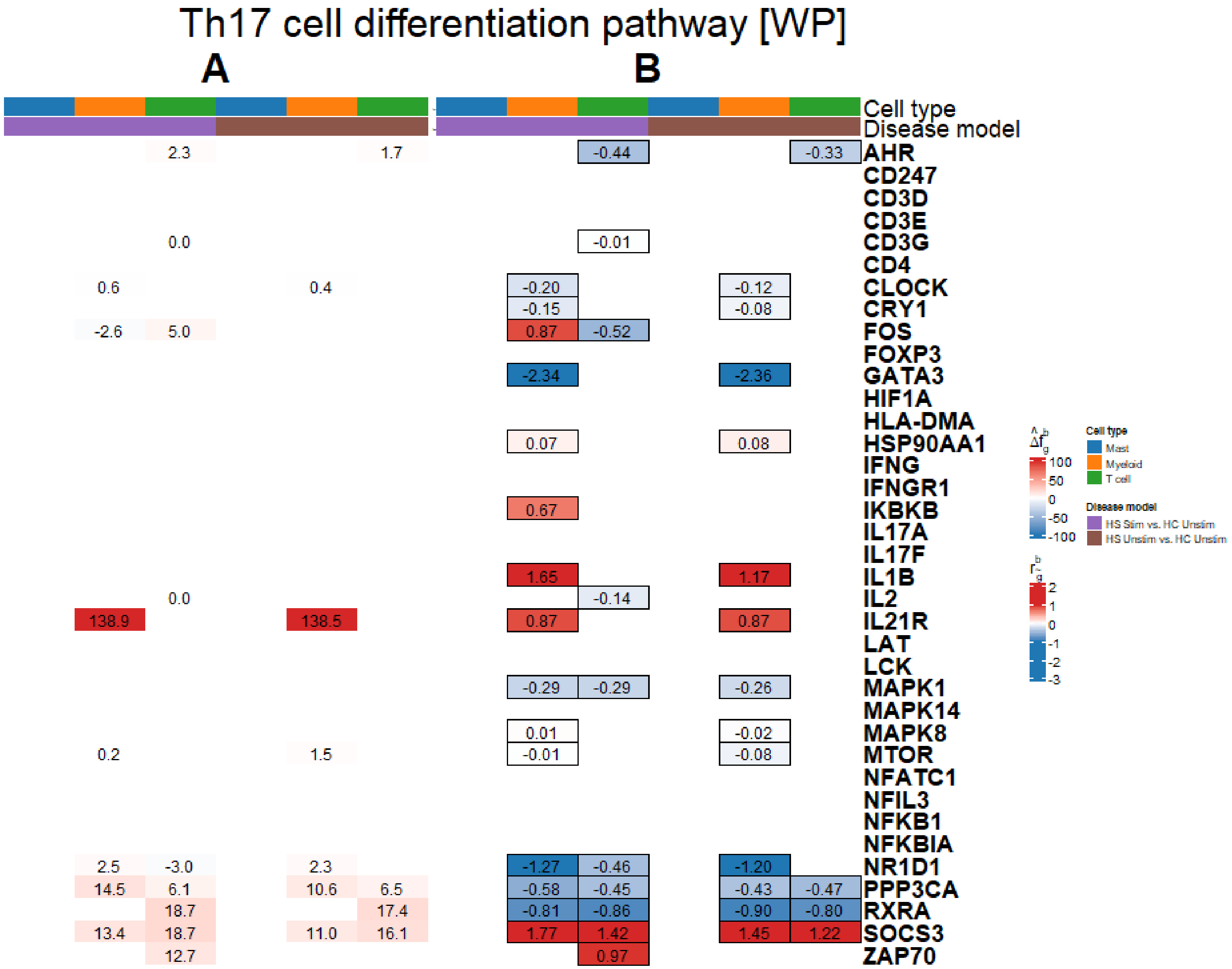
Th17 cell differentiation pathway [WP]
A
B
Cell type
Disease model
2.3 1.7 -0.44 -0.33 AHR
CD247
CD3D
CD3E
0.0 -0.01 CD3G
CD4
0.6 0.4 -0.20 -0.12 CLOCK
-0.15 -0.08 CRY1
-2.6 5.0 0.87 -0.52 FOS
FOXP3
-2.34 -2.36 GATA3
HIF1A
HLA-DMA
0.07 0.08 HSP90AA1
IFNG
IFNGR1
0.67 IKBKB
IL17A
IL17F
1.65 1.17 IL1B
0.0 -0.14 IL2
138.9 138.5 0.87 0.87 IL21R
LAT
LCK
-0.29 -0.29 -0.26 MAPK1
MAPK14
0.01 -0.02 MAPK8
0.2 1.5 -0.01 -0.08 MTOR
NFATC1
NFIL3
NFKB1
NFKBIA
2.5 -3.0 2.3 -1.27 -0.46 -1.20 NR1D1
14.5 6.1 10.6 6.5 -0.58 -0.45 -0.43 -0.47 PPP3CA
18.7 17.4 -0.81 -0.86 -0.90 -0.80 RXRA
13.4 18.7 11.0 16.1 1.77 1.42 1.45 1.22 SOCS3
12.7 0.97 ZAP70
100
50
0
-50
-100
2
1
0
-1
-2
-3
Cell type
Mast
Myeloid
T cell
Disease model
HS Stim vs. HC Unstim
HS Unstim vs. HC Unstim

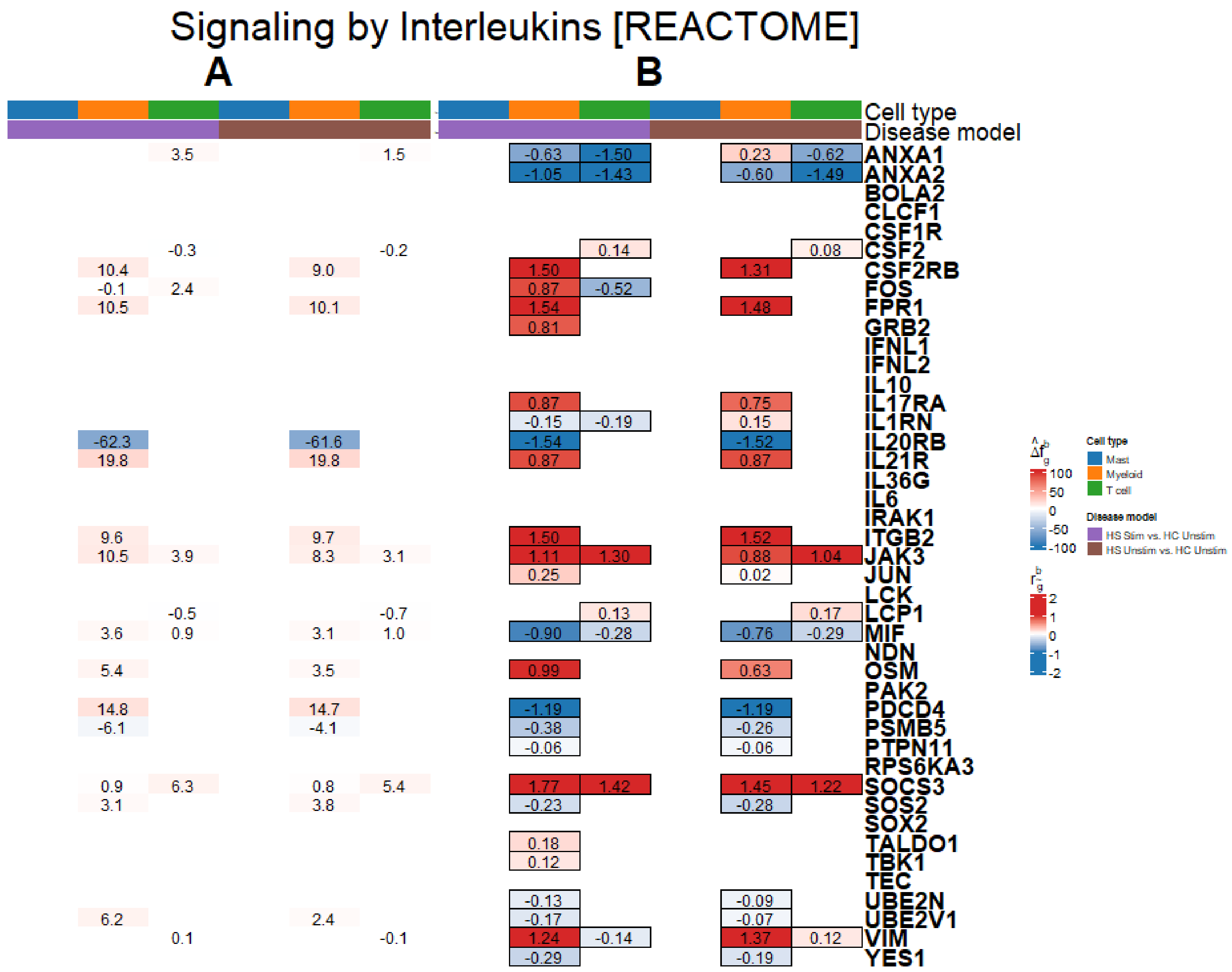

Signaling by Interleukins [REACTOME]
A
B
Cell type
Disease model
ANXA1
ANXA2
BOLA2
CLCF1
CSF1R
CSF2
CSF2RB
FOS
FPR1
GRB2
IFNL1
IFNL2
IL10
IL17RA
IL1RN
IL20RB
IL21R
IL36G
IL6
IRAK1
ITGB2
JAK3
JUN
LCK
LCP1
MIF
NDN
OSM
PAK2
PDCD4
PSMB5
PTPN11
RPS6KA3
SOCS3
SOS2
SOX2
TALDO1
TBK1
TEC
UBE2N
UBE2V1
VIM
YES1
Cell type
Mast
Myeloid
T cell
Disease model
HS Stim vs. HC Unstim
HS Unstim vs. HC Unstim

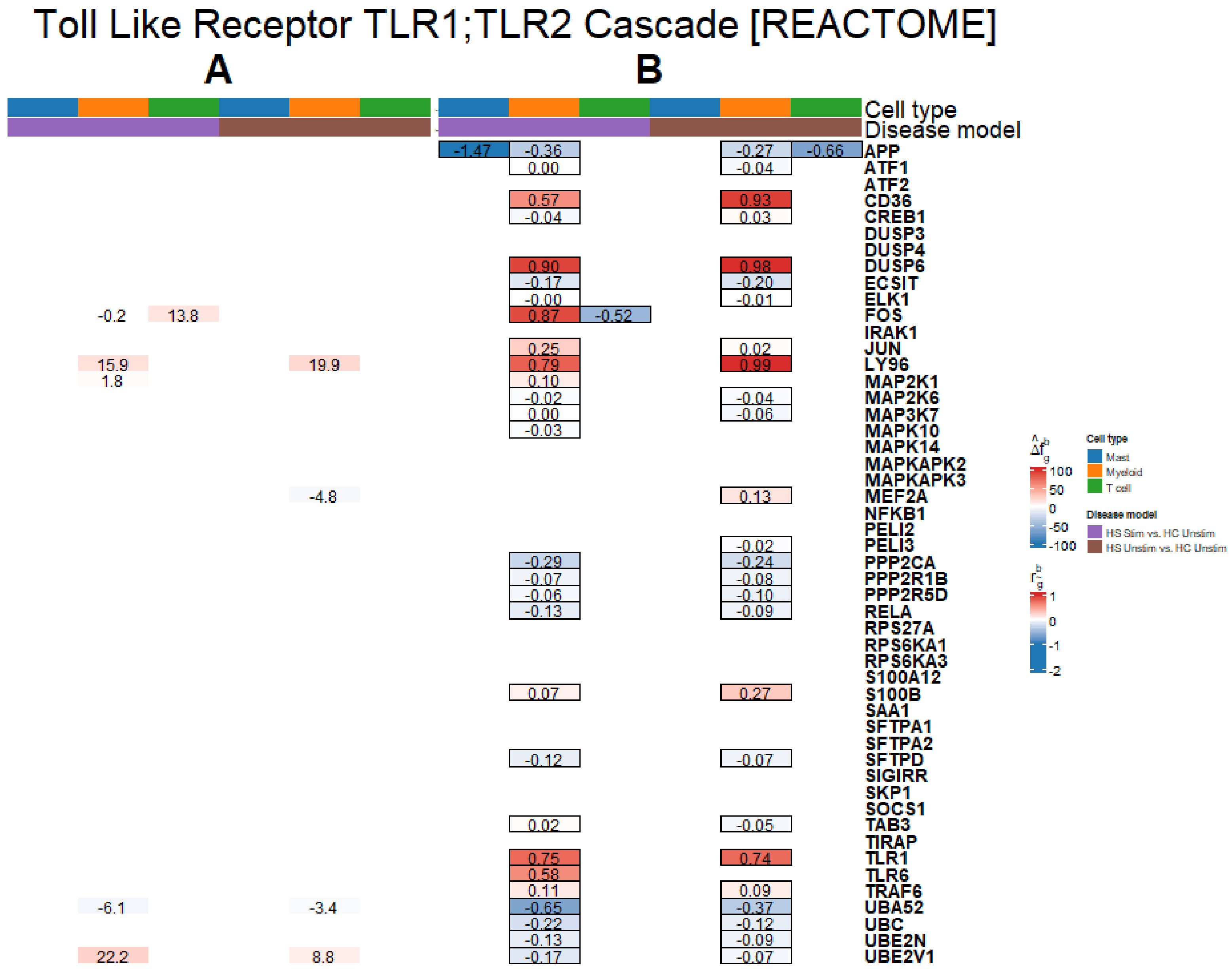

Fig J. Gene contribution and log2FC $r_{\tilde{g}}^{b}$ for well-recapitulated HS pathways. **A)** Gene contribution to cell type recapitulation by disease model. If gene set size of pathway is greater than 50, only the top 20 contributing genes, for each cell type, were displayed. Blank gene contributions correspond to 0 values. **B)** Computed $r_{\tilde{g}}^{b}$ by disease model. Framed FC refer to statistically significant $FDR \leq 0.05$ genes, as per FindMarkers. Blank FC correspond to 0 values.

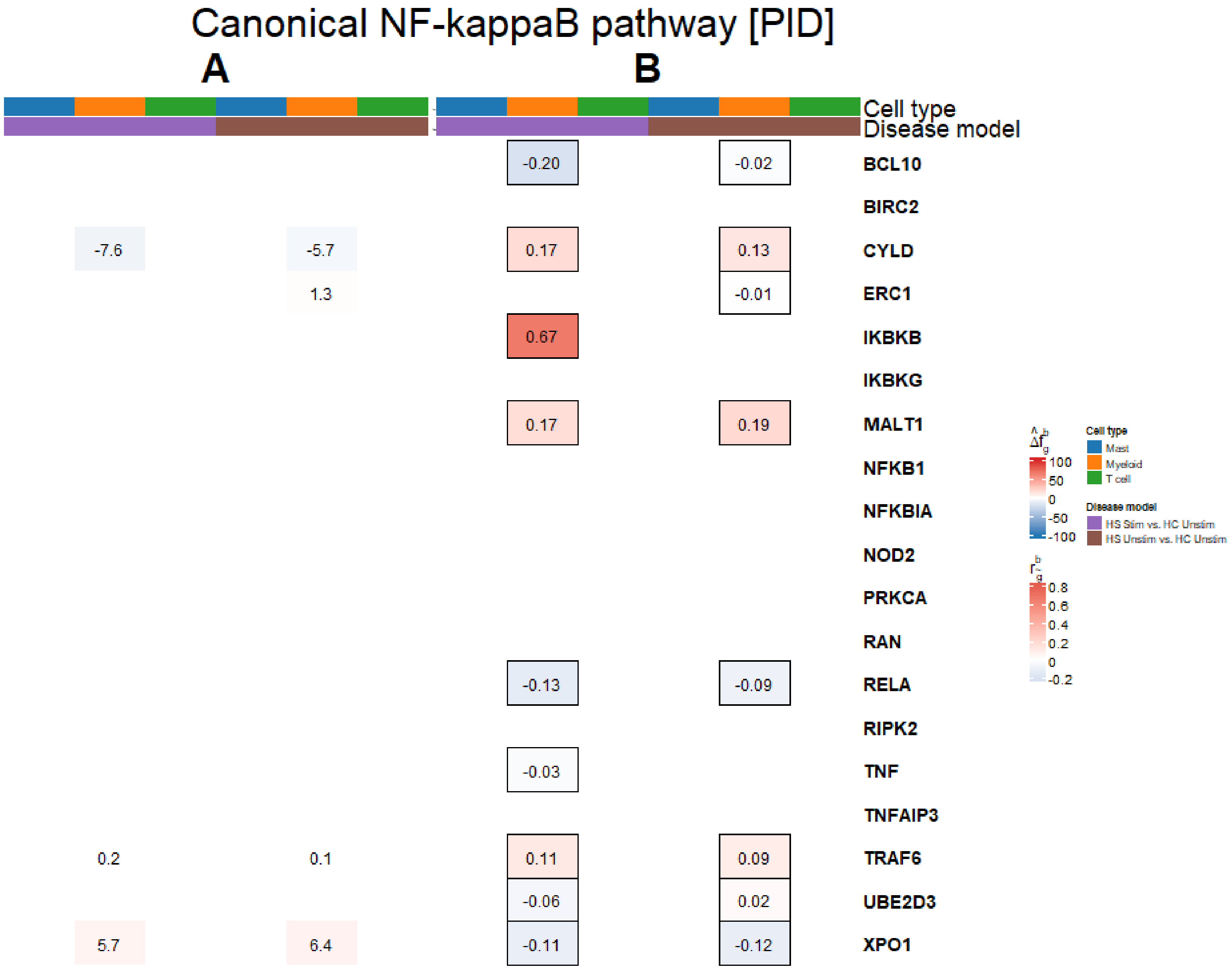
Canonical NF-kappaB pathway [PID]
A
B
Cell type
Disease model
BCL10
BIRC2
CYLD
ERC1
IKBKB
IKBKG
MALT1
NFKB1
NFKBIA
NOD2
PRKCA
RAN
RELA
RIPK2
TNF
TNFAIP3
TRAF6
UBE2D3
XPO1
-0.20
-0.02
-7.6
-5.7
0.17
0.13
1.3
-0.01
0.67
0.17
0.19
-0.13
-0.09
-0.03
0.2
0.1
0.11
0.09
-0.06
0.02
5.7
6.4
-0.11
-0.12
100
50
0
-50
-100
0.8
0.6
0.4
0.2
0
-0.2
Cell type
Mast
Myeloid
T cell
Disease model
HS Stim vs. HC Unstim
HS Unstim vs. HC Unstim

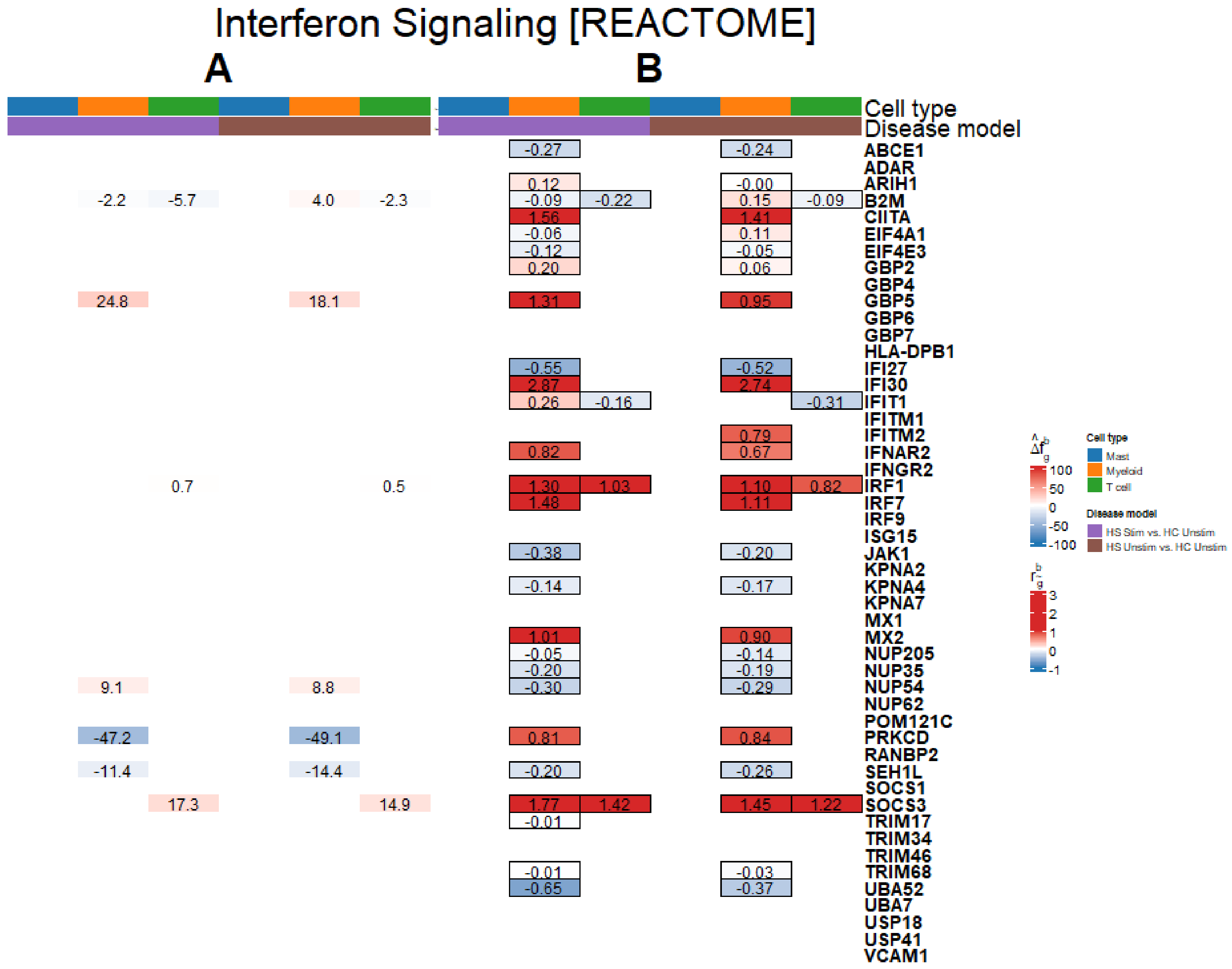
Interferon Signaling [REACTOME]
A
B
Cell type
Disease model
ABCE1
ADAR
ARIH1
B2M
CIITA
EIF4A1
EIF4E3
GBP2
GBP4
GBP5
GBP6
GBP7
HLA-DPB1
IFI27
IFI30
IFIT1
IFITM1
IFITM2
IFNAR2
IFNGR2
IRF1
IRF7
IRF9
ISG15
JAK1
KPNA2
KPNA4
KPNA7
MX1
MX2
NUP205
NUP35
NUP54
NUP62
POM121C
PRKCD
RANBP2
SEH1L
SOCS1
SOCS3
TRIM17
TRIM34
TRIM46
TRIM68
UBA52
UBA7
USP18
USP41
VCAM1
Cell type
Mast
Myeloid
T cell
Disease model
HS Stim vs. HC Unstim
HS Unstim vs. HC Unstim

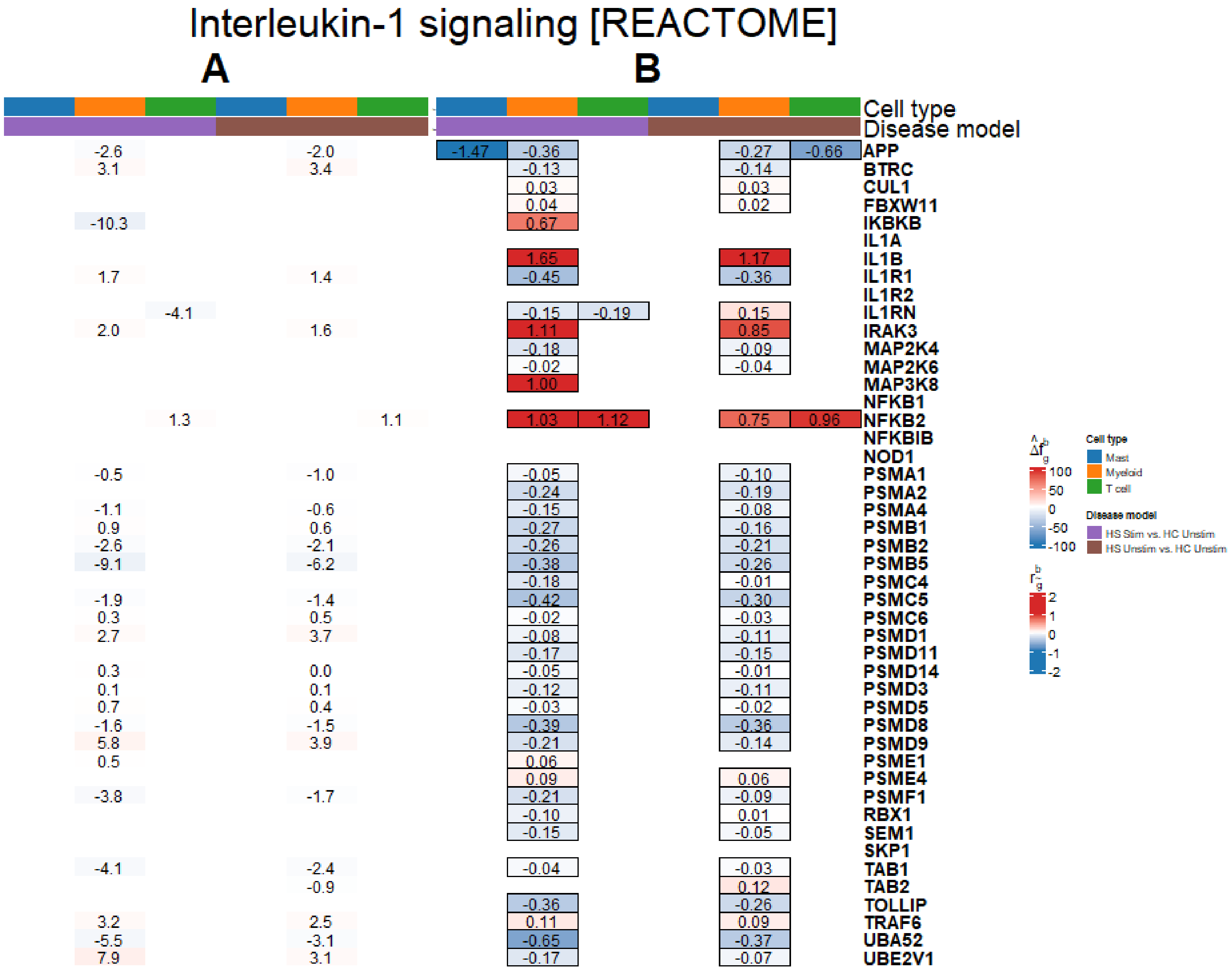
Interleukin-1 signaling [REACTOME]
A
B
Cell type
Disease model
APP
BTRC
CUL1
FBXW11
IKBKB
IL1A
IL1B
IL1R1
IL1R2
IL1RN
IRAK3
MAP2K4
MAP2K6
MAP3K8
NFKB1
NFKB2
NFKBIB
NOD1
PSMA1
PSMA2
PSMA4
PSMB1
PSMB2
PSMB5
PSMC4
PSMC5
PSMC6
PSMD1
PSMD11
PSMD14
PSMD3
PSMD5
PSMD8
PSMD9
PSME1
PSME4
PSMF1
RBX1
SEM1
SKP1
TAB1
TAB2
TOLLIP
TRAF6
UBA52
UBE2V1
Cell type
Mast
Myeloid
T cell
Disease model
HS Stim vs. HC Unstim
HS Unstim vs. HC Unstim
100
50
0
-50
-100
2
1
0
-1
-2

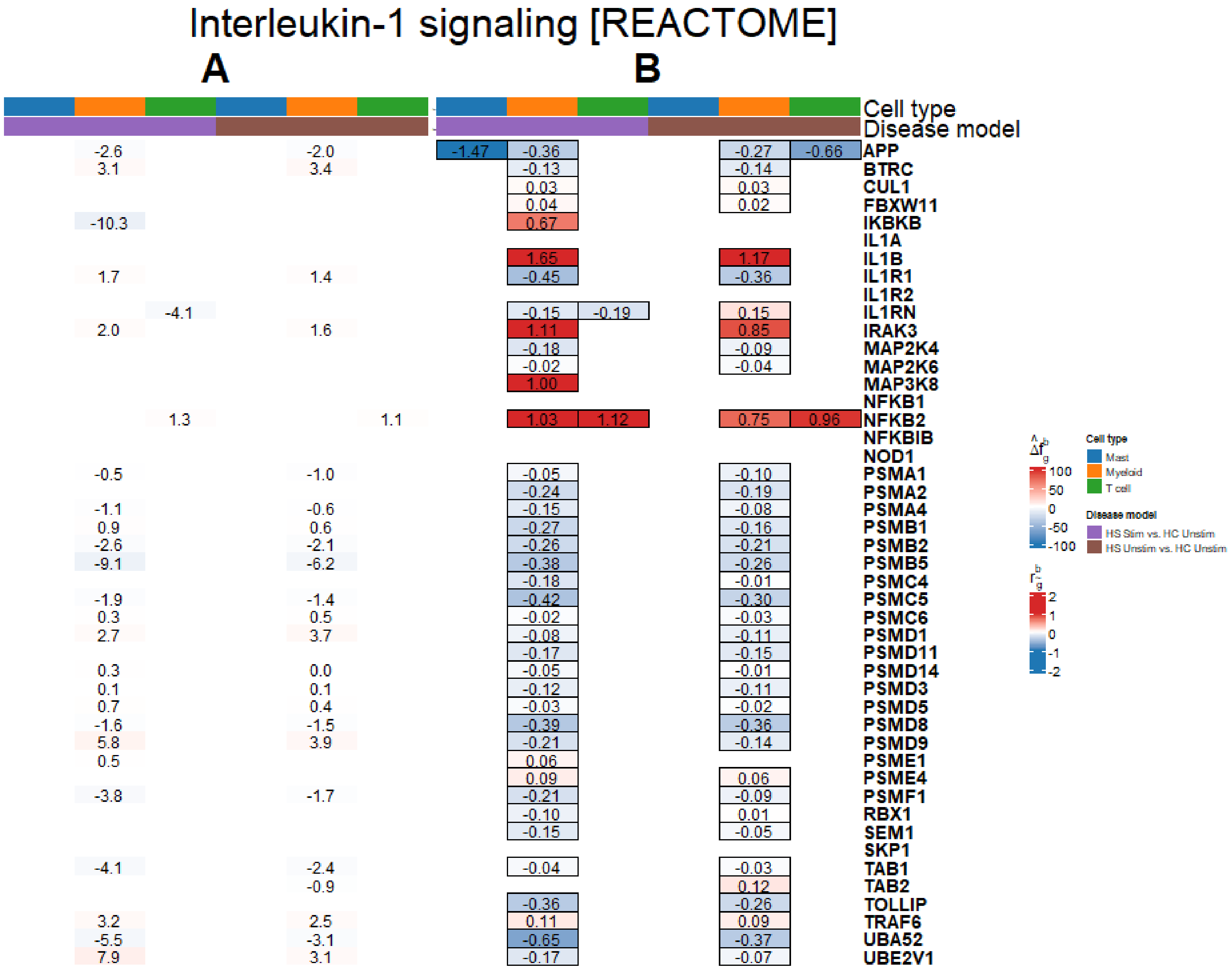
Interleukin-1 signaling [REACTOME]
A
B
Cell type
Disease model
APP
BTRC
CUL1
FBXW11
IKBKB
IL1A
IL1B
IL1R1
IL1R2
IL1RN
IRAK3
MAP2K4
MAP2K6
MAP3K8
NFKB1
NFKB2
NFKBIB
NOD1
PSMA1
PSMA2
PSMA4
PSMB1
PSMB2
PSMB5
PSMC4
PSMC5
PSMC6
PSMD1
PSMD11
PSMD14
PSMD3
PSMD5
PSMD8
PSMD9
PSME1
PSME4
PSMF1
RBX1
SEM1
SKP1
TAB1
TAB2
TOLLIP
TRAF6
UBA52
UBE2V1
Cell type
Mast
Myeloid
T cell
Disease model
HS Stim vs. HC Unstim
HS Unstim vs. HC Unstim

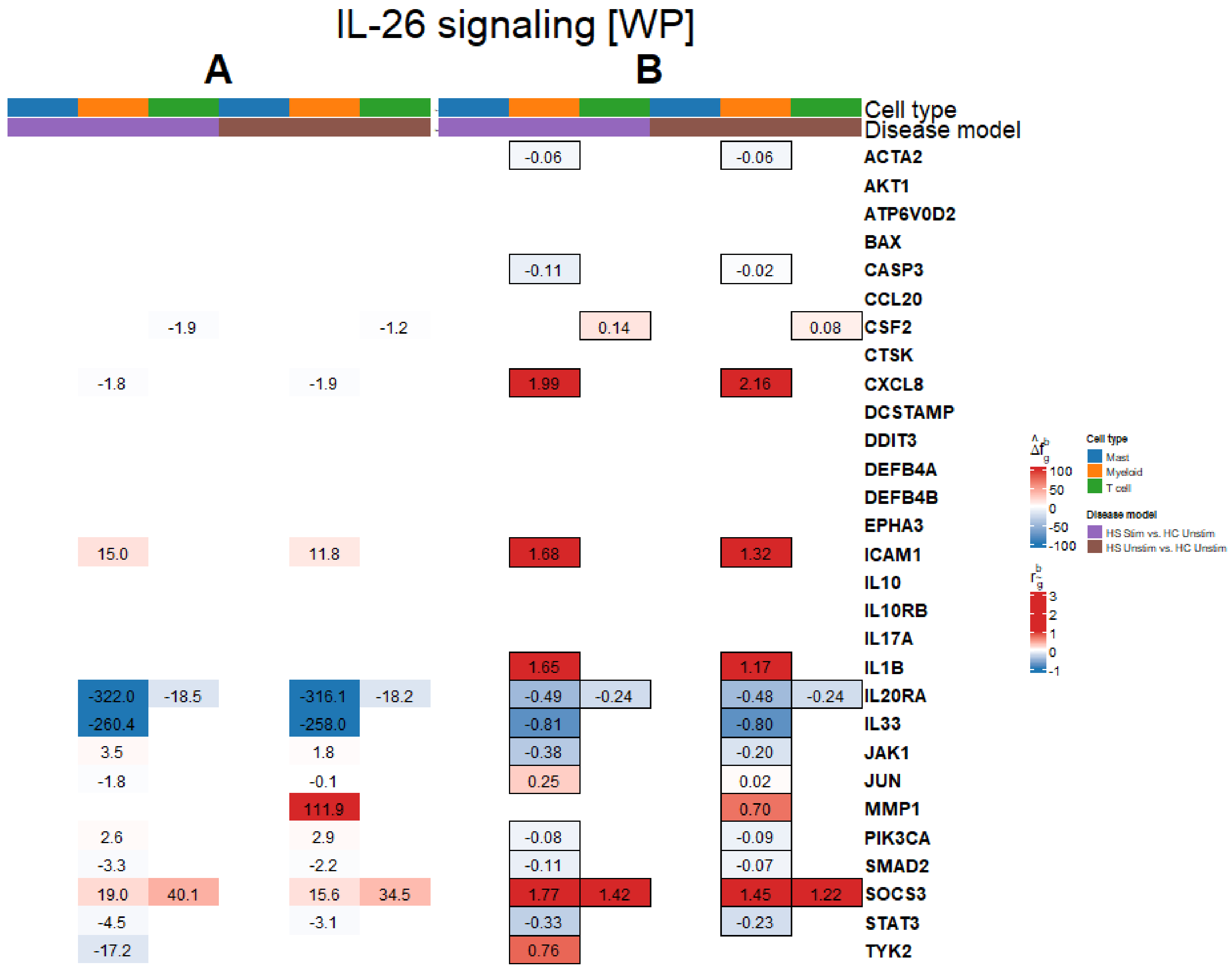

Fig K. Gene contribution and log2FC $r^b_{\tilde{g}}$ for bad-recapitulated HS pathways. **A)** Gene contribution to cell type recapitulation by disease model. If gene set size of pathway is greater than 50, only the top 20 contributing genes, for each cell type, were displayed. Blank gene contributions correspond to 0 values. **B)** Computed $r^b_{\tilde{g}}$ by disease model. Framed FC refer to statistically significant $FDR \leq 0.05$ genes, as per FindMarkers. Blank FC correspond to 0 values.

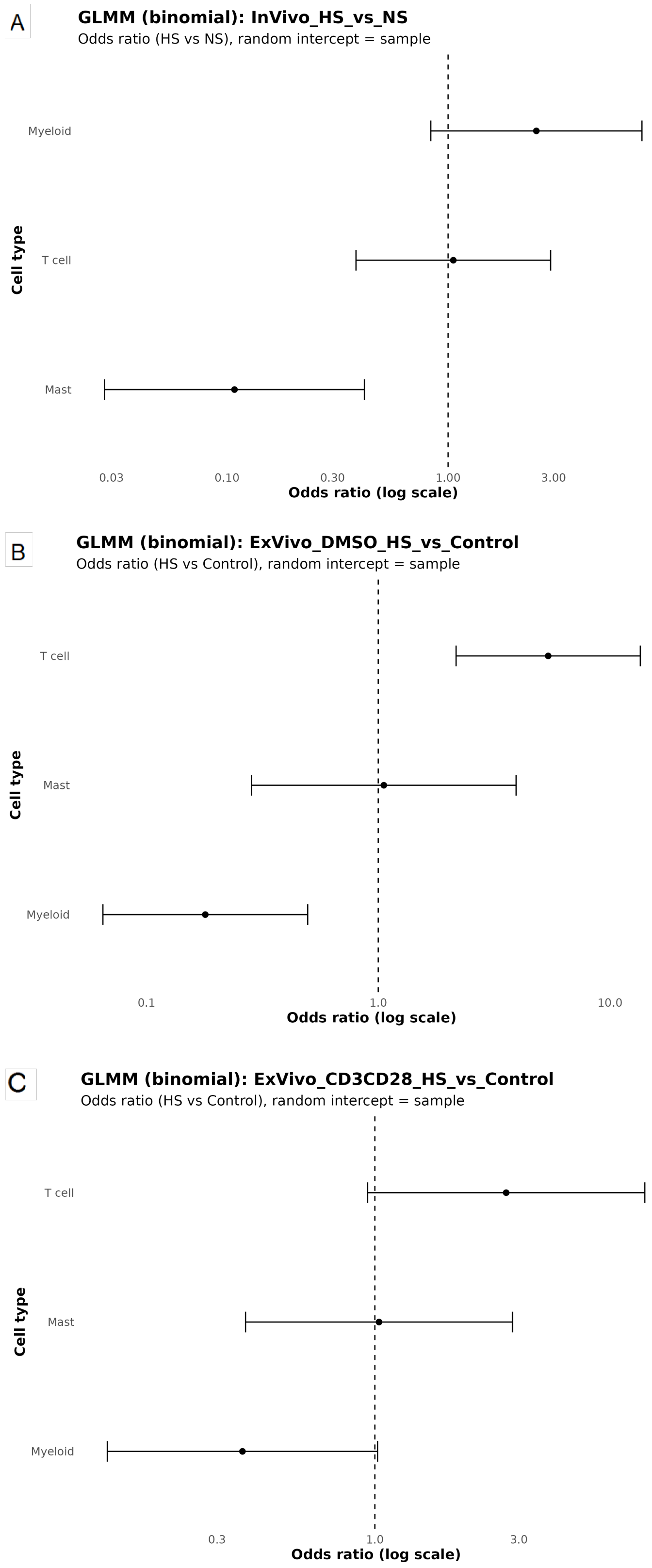

Fig L. Cell type composition in HS in vivo and HS explants. A) Generalized linear mixed model (binomial) for in vivo HS vs. Normal subject (NS) showing odds ratios (HS vs. NS) by cell type, with sample as random intercept. **B)** Same model for ex vivo HS explants (DMSO, unstimulated) vs. Healthy control skin (DMSO, unstimulated). **C)** Same model for ex vivo HS explants stimulated with CD3/CD28 vs. Healthy control skin stimulated with CD3/CD28. Points indicate estimated odds ratios on the log scale; horizontal bars show 95% confidence intervals; the vertical dashed line marks an odds ratio of 1 (no difference in abundance).
