# Supplementary material for: singIST: An integrative method for comparative single-cell transcriptomics between disease models and humans
Source: PLoS Comput Biol. 2026 Mar 16;22(3):e1014002. doi: 10.1371/journal.pcbi.1014002 (PMC13008255; doi:10.1371/journal.pcbi.1014002)
Supplement: S4 File — Overview of the cell type harmonization between human data and mouse models, including cluster selection and mapping criteria. (PDF) [file pcbi.1014002.s004.pdf]

Organism	Cluster	Cell type granularity 1**	Cell type granularity 2***	Action	Reason
*Homo Sapiens Sapiens**	KC-1	Keratinocytes	Suprabasal Keratinocyte	KEEP GRANULARITY 1	No relevant information reported on granularity 2 Keratinocytes in the original paper, only for granularity 1 Keratinocyte
	KC-2		Basal Keratinocyte		
	KC-3		Late differentiation Keratinocyte		
	KC-4		Proliferating Keratinocyte		
	KC-5		?		
	Tregs	T-cell	T-regs	KEEP GRANULARITY 2	
	TC-1	T-cell	Tissue Resident Memory T-cell		
	TC-2	T-cell	CD8+ effector T-cell		
	TC-3	T-cell	(CD161)+ T-cell / Th2a ?	DROP CLUSTER	- Small cluster according to [1] "*The smaller clusters TC-3, TC-4, and TC-5 were either absent (TC-3) or only detectable in small numbers (TC-4 and TC-5) in healthy control samples* "<br>- Not clear from the publication if its (CD161)+ T-cell or Th2A
	TC-4	T-cell	?	DROP CLUSTER	Non-identified cell type
	TC-5	T-cell	Proliferating T-cell	DROP CLUSTER	Small cluster according to [1] "*The smaller clusters TC-3, TC-4, and TC-5 were either absent (TC-3) or only detectable in small numbers (TC-4 and TC-5) in healthy control samples* "
	TC-6	T-cell	Natural Killer T-cell	KEEP GRANULARITY 2	
	Melanocytes	Melanocytes		KEEP GRANULARITY 1	It's the only granularity reported
	LC	Dendritic cells	Langerhans cells	KEEP GRANULARITY 2	
	DC-1	Dendritic cells	Myeloid cells		
	DC-2	Dendritic cells	Mature DCs	DROP CLUSTER	- Not relevant according to [1] "*Although the list of differentially expressed genes in DC-2 was relatively short, likely due to very small cell numbers and thus lacking statistical power, a few anti-inflammatory genes showed marked up-regulation during treatment, especially,...* "<br>- We are not considering treatment period, only HC and Baseline.<br>- Very small cluster to consider it for analysis
	DC-3	Dendritic cells	Plasmacytoid DCs	DROP CLUSTER	- Very small population according to [1] "*We found a very small population of plasmacytoid DCs (DC-3)...* "
	MastC_Others	?		DROP CLUSTER	Non-identified cell type

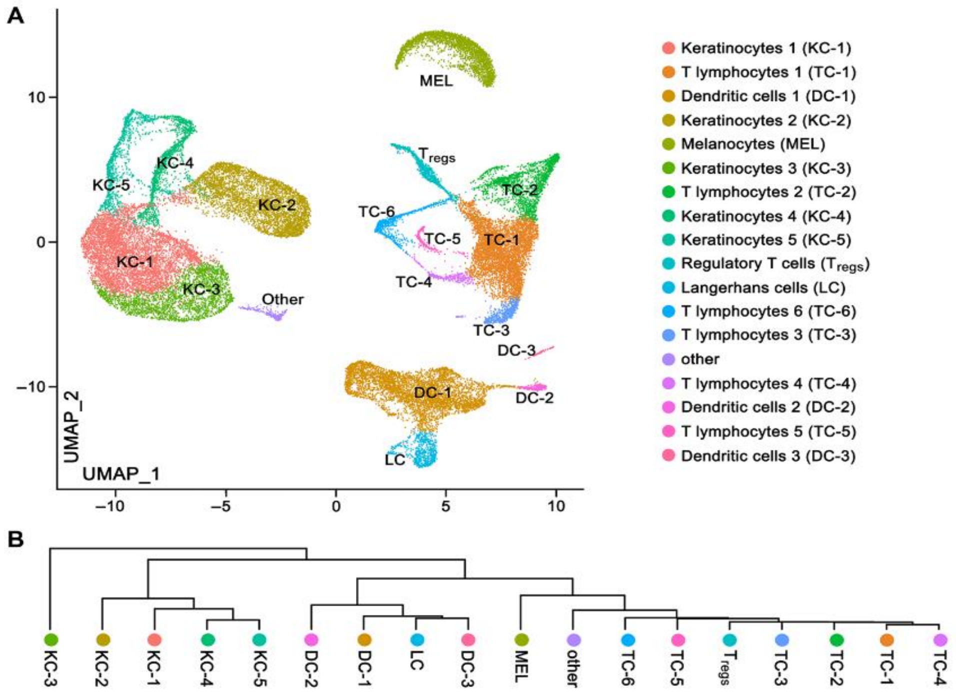

*Human data extracted from [1] "*Persistence of mature dendritic cells, TH2A, and Tc2 cells characterize clinically resolved atopic dermatitis under IL-4Ralpha blockade* " url: https://www.science.org/doi/epdf/10.1126/sciimmunol.abe2749

** If "?" cell type granularity 1 is not clearly identified in the publication

*** If "?" cell type granularity 2 is not clearly identified in the publication

Organism	Disease model(s)	Cluster	Cell type granularity 1*	Cell type granularity 2*	Action	Reason
Mus musculus	*Imiquimod (5%)*<br>and<br>*Oxazolona 3 Challenges*	0	Mac (Macrophages)			
		1	DETC (Dendritic Epidermal T-cells)			
		2	M/MdM (Monocyte derived Macrophages)			
		3	dgammadeltaT			
		4	cDC2 (Type 2 conventional Dendritic cell)			
		5	ILC2 (Type 2 Innate Lymphoid cell)			
		6	Thet (T-cell heterogeneous)	Treg<br>Tconv (Conventional T-cell)<br>CD8+ (CD8+ effector T-cell)<br>DNT (Double Negative T-cell)		
		7	Fibroblasts1			
		8	Indeterminate1			
		9	M/B (Mast cells and Basophils)			
		10	Neu (Neutrophils)			
		11	Cell cycle1			
		12	Keratinocytes			
		13	Cell cycle2			
		14	cDC1 (Type 1 conventional Dendritic cell)			
		15	LC (Langerhans cell)			
		16	mDCs (Mature Dendritic cell)			
		17	NK (Natural Killer cell)			
		18	Endothelial			
		19	Indeterminate2			
		20	Fibroblasts2			

*IMQ-OXA data extracted from [2] "Single-Cell Profiling Reveals Divergent, Globally Patterned Immune Responses in Murine Skin Inflammation" url: https://pubmed.ncbi.nlm.nih.gov/33205009/

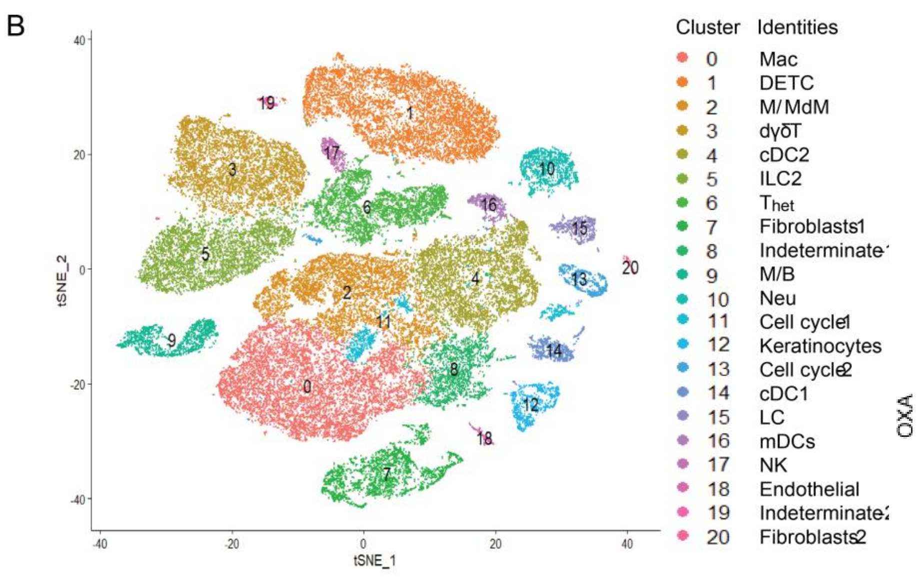

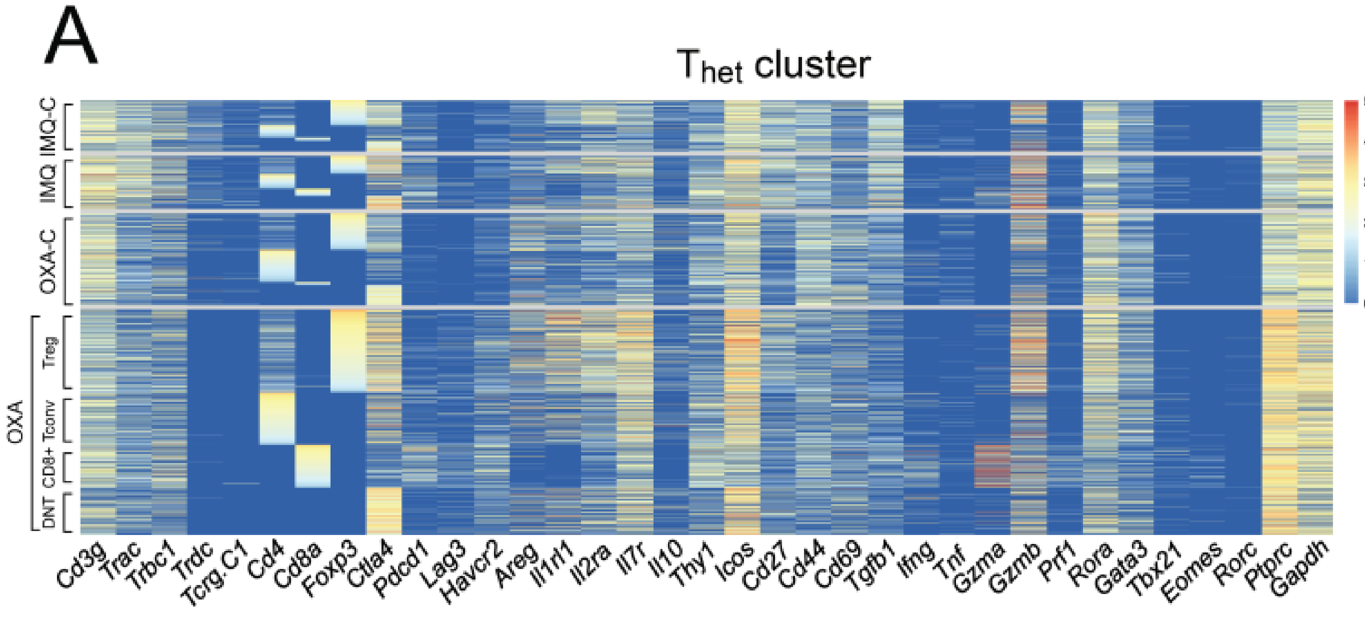

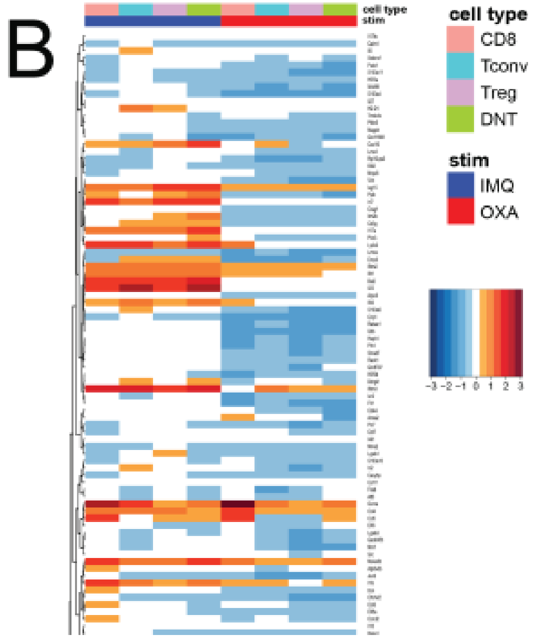

Human cells	IMQ-OXA cluster	IMQ-OXA cells	Reason
Keratinocytes	12	Keratinocytes	
Tregs	6	T-heterogeneous (Treg)	
Tissue Resident Memory T-cell	**?**	**?**	
CD8+ effector T-cell	6	T-heterogeneous (CD8+ effector T-cell)	
Natural Killer T-cell	17	NK	
Melanocytes		**None**	Melanocytes are not present in murine ear skin but either bulge and bulb regions of hair follicles, tail or ventral paws of non-hairy skin according to [3] "*Firstly, mouse pelage skin interfollicular epidermis entirely lacks functional, pigment-producing melanocytes. While murine melanocytes are found either in the bulge and bulb regions of hair follicles, in the tail, or in the ventral paws of non-hairy mouse skin, functional human melanocytes are mostly located in the basal layer of the epidermis (Gola et al., 2012).* "
Langerhans cells	15	LC (Langerhans cells)	
Myeloid cells	0, 2, 9, 10, 17	Mac (Macrophages), M/MdM (Monocyte derived Macrophages), M/B (Mast cells/Basophils), Neu (Neutrophils)	

[3] "*Characterization of a melanocyte progenitor population in human interfollicular epidermis* ". Cell Reports.
