# Supplementary material for: singIST: An integrative method for comparative single-cell transcriptomics between disease models and humans
Source: PLoS Comput Biol. 2026 Mar 16;22(3):e1014002. doi: 10.1371/journal.pcbi.1014002 (PMC13008255; doi:10.1371/journal.pcbi.1014002)
Supplement: S5 File — Simulation studies to validate singIST recapitulation properties and benchmark against ODEGs. (PDF) [file pcbi.1014002.s005.pdf]

Supplementary Material S5: Simulation study

1. PROVING SINGIST RECAPITULATION PROPERTIES THROUGH SIMULATION

A. Simulation methodology

There are two main inputs we will simulate: human pseudobulk expression and the disease model $\log_2 FC$. Once these inputs are simulated, we use the R/singIST library to run singIST workflow based on those inputs.

A.1. Human scRNAseq pseudobulk input

We simulate the human pseudobulk expression for p genes across B cell-type blocks in two conditions ("case" vs. "control") under typical single-cell constraints ($p \gg n$, high feature correlation, small sample size). Our procedure is:

Gene sampling. Let $\mathcal{G} = \{g_1, \ldots, g_p\}$ be a random sample of p real HGNC symbols. We assume same number of genes per block to simplify the simulation.

Block covariance. Let $\mathcal{C} = \{c_1, \ldots, c_B\}$ denote the B cell-types (e.g T cells, B cells, ...). We simulate each block independently but with the same covariance structure. For each block, $c = 1, \ldots, B$, define a correlation matrix $\Sigma \in \mathbb{R}^{p \times p}$ by

$$\Sigma_{ij} = \begin{cases} 1, & i = j, \\ \rho_{ij},\ \rho_{ij} \sim \text{Uniform}(0.6, 0.9), & i \neq j, \end{cases} \tag{S1}$$

and symmetrize $\Sigma \leftarrow (\Sigma + \Sigma^\top)/2$ and then projected to the nearest positive–definite correlation matrix via `Matrix::nearPD`.

Latent log-expression sampling. Let $n_{\text{case}} = 5$ and $n_{\text{ctrl}} = 5$ to reflect typical pseudobulk replica counts. For a per-cell-type and per-gene effect vector $\boldsymbol{\mu}^{\boldsymbol{c}} = (\mu^c_{g_1}, \ldots, \mu^c_{g_p})$ we stack $\boldsymbol{\mu} = (\boldsymbol{\mu}^{\boldsymbol{c_1}}, \ldots, \boldsymbol{\mu}^{\boldsymbol{c_B}})$. To simplify simulation we set $\boldsymbol{\mu_{ctrl}} = \boldsymbol{\mu^{(c)}_{ctrl}} = 10000$ constant for all cell-types and genes, and we let $\boldsymbol{\mu_{case}}$ as an input parameter. We draw i.i.d samples from:

$$Z^{(c)}_{\text{ctrl}} \sim \mathcal{N}_p(\boldsymbol{\mu^{(c)}_{ctrl}}, \Sigma), \quad Z^{(c)}_{\text{case}} \sim \mathcal{N}_p(\boldsymbol{\mu^{(c)}_{case}}, \Sigma), \tag{S2}$$

We stack to form $Z^{(c)} \in \mathbb{R}^{(n_{case}+n_{ctrl}) \times p}$

Log-normalization. To mimic $\log_2$(CPM+1) pseudobulk, we first truncate negative values to 0 and we set:

$$X^{(c)}_{ij} \leftarrow 0, \quad X^{(c)}_{ij} < 0 \tag{S3}$$

$$X^{(c)}_{ij} = \log_2 \left(X^{(c)}_{ij} + 1 \right) \tag{S4}$$

and then

truncation ensures a non-negative expression. Finally, concatenate all blocks to obtain the pseudobulk log-normalized gene expression matrix input:

$$X = \begin{bmatrix} X^{(c_1)} \\ X^{(c_2)} \\ \vdots \\ X^{(c_B)} \end{bmatrix} \in \mathbb{R}^{B(n_{\text{case}}+n_{\text{ctrl}}) \times p} \tag{S5}$$

Where i denotes a combination of cell-type per sample and j a simulated HGNC gene name.

A.2. Disease model $\log_2 FC$ input

For downstream recapitulation, we construct a list of per-cell-type differential-expression tables. Let $\mathcal{G}$ denote the same p genes.

Effect magnitude. Choose a fixed interval $[a, b] \subset \mathbb{R}$; for each cell-type c and gene g, draw

$$\log_2 \mathrm{FC}_{c,g} \sim \mathrm{Uniform}(a, b), \tag{S6}$$

Statistical signficance. Let $\pi \in [0, 1]$ be the desired proportion of "significant" genes. We sample an index set $\mathcal{S} \subset \{1, \dots, p\}$ with $|\mathcal{S}| = \lfloor \pi p \rfloor$. Then define adjusted p-values:

$$p_{c,g}^{(\mathrm{adj})} = \begin{cases} \mathrm{Uniform}(0,\ 0.05), & i \in \mathcal{S}, \\ \mathrm{Uniform}(0.05,\ 1), & i \notin \mathcal{S}, \end{cases} \tag{S7}$$

Auxiliary columns. Some auxiliary variables needed for the input, but whose value do not affect recapitulations as they're just informative values, are drawn:

$$p_{c,g}, \mathrm{pct1}_{c,g}, \mathrm{pct2}_{\ c,g} \sim \mathrm{Uniform}(0, 1) \tag{S8}$$

Assemble list of data frames. We define a list of data frames, as required by singIST when user introduces a predefined list of $\log_2 FC$, as:

$$\boldsymbol{D_c} = \begin{bmatrix} p_{c,1} & \log_2 \mathrm{FC}_{c,1} & \mathrm{pct1}_{c,1} & \mathrm{pct2}_{c,1} & p_{c,1}^{(\mathrm{adj})} \\ \vdots & \vdots & \vdots & \vdots & \vdots \\ p_{c,p} & \log_2 \mathrm{FC}_{c,p} & \mathrm{pct1}_{c,p} & \mathrm{pct2}_{c,p} & p_{c,p}^{(\mathrm{adj})} \end{bmatrix} \tag{S9}$$

Collected into a named list $\mathcal{L} = \{\boldsymbol{D_{c_1}}, \dots, \boldsymbol{D_{c_B}}\}$

B. Simulation results

B.1. Sign and magnitude of recapitulation

Here we aim to prove the claims of the interpretation of recapitulation sign and magnitude as provided in the "Interpretation of recapitulation measures" section of the manuscript. The recapitulation value should be negative wherever the changes in the disease model, measured as $\log_2 FC$, are opposed to the human disease changes. That is, if human expression change is up for the target class against base class but $\log_2 FC$ is negative and viceversa, then the recapitulation should be negative. However, positive recapitulation values should arise whenever there's agreement in direction change between both. The magnitude of the recapitulation should also increase/decrease as $\log_2 FC$ increases/decreases.

We simulate two identical human pseudobulk matrices with; $p = 300$, $n = n_{ctrl} + n_{case} = 10$, $B = 5$, and $n_{ctrl} = n_{case}$. Note that model's number of features is $B \times p = 1500$. However, one pseudobulk matrix will have negative effects for all cell-types and genes $\boldsymbol{\mu}_{ctrl} = 10000 > 8705.5 = \boldsymbol{\mu_{case}}$ and the other all positive effects $\boldsymbol{\mu}_{ctrl} = 10000 > 11486.9 = \boldsymbol{\mu_{case}}$. Note that human change in $\log_2 FC$ is defined by:

$$\log_2 FC = \log_2 \left(\frac{\boldsymbol{\mu_{case}}}{\boldsymbol{\mu_{ctrl}}} \right) \tag{S10}$$

For the positive effect case the $\log_2 FC = 0.20$ while for the negative effect case $\log_2 FC = -0.20$.

We simulate the disease model $\log_2 FC$ drawing from $[a = 0, b = 1]$ interval with steps of 0.05. We report its midpoint value for each interval step as the average $\log_2 FC$. We simulate all genes to be statistically significant $\pi = 1$.

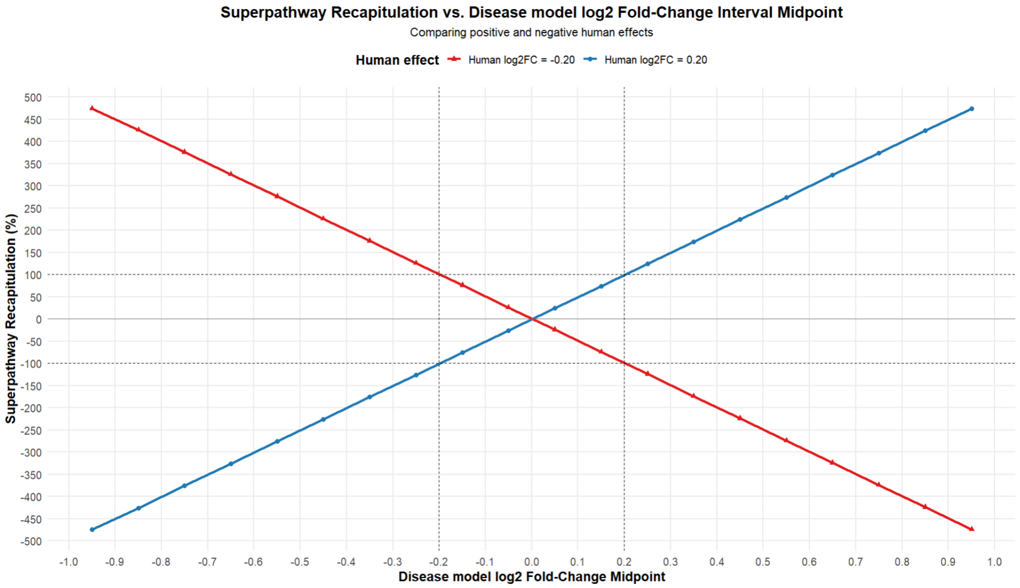

Fig. S1. Superpathway recapitulation against disease model $\log_2 FC$ midpoint. The Human $\log_2 FC = -0.20$ red curve corresponds to the simulated pseudobulk matrix with negative effects, while the Human $\log_2 FC = 0.20$ corresponds to the one with positive effects. Two vertical black dashed curves are shown at $\log_2 FC = 0.20$ and $\log_2 FC = -0.20$, while two horizontal black dashed curved are shown at Superpathway recapitulation values of -100% and 100%

For each disease model $\log_2 FC$ interval, we run singIST workflow against both two simulated pseudobulk human expression matrices, and we report its superpathway recapitulation against the interval midpoint disease model $\log_2 FC$.

In figure S1 we observe two key aspects of the superpathway recapitulation properties:

- For the recapitulation to be positive the sign of the human and disease model $\log_2 FC$ must coincide. Otherwise, the recapitulation is negative when there's a mismatch in direction of the $\log_2 FC$.

- For the Human $\log_2 FC = 0.20$ effect, a superpathway recapitulation of 100% is achieved when disease model $\log_2 FC \approx 0.20$, while a recapitulation of -100% is also achieved when $\log_2 FC \approx -0.20$. On the other side, for the human $\log_2 FC = -0.20$ effect, the 100% recapitulation is achieved when the disease model $\log_2 FC \approx -0.20$ while the -100% recapitulation is achieved when $\log_2 FC \approx 0.20$. Note that as the disease model effect becomes larger with respect to the human effect, the recapitulations increase beyond $\pm 100\%$ values. When the disease model has no effect the recapitulations become null.

B.2. One-to-one orthology effect on recapitulation

We randomly drop a fraction $f \in [0, 1]$ of genes from the fold-change list $\mathcal{L}$ and we recompute the recapitulations with singIST library. We simulate the disease model with a fixed-fold change interval $\log_2 FC \sim Uniform(0.19, 0.21)$ and map it against the simulated human pseudobulk matrix of positive effect. With increasing f, we illustrate that loss of putative one-to-one orthologs proportionally degrades recapitulation power, until it drops to 0 when $f = 0$.

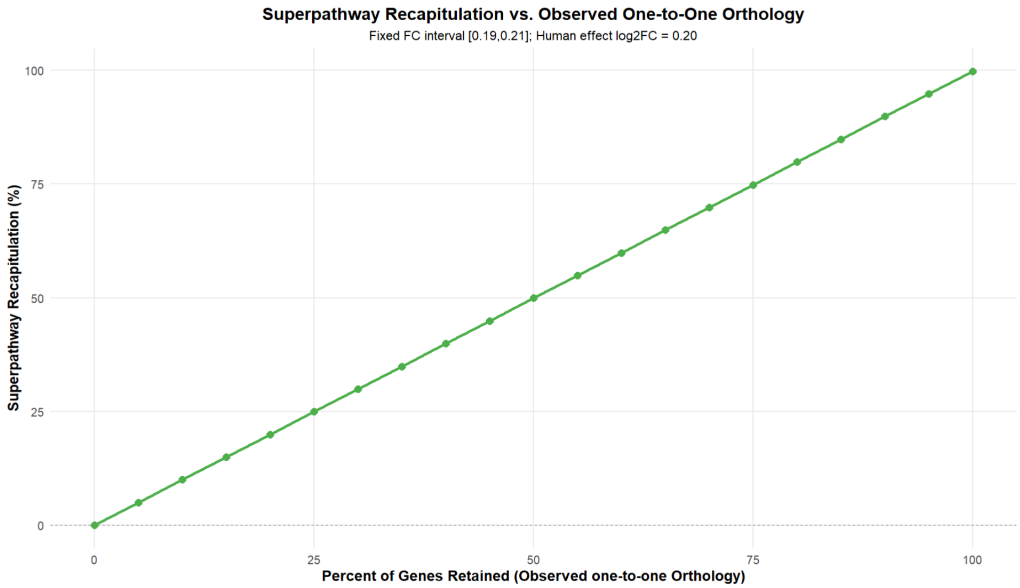

Fig. S2. Superpathway recapitulation against observed one-to-one orthology fraction $f \in [0, 1]$.

B.3. Cell-type mapping effect on recapitulation

We use the same inputs as before. However, now we vary the number of mapped cell-types in the class `mapping.organism` object. We consider $k = 1, 2, \ldots, B$, with $B = 5$, cell-types being mapped and recompute the recapitulations. Note that singIST workflow does not operate when zero cell-types are mapped. Recapitulation increases monotonically with k, demonstrating that multi-block integration in singIST effectively leverages additional cell-type information. Note that the increases are monotonic since the human effect is equal for all cell-types in the simulation.

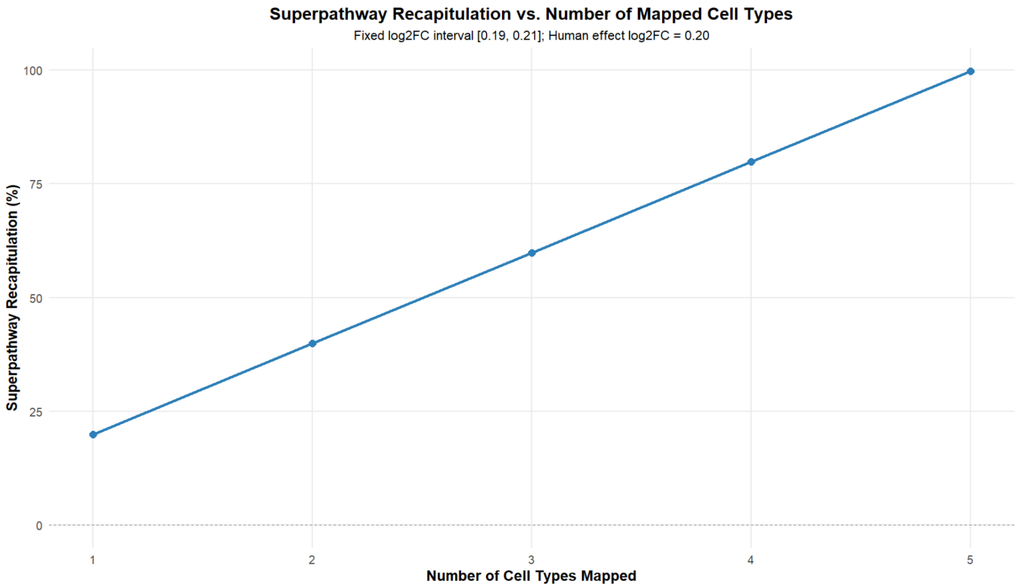

Fig. S3. Superpathway recapitulation for a disease model against number of cell-types mapped.

B.4. Proportion of DEGs effect on recapitulation

We simulate the disease model with a fixed-fold change interval $\log_2 FC \sim Uniform(0.19, 0.21)$, we vary the proportion π of genes marked as significant ($p_{\text{adj}} \leq 0.05$) and we use singIST workflow against the positive effect human pseudobulk matrix. The superpathway recapitulation grows with π, confirming that singIST sensitivity scales with the prevalence of differential signal.

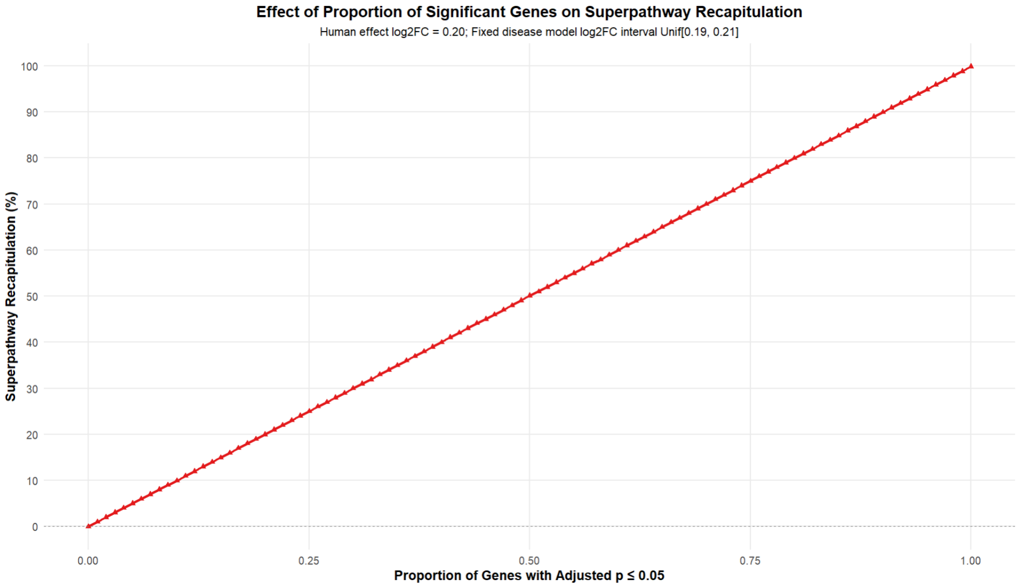

Fig. S4. Superpathway recapitulation for a disease model with $\log_2 FC \sim Uniform(0.19, 0.21)$ and $\pi \in [0, 1]$ proportion of statistically significant genes.

B.5. 100% recapitulation is achieved when human and disease model log2FC agree in direction and magnitude

Here we showcase via simulation the sign and magnitude of the recapitulation when magnitude and direction of log2FC perfectly match and perfectly oppose.

When there's a perfect match $\log_2 FC$ Human $= \log_2 FC$ Disease model the superpathway recapitulation lies around a constant value 100%. However, when there's perfect mismatch $\log_2 FC$ Human $= -\log_2 FC$ Disease model the superpathway recapitulation lies around a constant value -100%.

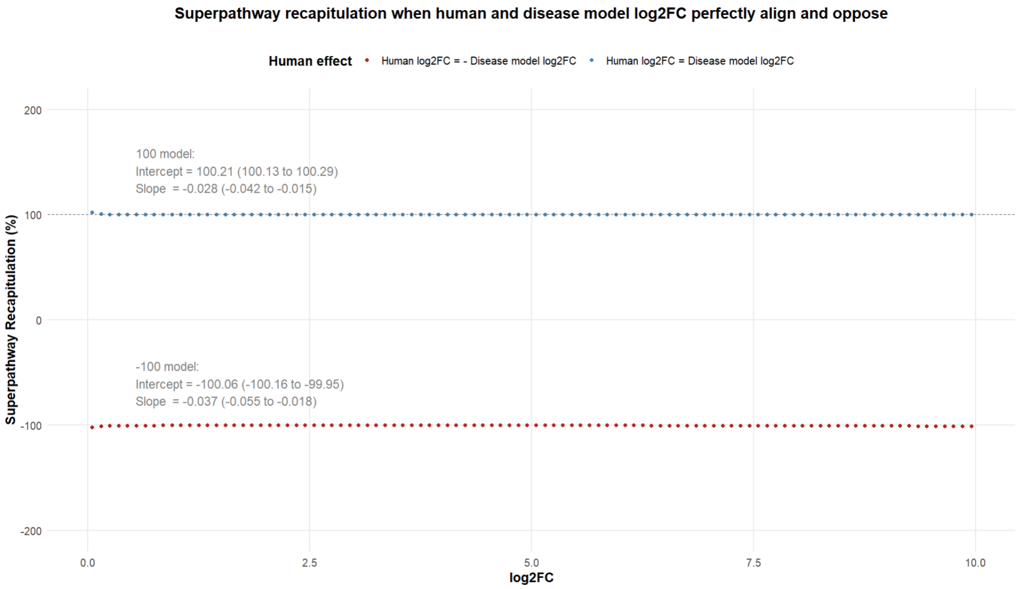

Fig. S5. Superpathway recapitulation when log2FC of human and disease model perfectly match and perfectly oppose.

2. BENCHMARKING SINGIST AGAINST OVERLAPPING DIFFERENTIALLY EXPRESSED GENES

We compare singIST to a more classical overlap of differentially expressed genes (ODEGs) strategy, under a controlled simulation setting with two disease models. Throughout this section we assume the human reference is fixed as in Section 1, and we consider two candidate disease models, denoted ℓ_1 and ℓ_2. We will use notation of section 1 if not stated otherwise. For the ODEG-based approach, let S_H be the set of human differentially expressed genes (DEGs) at the superpathway under study, and let S_ℓ be the set of DEGs in disease model $\ell \in \{\ell_1, \ell_2\}$. A natural scalar summary of disease model-to-human similarity for ODEGs is the size of the overlap $|S_\ell \cap S_H|$.

Within this framework, we say that model ℓ_2 is ODEG-superior to model ℓ_1 if:

$$|S_{\ell_2} \cap S_H| > |S_{\ell_1} \cap S_H| \tag{S11}$$

By contrast to ODEGs, singIST operates at the level of a superpathway $\mathcal{P}^p = \bigcup_{b=1}^{B} \mathcal{G}_p^b$, jointly integrating all relevant cell types b and the corresponding gene sets $\mathcal{G}_p^b$ defined in Section 1. We say that model ℓ_2 is singIST-superior to model ℓ_1 if:

$$\widehat{\Omega} f_{\ell_2} > \max\{0, \widehat{\Omega} f_{\ell_1}\} \tag{S12}$$

that is, if ℓ_2 recapitulates the human superpathway more strongly than ℓ_1, and is non-negative.

Beyond this quantitative benchmark, there are qualitative differences between ODEGs and singIST that motivate the comparison. In typical single-cell analyses, ODEGs are computed separately for each cell type, yielding cell type specific DEG sets S_H^b and S_ℓ^b and overlaps $|S_\ell^b \cap S_H^b|$ that are interpreted in isolation. This approach does not explicitly integrate information across cell types, treats all DEGs as equally informative, and is insensitive to the direction and magnitude of the $\log_2 FC$ beyond the binary DEG/non-DEG decision. In contrast, singIST is formulated directly at the superpathway level, aggregating evidence across all modeled cell types $\{C^b\}_{b=1}^{B}$, and combining these with disease model $\log_2 FC$ in a signed recapitulation $\widehat{\Omega} f_\ell$.

In the remainder of this section, we use two disease model setup (ℓ_1, ℓ_2) to quantify how often singIST and ODEGs agree in identifying the superior model (Eq. (S11)-Eq. (S12)), and to characterize the scenarios in which they disagree. We pay particular attention to disagreements driven by: direction of change, magnitude of change, and the distribution of signal across genes with different importance in the human model.

A. Agreement between singIST and ODEGs outcomes

We consider an idealized setting in which both ODEGs and singIST are expected to agree on which disease model best recapitulates the human condition. We fix a single simulated superpathway with p = 300 genes and B = 5 cell types, and construct a human reference pseudobulk as in Section 1, with $n_{case} = n_{ctrl} = 5$ and a homogeneous positive effect. For all genes and cell types we set $\mu_{ctrl} = 10000$ and $\mu_{case} = 10000 \cdot 2$, so that all genes in the superpathway have $\log_2 FC = 1$ in humans. This defines a human DEG set of S_H equal to the entire gene set $\mathcal{G}_p$.

We then define for the disease models, ℓ_1 and ℓ_2, that differ only in the proportion of genes declared as DEG, while perfectly matching the human effect ($\log_2 FC = 1$) for those DEGs. For ℓ_1, we select a random subset $S_{\ell_1} \subset \mathcal{G}_p$ of size $|S_{\ell_1}| = \pi_{\ell_1} p$ with $\pi_{\ell_1} = 0.5$, assign $\log_2 FC = 1$ and $p_{adj} \leq 0.05$ to genes in S_{ℓ_1}, and $\log_2 FC = 0$ for $p_{adj} > 0.05$.

In summary, this simulation confirms that singIST and ODEGs behave consistently when the singal is simple and globally homogeneous. When both disease models match the human $\log_2 FC$ pattern and differ only in the proportion of affected genes ($\pi_{\ell_1} = 0.5, \pi_{\ell_2} = 1$), both approaches correctly rank ℓ_2 as the superior model. This agreement case acts as a sanity check, showing that singIST reduces to the same qualitative preference as a standard ODEGs when only the prevalence of concordant DEGs changes.

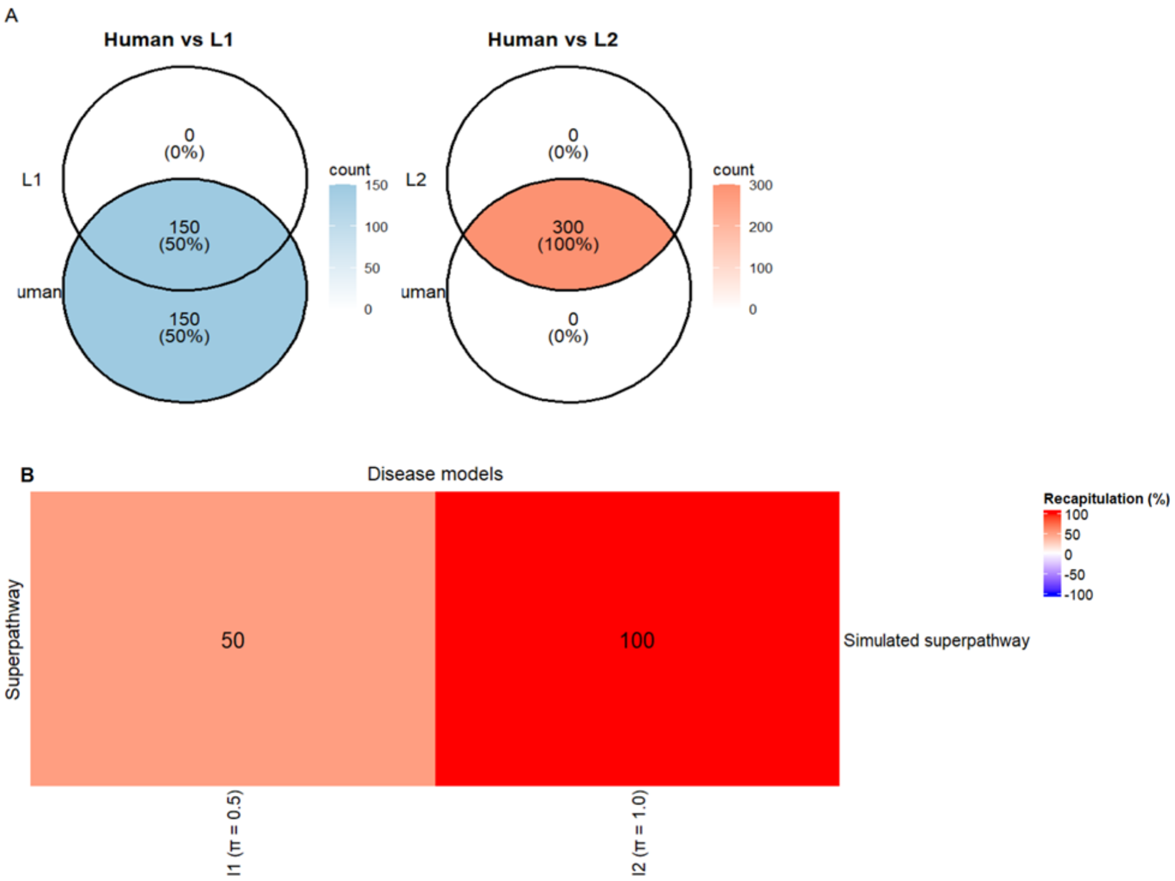

Fig. S6. Agreement between singIST and ODEGs in an idealized simulation scenario. A) Venn diagrams comparing the overlap of DEGs between human and each disease model. All genes in the simulated superpathway are differentially expressed in humans with $\log_2 FC = 1$. Model ℓ_1 is constructed so that only $\pi_{\ell_1} = 0.5$ of all genes are DEGs (right circle), whereas model ℓ_2 has $\pi_{\ell_2} = 1$, i.e all genes are DEGs and fully overlap with the human DEG set. The areas of the overlaps illustrate that $|S_{\ell_2} \cap S_H| > |S_{\ell_1} \cap S_H|$, so that ℓ_2 is ODEG-superior to ℓ_1 according to Equation Eq. (S11). **B)** Superpathway recapitulation values $\widehat{\Omega} f_\ell$ from singIST for the same two models. Under perfect one-to-one orthology and cell type mapping, and with all model DEGs matching the human direction and magnitude ($\log_2 FC = 1$), singIST yields an intermediate positive recapitulation for model ℓ_1 and a perfect positive recapitulation for ℓ_2. This satisfies $\widehat{\Omega} f_{\ell_2} > \max\{0, \widehat{\Omega} f_{\ell_1}\}$, so that ℓ_2 is also singIST-superior to ℓ_1 according to Eq. (S12).

B. Disagreement due to direction of change

We repeat the same setting as in 2.A, except for one change: both disease models now invert the human direction of change. The human reference still has $\log_2 FC = 1$ for all genes in the simulated superpathway, with $\pi_{\ell_1} = 0.5$ and $\pi_{\ell_2} = 1$ defined over all genes, but we now assign $\log_2 FC = -1$ to DEGs in both ℓ_1 and ℓ_2. As a consequence, the ODEG overlaps remain unchanged and still favour ℓ_2, because sign is ignored and only the presence of DEGs is counted. In contrast, singIST recapitulation becomes negative for both models, neither model is considered superior, proving that singIST can penalize direction of change mismatches that ODEG-based entirely overlooks.

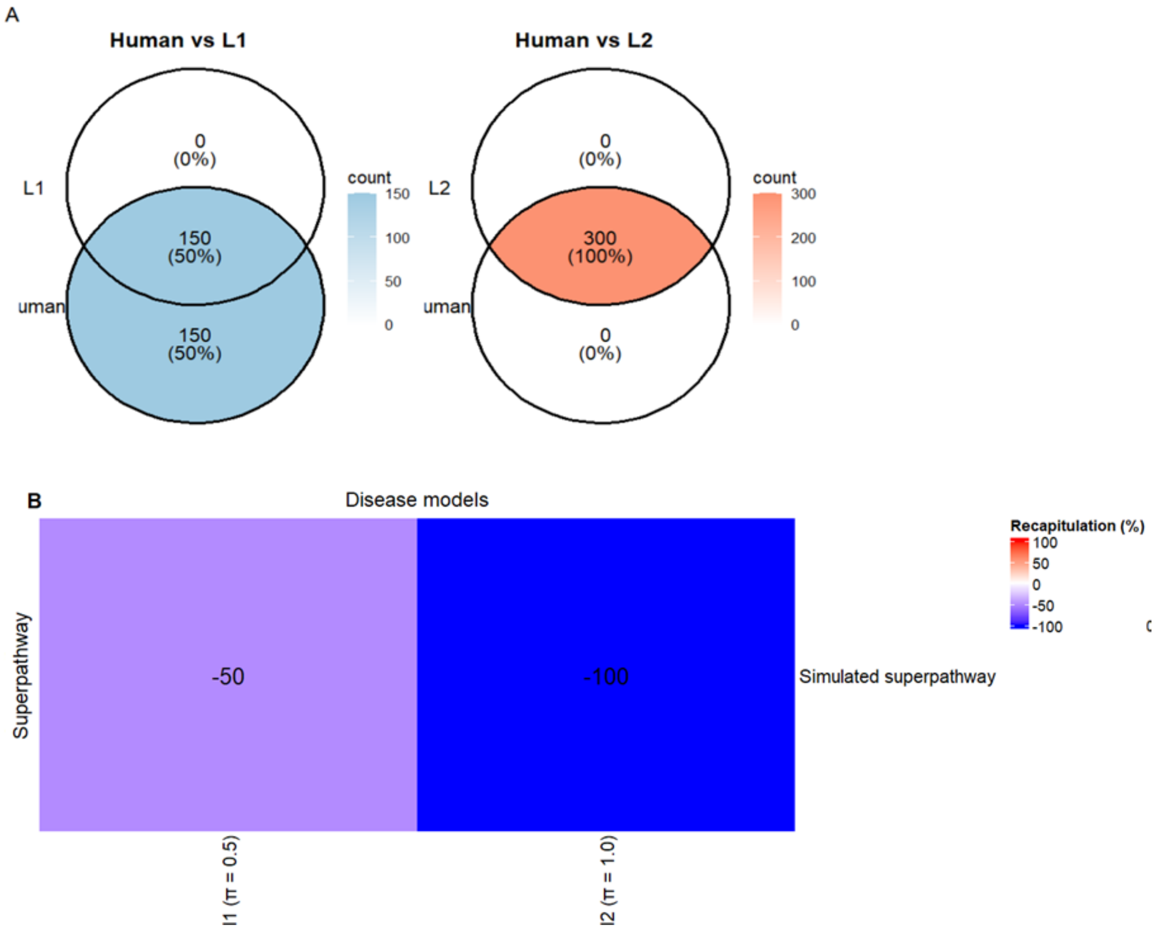

Fig. S7. Disagreement between singIST and ODEGs when disease models invert the human direction of change. A) Venn diagrams of DEGs between human and each disease model, as in the agreement scenario: model ℓ_1 has $\pi_{\ell_1} = 0.5$ and model ℓ_2 has $\pi_{\ell_2} = 1$, so $|S_{\ell_1} \cap S_H| > |S_{\ell_2} \cap S_H|$. Because ODEGs ignore the sign of the $\log_2 FC$, this panel is identical to the previous case and still favours ℓ_2. **B)** singIST superpathway recapitulation when both models have $\log_2 FC = -1$ for their DEGs, opposite to the human $\log_2 FC = 1$. Recapitulation values are negative for both models, indicating systematic disagreement with the human pattern. Under the singIST superiority criterion, neither model is considered superior $(\widehat{\Omega}f_{\ell_1}, \widehat{\Omega}f_{\ell_2} < 0)$, highlighting a direction of change mismatch that is not captured by the ODEG.

C. Disagreement due to magnitude of change

In this scenario we only modify the magnitude of the disease-model changes. The human reference is the same as in the agreement case (all genes with $\log_2 FC = 1$), and both models share the same direction of change, but differ in DEG proportion and effect size: ℓ_1 has $\pi_{\ell_1} = 0.5$ and $\log_2 FC = 1$ for its DEGs, whereas ℓ_2 has more DEGs ($\pi_{\ell_2} = 0.7$) but a much smaller effect ($\log_2 FC = 0.1$). ODEGs, which only counts overlapping DEGs, still favors ℓ_2 because $|S_{\ell_2} \cap S_H| > |S_{\ell_1} \cap S_H|$. By contrast, singIST assigns a substantially higher recapitulation to ℓ_1 ($\approx 50\%$) than to ℓ_2 ($\approx 7\%$), reflecting that ℓ_1 not only overlaps with the human DEG set but also better matches the human effect magnitude. This illustrates that singIST can distinguish between models with similar DEG overlaps but very different biological impact, whereas ODEGs cannot.

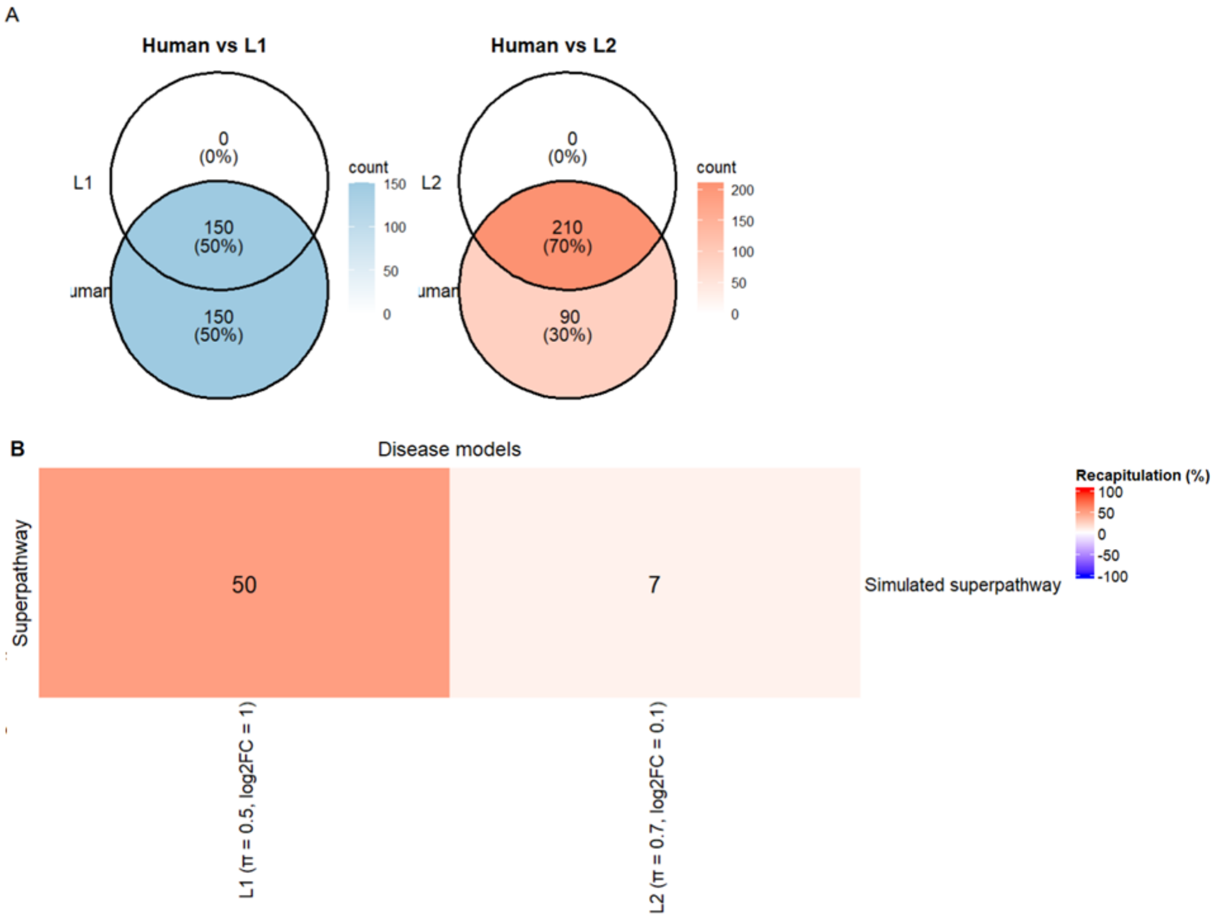

Fig. S8. Disagreement between singIST and ODEGs due to effect-size magnitude. A) Venn diagrams for the overlap of DEGs between human and each disease model. All genes in the simulated superpathway are differentially expressed in humans with $\log_2 FC = 1$. Model ℓ_1 has $\pi_{\ell_1} = 0.5$ of DE genes with $\log_2 FC = 1$, whereas model ℓ_2 has a larger DEG proportion $\pi_{\ell_2} = 0.7$ but much smaller effects ($\log_2 FC = 0.1$). Because ODEGs only uses the size of the overlap $|S_\ell \cap S_H|$, it ranks ℓ_2 as superior to ℓ_1. **B)** Superpathway recapitulation $\widehat{\Omega} f_\ell$ from singIST for the same models. Under perfect orthology and cell type mapping, singIST yields a higher positive recapitulation for ℓ_1 ($\approx 50\%$) and a near null recapitulation for ℓ_2 ($\approx 7\%$), identifying ℓ_1 as the better model once the magnitude of $log_2 FC$ is taken into account.
